# Craniomandibular osteology of a new massopodan sauropodomorph (Dinosauria: Sauropodomorpha) from the Late Triassic (latest Norian) of Canton Aargau, Switzerland

**DOI:** 10.1186/s13358-025-00373-6

**Published:** 2025-07-14

**Authors:** Alessandro Lania, Ben Pabst, Torsten M. Scheyer

**Affiliations:** 1 Abteilung Paläontologie, Bonner Institut für Organismische Biologie, Rheinische Friedrich-Wilhelms-Universität Bonn, Nussallee 8, 53115 Bonn, Germany; 2 Sauriermuseum Aathal, Zürichstrasse 69, 8607 Aathal-Seegräben, Switzerland; 3 Department of Paleontology, University of Zurich, Karl-Schmid-Strasse 4, 8006 Zurich, Switzerland

**Keywords:** Sauropodomorpha, Plateosauridae, Massopoda, Late Triassic, Klettgau formation, Gruhalde member, Craniomandibular osteology, Mosaic craniomandibular anatomy, Phylogeny, Micro-computed tomography

## Abstract

Non-sauropodan sauropodomorphs represented the most abundant and diverse herbivore component of the Gondwanan continental paleoecosystems during the Late Triassic. Nonetheless, a constantly increasing diversity has been recovered also from Laurasian formations, such as the Klettgau Formation, which is best exposed at the Gruhalde clay pit (Tonwerke Keller AG) in Frick, Canton Aargau, Switzerland. Despite being renowned for mass-accumulation horizons of the plateosaurid *Plateosaurus trossingensis*, a new fossiliferous layer was recently discovered above the “*Plateosaurus* bonebeds”, yielding the holotype of the neotheropod *Notatesseraeraptor frickensis* as well as several partial articulated skeletons of an unknown sauropodomorph. The complete craniomandibular anatomy of an articulated skull, SMF 13.5.37, belonging to a partial skeleton, SMF 13.5, referred to this new latest Norian sauropodomorph from the Klettgau Formation is here presented. Micro-computed tomography scans (µCT) as well as segmentation techniques were employed in order to examine inaccessible craniodental features of the snout of the specimen under study. The osteological investigation and the anatomical comparison with related taxa unveiled a unique mosaic-like combination of plesiomorphic and apomorphic craniomandibular features, implying that the cranial anatomy of SMF 13.5.37 is transitional between non-massopodan plateosaurian and massopodan sauropodomorph morphologies, similarly to the Argentinian *Coloradisaurus brevis* from the mid-to-late Norian of the Los Colorados Formation. An intermixed craniomandibular condition is also reflected in the phylogenetic results, which resolve SMF 13.5.37 as a basal massopodan, branching out either at the first or third node of Massopoda, representing the first Laurasian non-sauropodiform massopodan. Even though the evolutionary trend towards a complete massopodan-like architecture needs to be further tested with the study of the postcranium, SMF 13.5.37 unequivocally represents the skull of a new massopodan sauropodomorph taxon from Switzerland, shedding light on a more diversified herbivorous dinosaurian paleofauna from the Norian comparable to those of South America and Africa, as it represents the fourth officially recognized non-sauropodan sauropodomorph along with *Plateosaurus trossingensis*, *Gresslyosaurus ingens* and *Schleitheimia schutzi*.

**Supplementary Information:**

The online version contains supplementary material available at 10.1186/s13358-025-00373-6.

## Introduction

Among the Mesozoic terrestrial vertebrate groups, Sauropodomorpha represents one of the most successful dinosaurian clades, as it became one of the most abundant and dominant herbivore components of both the Late Triassic and the Jurassic continental paleoecosystems with an almost global distribution, spatially spanning from Antarctica to Greenland (e.g. Apaldetti et al., 2021; Beccari et al., 2021; Galton & Upchurch, 2004; Pol et al., 2021; Smith & Pol, 2007). The origin of sauropodomorphs dates back to the early Late Triassic of Gondwanan continents with the oldest representatives discovered in the Carnian sediments of the Santa Maria Formation (southern Brazil), the Ischigualasto Formation (northwestern Argentina) (e.g. Cabreira et al., 2016; Langer et al., 2022; Pol et al., 2021), the Pebbly Arkose Formation (southern Africa) (Griffin et al., 2022) and the Laurasian Popo Agie Formation (North America) (Lovelace et al., 2025).

Based on the South American fossil record, which provides one of the most comprehensive understandings of the early evolution of Sauropodomorpha, a rapid radiation and diversification occurred in a timeframe of approximately 30 million years, shifting from a limited number of lineages characterized by a small body size, bipedal locomotion and carnivorous/faunivorous dietary habits (e.g. Cabreira et al., 2016; Müller et al., 2018a; Sereno et al., 2013), all of which typical of Carnian taxa like *Eoraptor lunensis* Sereno et al., 1993 and *Buriolestes schultzi* Cabreira et al., 2016, to a plethora of new sauropodomorphs during the Norian-Rhaetian, like *Macrocollum itaquii* Müller et al., 2018b, *Riojasaurus incertus* Bonaparte, 1967 and *Coloradisaurus brevis* Bonaparte, 1979 accounting for medium-to-large size body plans, onset of quadrupedality and acquisition of herbivorous diet (e.g. Apaldetti et al., 2014; Barrett et al., 2010; Müller, 2020; Pol et al., 2021; Rauhut et al., 2011). Additionally, this dramatic increase in the sauropodomorph paleobiodiversity of southern Pangea at Norian times is further attested by the emergence of new main lineages, such as Massopoda and Sauropodiformes, as well as by a notable divergence in the morphological disparity, which is consequently reflected in an expansion of the occupied morphospace given the development of novel anatomical features (e.g. Apaldetti et al., 2021; Ballell et al., 2022; Button et al., 2017).

Although not being as much diverse as the Gondwanan paleofaunas, a comparable taxonomic assemblage of sauropodomorphs has been documented from the Norian-Rhaetian outcrops of Europe, especially Switzerland, Germany and England, with numerous taxa erected since the first half of the nineteenth century (e.g. Riley & Stutchbury, 1836; Meyer, 1837; Huene, 1926, 1928, 1932; Sander, 1992). The European sauropodomorphs are mainly represented by several non-plateosaurian sauropodomorphs, like *Thecodontosaurus antiquus* Morris, 1843, *Pantydraco caducus* Galton et al., 2007 and *Efraasia minor* Huene, 1908, and the plateosaurid *Plateosaurus trossingensis* Fraas, 1913 (previously known as *Plateosaurus engelhardti*, ICZN, 2019) (e.g. Rauhut et al., 2020; Regalado Fernández & Werneburg, 2022). Nevertheless, new information about a broader dinosaurian paleobiodiversity during the Norian stage has been revealed by the redescription and revaluation of specimens that were previously assigned to the latter taxon, comprising also the first non-*Plateosaurus* plateosaurid to have reached paleolatitudes over 40°N, namely *Issi saaneq* Beccari et al., 2021 from Greenland, and the first two Norian non-sauropodan sauropodiforms of Europe, precisely *Schleitheimia schutzi* Rauhut et al., 2020 from Switzerland and *Tuebingosaurus maierfritzorum* Regalado Fernández & Werneburg, 2022 from Germany, which even predate the only other known basal sauropodiform *Camelotia borealis* Galton, 1985a, 1985b that comes from the Rhaetian of England (Beccari et al., 2021; Galton, 1998a; Rauhut et al., 2020). However, the evolutionary pattern shown by European sauropodomorphs from the Late Triassic does not completely overlap with that of the Gondwanan fossil record due to the absence of evidence of several lineages, such as taxa belonging to the basal branches of Massopoda or Massospondylidae. Accordingly, a morphological and evolutionary gap is yet to be filled between the European non-massopodan sauropodomorphs, such as plateosaurids, and the more derived non-sauropodan sauropodiforms, like *Sch. schutzi*.

Among the European taxa, the plateosaurid *Plateosaurus trossingensis* is the most renowned and best-known Late Triassic non-sauropodan sauropodomorph as it accounts for a fossil record comprising dozens of partial-to-complete skeletons from different localities, occurring in Norian aged outcrops of Germany, Switzerland, France, Norway and Greenland (e.g. Galton & Upchurch, 2004; Galton, 1998b, 2001; Hurum et al., 2006; Jenkins et al., 1994; Sander, 1992; Schaeffer, 2024). Plenty of studies have been conducted highlighting its osteohistological features, mainly consisting of a poor correlation between ontogenetic age and morphometric size, named developmental plasticity (Klein & Sander, 2007; Sander & Klein, 2005), that is shared with the massospondylid *Massospondylus carinatus* Owen, 1854 (Chapelle et al., 2021) and the sauropodiform *Mussaurus patagonicus* Bonaparte & Vince, 1979 (Cerda et al., 2022), and a remarkable degree of morphological variation among the referred specimens (e.g. Lallensack et al., 2021; Lefebvre et al., 2020; Nau et al., 2020; Prieto-Márquez & Norell, 2011; Rauhut et al., 2020). Although many additional species were established over time (e.g. Beccari et al., 2021; Lallensack et al., 2021; Nau et al., 2020; Prieto-Márquez & Norell, 2011; Schaeffer, 2024), the current consensus is that a single type species is validly accepted, namely *Plateosaurus trossingensis* based on the holotype SMNS 13200 (Huene, 1926; ICNZ, 2019; Schaeffer, 2024), pending proper redescriptions of the material referred to taxa like *Sellosaurus gracilis* Huene, 1908 and *Gresslyosaurus ingens* Rütimeyer, 1856, formerly considered additional species of *Plateosaurus* (e.g. Yates et al., 2010; Yates, 2003a). Even though the taxonomic validity as different *Plateosaurus* species is debatable, both *Sel. gracilis* and *Gre. ingens* substantially differ in age from *Pla. trossingensis*, being recovered from Norian formations that are stratigraphically older and younger respectively (Mujal et al., 2025; Rauhut et al., 2020; Yates, 2003a). Accordingly, the chronostratigraphic distribution of non-*Pla. trossingensis* taxa in Laurasia reflects a differential taxonomic diversity of Sauropodomorpha throughout the Late Triassic, supporting the definition of rapid biotic turnovers and the establishment of new clades especially during the Norian.

Interestingly, the vast majority of *Plateosaurus trossingensis* specimens have been yielded by almost monospecific mass accumulations from the German localities of Halberstadt and Trossingen and the Swiss one of Frick (e.g. Galton, 1986; Lallensack et al., 2021; Nau et al., 2020; Schaeffer, 2024), generally mentioned as “*Plateosaurus* bonebeds” given the peculiar taphonomic pattern that is consistent across the different sites (*sensu* Sander, 1992). The fossil locality of Frick (Canton Aargau, Switzerland) has been known since 1961 when Ernst Wälchli discovered the first bone fragment (Sander, 1992). Given its significant lateral distribution of the bone-bearing layers, which reaches roughly 4 km in beeline indicating a potential fossiliferous surface distribution on the order of square kilometers, and its massive production of Late Triassic dinosaurian material, which has been systematically extracted since 1976 (Foelix et al., 2011; Nau et al., 2020), this fossil locality represents a unicum of paleontological interest in Europe. The Norian strata that have been deeply investigated, returning most of the *Plateosaurus trossingensis* specimens now housed at the Sauriermuseum Frick (SMF), extensively outcrop at the Gruhalde clay pit (Tonwerke Keller AG) and belong to the middle fossiliferous horizons of the Gruhalde Member of the Klettgau Formation (Jordan et al., 2016). Nonetheless, fieldwork efforts led to the discovery in 2006 of an upper fossiliferous horizon within the Gruhalde Member, placed 6 m above the “*Plateosaurus* bonebed” layers and roughly 60 cm below the unconformably overlying Jurassic sediments of Staffelegg Formation, and from which the holotype of the neotheropod *Notatesseraeraptor frickensis* Zahner & Brinkmann, 2019 was recovered (Lallensack et al., 2021; Tschopp et al., 2020; Zahner & Brinkmann, 2019). Notably, approximately 1000 m^2^ were prospected between 2006 and 2013, resulting in the discovery of additional findings including rhynchocephalian remains as well as eight semiarticulated skeletons, accounting for different completeness degrees, of a sauropodomorph which, at first glance, did not correspond to *Plateosaurus trossingensis*, as already pointed out by Lallensack et al. (2021), and thus potentially indicating a more diverse dinosaurian paleofauna than previously expected.

Here we thoroughly describe for the first time the craniomandibular anatomy of the specimen SMF 13.5.37, a skull pertaining to a fairly complete skeleton, SMF 13.5, belonging to a new sauropodomorph from the uppermost fossiliferous layer of the Gruhalde Member of the Klettgau Formation from Frick (Switzerland). Extensive comparisons with other non-sauropodan sauropodomorphs and several rounds of cladistic analysis are performed in order to elucidate its taxonomic status. Finally, we discuss its macroevolutionary implications depending on the resulting phylogenetic affinities.

## Geological context

**Fig. 1:**
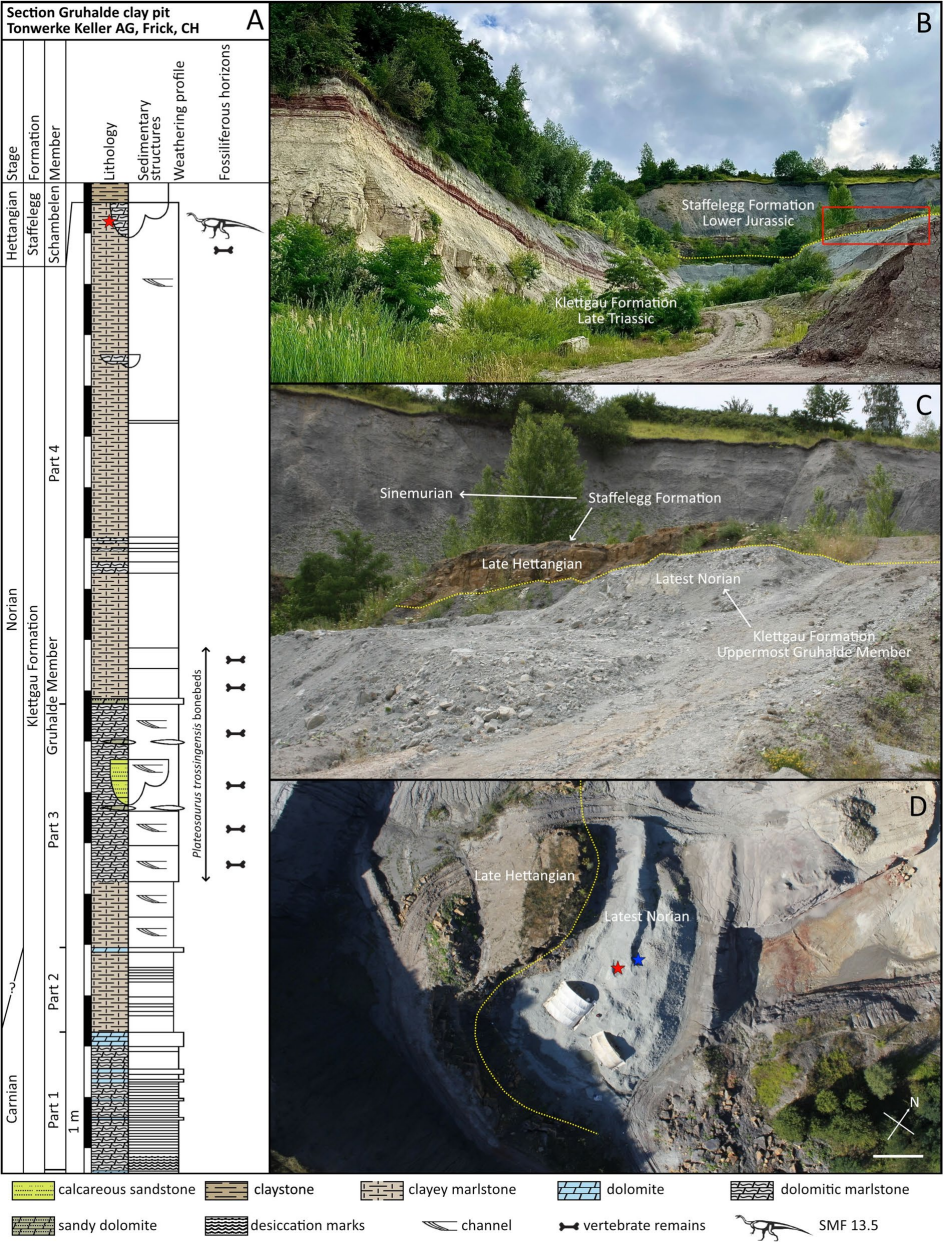
Geological setting of the Gruhalde Member, Klettgau Formation at the Gruhalde clay pit (type locality), Tonwerke Keller AG in Frick, Switzerland and overview of the find of SMF 13.5. Yellow dashed line marks the Triassic/Jurassic boundary between the Klettgau Formation and the Staffelegg Formation. Red star indicates where SMF 13.5 was excavated. Blue star represents the finding site of *Notatesseraeraptor frickensis*. **A** Lithostratigraphic section of the Gruhalde Member based on the exposed outcrop at the type locality with stratigraphic position of SMF 13.5. Modified from Jordan et al. (2016). **B** Geology of the Gruhalde clay pit, illustrating the Triassic and Jurassic outcrops. **C** Close-up of the uppermost fossiliferous horizon of the fourth subunit of the Gruhalde Member, where SMF 13.5 comes from, representing the same exposed outcrops highlighted by the red rectangle in **B**. **D** Aerial overview of the fourth subunit of the Gruhalde Member, showing the exact position where SMF 13.5 was found and its close association with the holotype of *Not. frickensis*. Scale bar equals 5 m. The aerial image was taken in 2013 by BP

The Klettgau Formation is one of the most extensive stratigraphic successions of the Late Triassic in Europe, consisting of a lithologically heterogenous series deposited over a prolonged timeframe of 26–30 million years, from the Early Carnian to the Late Rhaetian (DSK, 2002; Jordan et al., 2016). Outcropping in many localities across Switzerland, the Klettgau Formation records a non-continuous sequence of variegated playa sediments with fluvial and marine influence, depicting various lateral paleoenvironmental shifts over the entire stratigraphic section (Jordan et al., 2016). Within the Klettgau Formation, six successive and conformable lithostratigraphic units occur (Jordan et al., 2016), among which the fourth, officially recognized as Gruhalde Member and previously known as “Obere Bunte Mergel”, yields Norian (227–208.5 Ma) strata plentifully enriched in vertebrate remains, especially belonging to the early sauropodomorph *Pla. trossingensis* (Fig. 1A) (e.g. Lallensack et al., 2021; Nau et al., 2020; Sander, 1992; Tschopp et al., 2020).

Jordan et al. (2016) recognized the Gruhalde clay pit (Tonwerke Keller AG) in Frick (Canton Aargau), Switzerland, as the type locality of the Gruhalde Member, where a conformable succession of continental sediments, mostly variegated dolomitic marls, is widely exposed, reaching 20 m in thickness (Fig. 1B). Nonetheless, despite the remarkable chronological record of likely 20 Myr (Jordan et al., 2016; Sander, 1992), the stratigraphic section has temporal gaps given by non-homogeneous sedimentation rate, occasionally characterized by omission or erosion patterns.

The Gruhalde Member is further divided into four subunits (Jordan et al., 2016), the third and the fourth of which bear rich fossiliferous horizons but differ in lithology and diverse vertebrate assemblage (Fig. 1A). Specifically, the third subunit, which is comparable to the Norian Trossingen Formation and Löwenstein Formation, accounts for 5 m of non-continuous greyish to purple dolomitic marl layers with sparse sandstone channel structures (Jordan et al., 2016; Lallensack et al., 2021; Tschopp et al., 2020). Within this subunit, which depicts a Norian paleoenvironment dominated by expanded terrestrial playa influenced by both possible marine or freshwater transgression and pedogenesis, an extensive dinosaurian fossil record is documented, mostly consisting of partial or complete, mostly articulated *Pla. trossingensis* skeletons, but also comprising medium-to-large theropod remains (e.g. Lallensack et al., 2021; Nau et al., 2020; Tschopp et al., 2020).

On the other hand, the fourth subunit of the Gruhalde Member consists of 10 m of greyish to purplish clayey marls with rare dolomitic channels, the top of which marks the upper boundary of the Klettgau Formation with the unconformably overlying Early Jurassic (Late Hettangian) Staffelegg Formation (Fig. 1C) (Jordan et al., 2016). The chronostratigraphic discontinuity with the Jurassic formation is due to either a sedimentary omission or erosion during the Rhaetian, which also hinders the identification of a clear boundary between the Norian and the Rhaetian in the last subunit of the Gruhalde Member, which is thus conservatively referred as mid-to-latest Norian in age (Jordan et al., 2016; Zahner & Brinkmann, 2019). Nonetheless, pending additional geochemical and chronostratigraphic investigations that will be conducted elsewhere, it is plausible to hypothesize a potential, as of yet untested, Rhaetian age for the uppermost section of the fourth subunit of the Gruhalde Member. Two distinct fossiliferous horizons, vertically separated by 6 m, are found respectively at the bottom and at the top of the fourth subunit. Specifically, the former records a similar faunal assemblage as the one found in the underlying third subunit, but more diversified, accounting not only for *Pla. trossingensis*, but also for the stem-turtle *Proganochelys quenstedtii* Baur, 1887 (Scheyer et al., 2022), whereas the latter yields the neotheropod *Not. frickensis*, in which rhynchocephalian remains were found, and several partial semi-to-articulated sauropodomorph skeletons (Tschopp et al., 2020; Zahner & Brinkmann, 2019). Among these, the skeleton SMF 13.5 was unearthed within the upper fossiliferous horizon of the fourth subunit of the Gruhalde Member, specifically within a range of 40–80 cm below the boundary with the overlying Jurassic Staffelegg Formation, making this specimen as the one found the closest to the Triassic-Jurassic transition in the entire stratigraphic section exposed in Frick.

Taphonomy-wise, the uppermost fossiliferous layer strikingly differs from all the underlying bone-bearing horizons in respect to the bone preservation, given a much lower degree of plastic deformation and different colouration, which is black, as already reported by Zahner and Brinkmann (2019) and Lallensack et al. (2021), and not orange-grey as the classic fossil material from Frick (e.g. Nau et al., 2020). This remarkable difference might be due to a different chemistry of both the paleoenvironmental setting and the fossilization process, however further sedimentological and chemical analyses are required to confirm it. A taphonomic bias is shown across all the sauropodomorph-bearing horizons of the entire stratigraphic section, consisting of a pattern leading to the preservation of the second half of the trunk region, pelvic girdle and hindlimbs of sauropodomorph dinosaurs in an upright posture (e.g. Sander, 1992; Tschopp et al., 2020). This condition has been interpreted as a specific paleoenvironmental setting related to mud-hole traps for large-sized organisms, which was also referred to the *Plateosaurus* taphonomy in Trossingen, Germany (Schoch & Seegis, 2014; Schaeffer, 2024). Despite most of the sauropodomorph specimens from the Gruhalde Member show this pattern, a vaster array of skeletons was recovered, accounting for more complete and articulated sauropodomorphs, lightly-built theropods and isolated non-dinosaurian bones as well and thus partially contradicting the previous taphonomic hypothesis (Tschopp et al., 2020).

## Material and methods

### Discovery, scan and photogrammetry of SMF 13.5.37

**Fig. 2:**
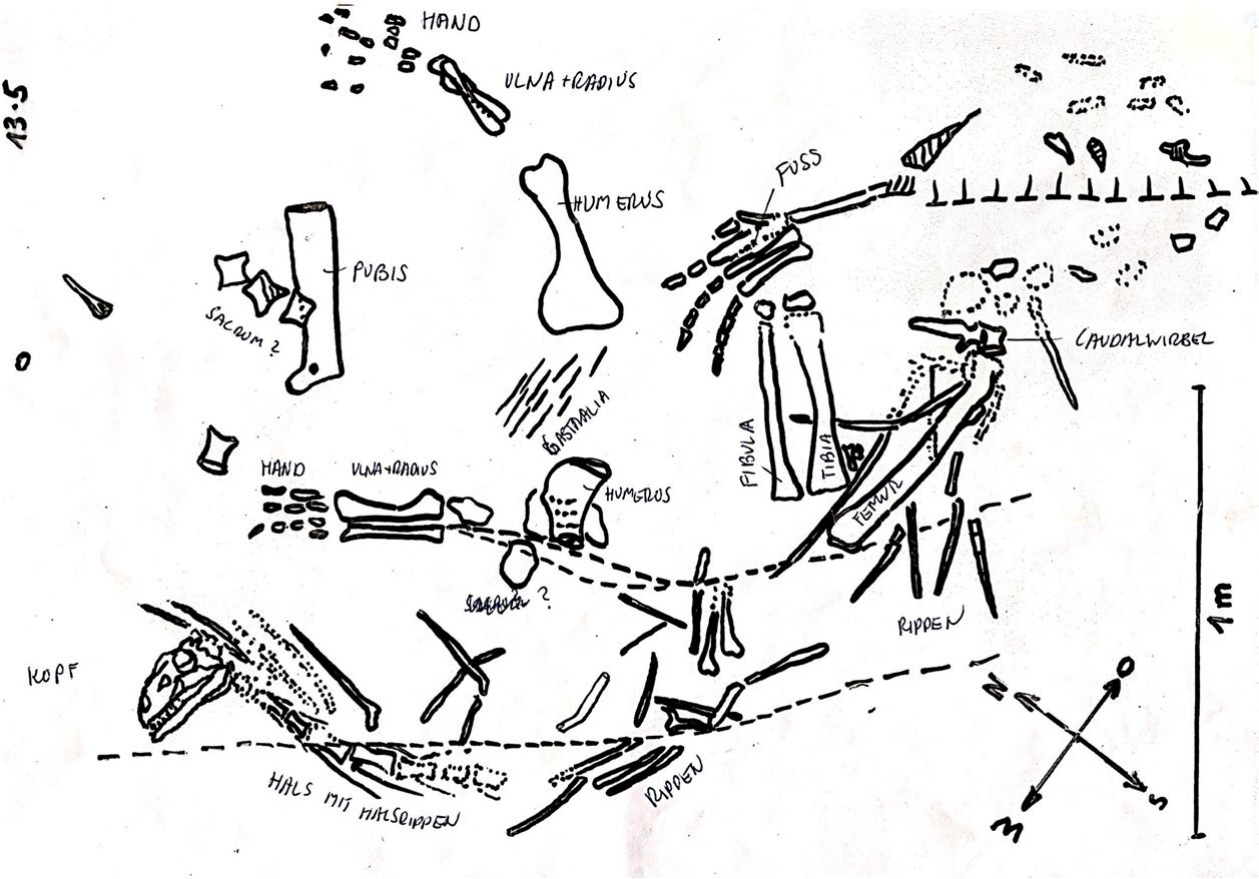
Scan of the excavation map of the specimen SMF 13.5 showing the disposition of the skeletal elements in situ. The original map was drawn in 2013 by BP

The material described herein consists of a complete skull, SMF 13.5.37, belonging to a partially complete skeleton, SMF 13.5 (Fig. 2), of an unknown sauropodomorph from the uppermost fossiliferous horizon of the Gruhalde Member (Klettgau Formation) from the Gruhalde Quarry of the Tonwerke Keller AG in Frick. Discovered in 2013 during the annual excavation campaign supervised by BP and Ursina Bachmann, the complete specimen SMF 13.5 was located three meters away from the holotype of *Not. frickensis* (Fig. 1D) (Zahner & Brinkmann, 2019) and close to other three partial sauropodomorph postcranial remains that likely belong to the same taxon. Specifically, the skull, SMF 13.5.37, was found lying on its right lateral side and westward oriented, still in articulation with the cervical vertebral series (Fig. 2). However, probably due to tectonic activity, the rostralmost portion of the snout, mostly accounting for the premaxillae, was recovered roughly 30 cm away and in line from the main skull block. Given the close association, the paucity of cranial material and the perfect matching between the two parts, the premaxillary block and the main skull block are referred to belong unambiguously to the same individual.

After the extraction, the complete specimen SMF 13.5 was stored in the vertebrate collection of the Sauriermuseum Frick (SMF) and then part of it, including SMF 13.5.37, was mechanically prepared with pneumatic air scribes by BP and Rabea Lillich at the preparation lab of the Sauriermuseum Aathal, Aathal, Switzerland. The preparation of the postcranium is yet to be completed and thus its osteological description will be provided separately in a further paper.

Given that the morphologies of the neurocranium, the palatal complex and the medial portions of the dermal cranial bones are obscured because of the encasing matrix, the specimen SMF 13.5.37 was scanned using a NIKON XTH 225 ST CT Scanner housed at the Anthropological Department of the University of Zurich, Switzerland. However, given the non-fitting dimension of the main skull block, only the smaller premaxillary block was investigated with a successful micro-computed tomography scan. The best µCT imaging was obtained with scan parameters equal to 145 kV in voltage and 354 µA in current, providing a voxel size of 0.06470 mm with no filter used. The resulting data dimensions are as follows: 1000 × 1000 × 1000 with VoxelSize = 0.06469785 mm. Virtual three-dimensional models of the premaxillae, the rostralmost portion of the dentaries and related teeth were subsequently created through segmentation using Avizo Software version 2023.2.

A second scanning session was conducted at Eurofins Qualitech AG, Mägenwil, Switzerland using an YXLON CT Modular Scanner on the main skull block of SMF 13.5.37. Although an image stack was produced from the micro-computed tomography scan, the density resolutions of both the matrix and the bone tissue were not distinguishable from one another, rendering the analysis results inapplicable for any further analysis.

A 3D photogrammetric model of SMF 13.5.37 was obtained with Agisoft Metashape 2.1.0, by processing 120 shots for each block and merging together the individual block models. The photos were shot with a Canon EOS 1000D.

Measurements of bones were manually taken with a calliper and a goniometer.

### Phylogenetic analysis

In order to investigate the phylogenetic affinities of SMF 13.5.37, the specimen was scored as a distinct operational taxonomic unit (OTU) in the data matrix of Ezcurra et al. (2024) (91 taxa and 421 morphological characters) using Mesquite v3.81 (Maddison & Maddison, 2023). Ordered characters were treated as such according to Ezcurra et al. (2024).

A revision of the scores for the cranial characters 50, 58 and 65 was conducted (see Supplementary material, “summary list of scoring amendments upon the phylogenetic matrix”). Furthermore, scores for the taxon *Plateosaurus trossingensis* were updated in the Ezcurra et al. (2024) matrix based on the osteological redescription of the holotype material by Schaeffer (2024). Finally, the taxon *Musankwa sanyatiensis* Barrett et al., 2024 was added in the Ezcurra et al. (2024) matrix in accordance with the character scoring of Barrett et al. (2024).

The data matrix was initially analysed with TNT 1.6 (Goloboff & Morales, 2023), specifying *Euparkeria* as the outgroup taxon and using equally weighted characters, performing Traditional Search as tree search strategy with 100 replicates of Wagner trees and TBR swapping algorithm, holding 1000 trees per replicate, and 1 random seed. Based on the resulting trees, a strict consensus tree was calculated. In order to resolve large polytomies, a second round of analysis was conducted employing the same search strategy with implied weighting, setting the concavity constant K value equal to 12 (Goloboff et al., 2018), from whose outputs a strict consensus tree was determined.

## Systematic paleontology

DINOSAURIA Owen, 1842

SAURISCHIA Seeley, 1887

SAUROPODOMORPHA Huene, 1932

MASSOPODA Yates, 2007

*Investigated material*: SMF 13.5.37, a complete articulated skull, comprising of cranium and mandible, with the left elements being slightly displaced ventrally from life position and weakly three-dimensionally deformed due to sedimentary compression. The left splenial and the left angular represent the only two fully disarticulated bones of the specimen. The medial side of all dermatocranial bones is not accessible because of the encasing matrix, as well as the palatal complex and the neurocranium, with the exception of their respective caudalmost portions.

*Age and stratigraphic horizon*: Late Triassic (latest Norian), uppermost fossiliferous horizon of the fourth subunit of the Gruhalde Member (Klettgau Formation), discordantly overlain almost one meter above by the Early Jurassic (Hettangian) Staffelegg Formation.

*Locality*: clay pit Gruhalde of the Tonwerke Keller AG, Frick, Canton Aargau, Switzerland. Coordinates 47°30′24.0"N 8°00′30.5"E (www.strati.ch).

*Diagnostic craniomandibular features of SMF 13.5.37*: a massopodan sauropodomorph diagnosed by the following autapomorphies (indicated by asterisks) and unique combination of characters, all of which shared in each round of analysis of the data matrix (see Supplementary material, “supporting synapomorphies of Sauropodomorpha nodes present in SMF 13.5.37 and unique combination of characters”): neurovascular foramen at the posterior end of the lateral maxillary row opens ventrally (modified from Sereno, 1999); orientation of the lacrimal orbital margin is erect and close to vertical (Yates, 2007); triangular, caudolateral spur of the nasal that envelops the rostral margins of the lacrimal and the prefrontal within two distinct embayments (*); maximum transverse width of the prefrontal more than 0.25 of the skull width at that level (modified from Galton, 1990); notch on the rostral ramus of the frontal defines a dorsally exposed, S-shaped nasofrontal suture (*); rostrolaterally projecting, triangular process on the lateral surface of the paroccipital processes (*); height: length ratio of the dentary greater than 0.2 (Benton et al., 2000); orientation of the maxillary tooth crowns is procumbent (Gauthier, 1986); orientation of the postorbital ramus of laterosphenoid extends laterally (Chapelle & Choiniere, 2018).

N.B.: the unique combination of cranial features of SMF 13.5.37 changes depending on the round of phylogenetic analysis, thus it accounts for additional characters that are analysis-specific and that are listed and discussed within each respective paragraph (see Supplementary material, “supporting synapomorphies of Sauropodomorpha nodes present in SMF 13.5.37 and unique combination of characters”).

## Morphological description of SMF 13.5.37

### Skull overview and cranial openings

**Fig. 3:**
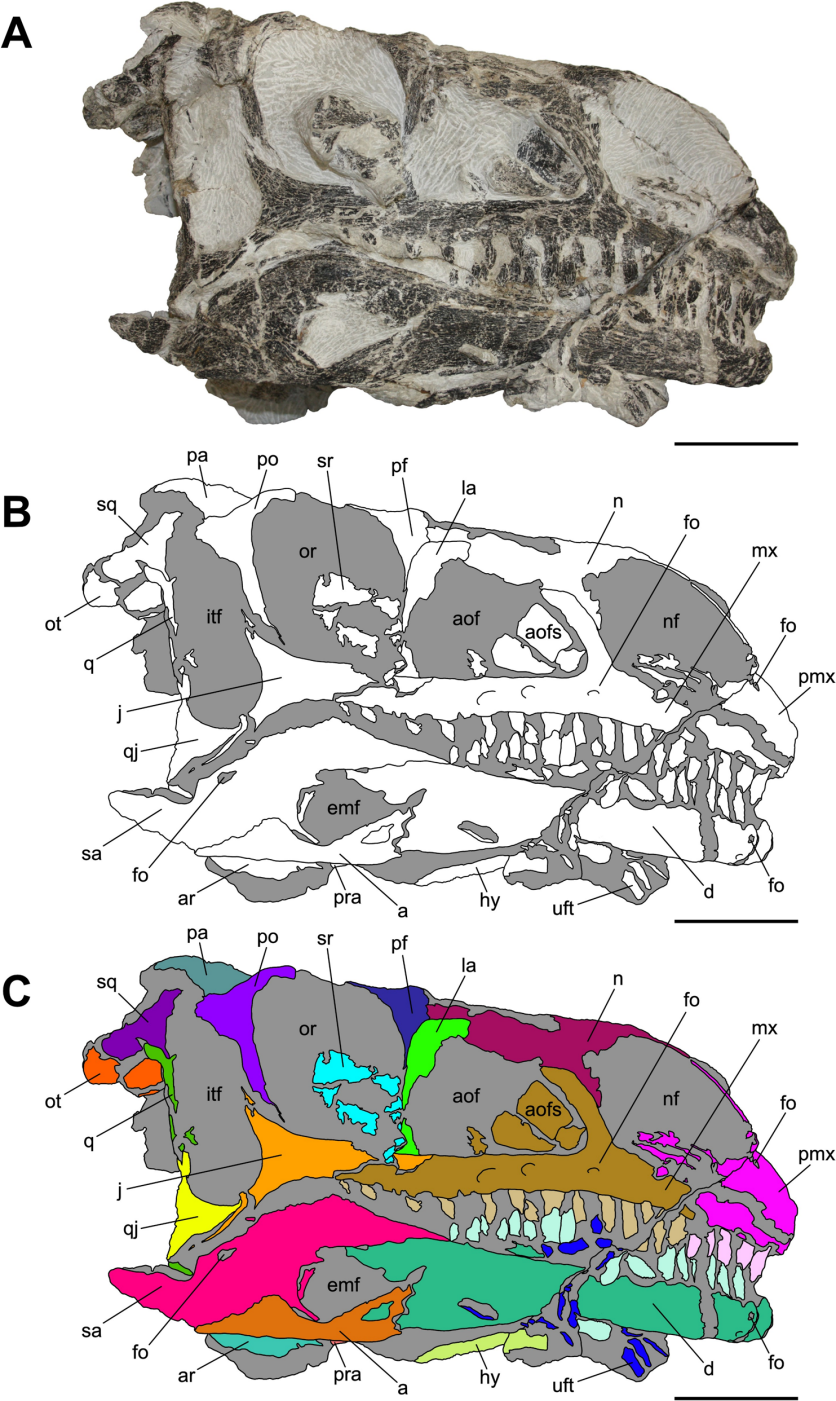
Articulated skull SMF 13.5.37 in right lateral view. **A** Photograph. **B** Interpretative line drawing. White areas correspond to bones, grey surfaces represent matrix. **C** Coloured craniomandibular map. For abbreviations, see “Anatomical abbreviations” section. Scale bars equals 5 cm

**Fig. 4:**
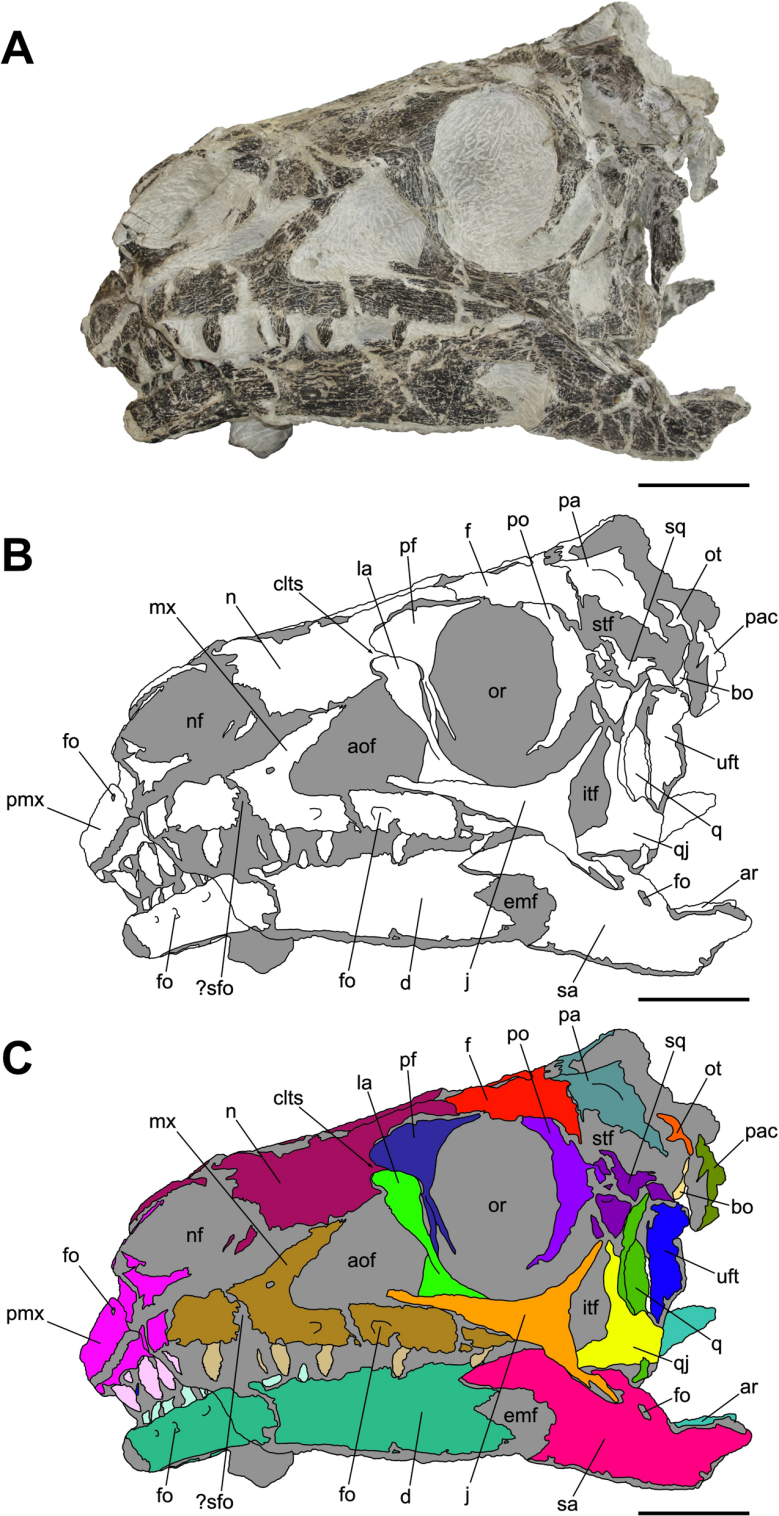
Articulated skull SMF 13.5.37 in left lateral view. **A** Photograph. **B** Interpretative line drawing. White areas correspond to bones, grey surfaces represent matrix. **C** Coloured craniomandibular map. For abbreviations, see “Anatomical abbreviations” section. Scale bars equals 5 cm

**Fig. 5:**
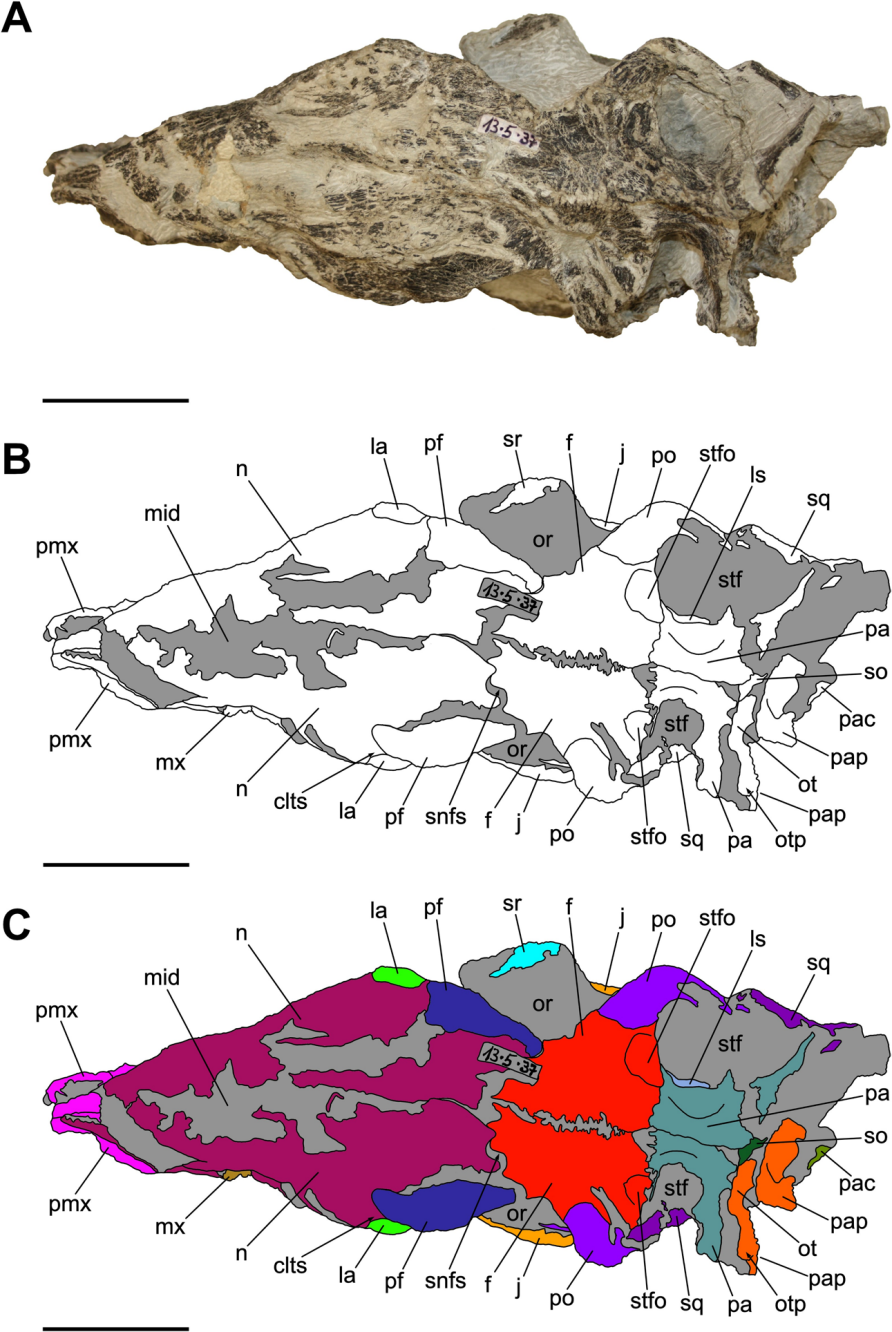
Articulated skull SMF 13.5.37 in dorsal view. **A** Photograph. **B** Interpretative line drawing. White areas correspond to bones, grey surfaces represent matrix. **C** Coloured craniomandibular map. For abbreviations, see “Anatomical abbreviations” section. Scale bars equals 5 cm

**Fig. 6:**
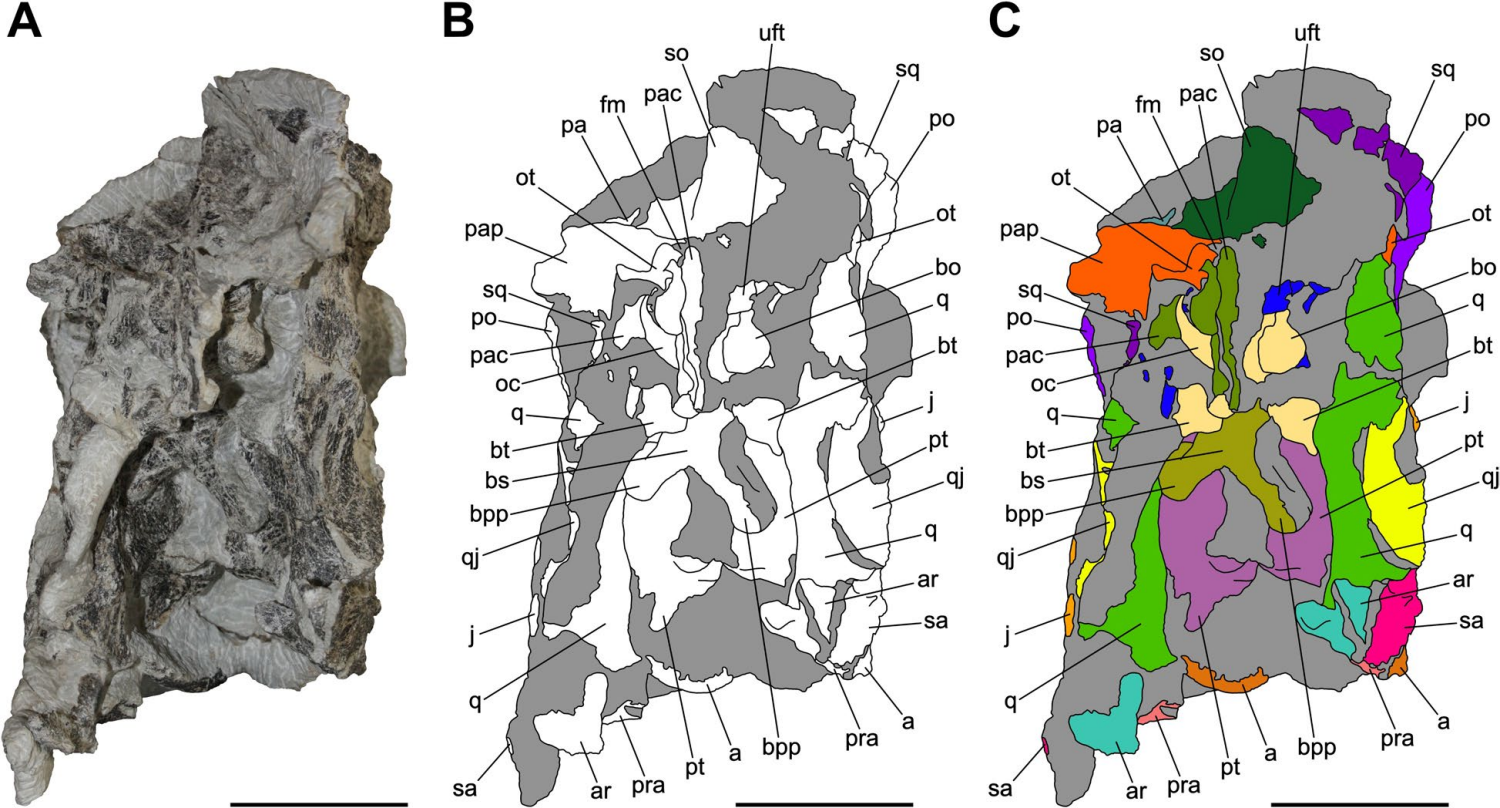
Articulated skull SMF 13.5.37 in caudal view. **A** Photograph. **B** Interpretative line drawing. White areas correspond to bones, grey surfaces represent matrix. **C** Coloured craniomandibular map. For abbreviations, see “Anatomical abbreviations” section. Scale bars equals 5 cm

**Fig. 7:**
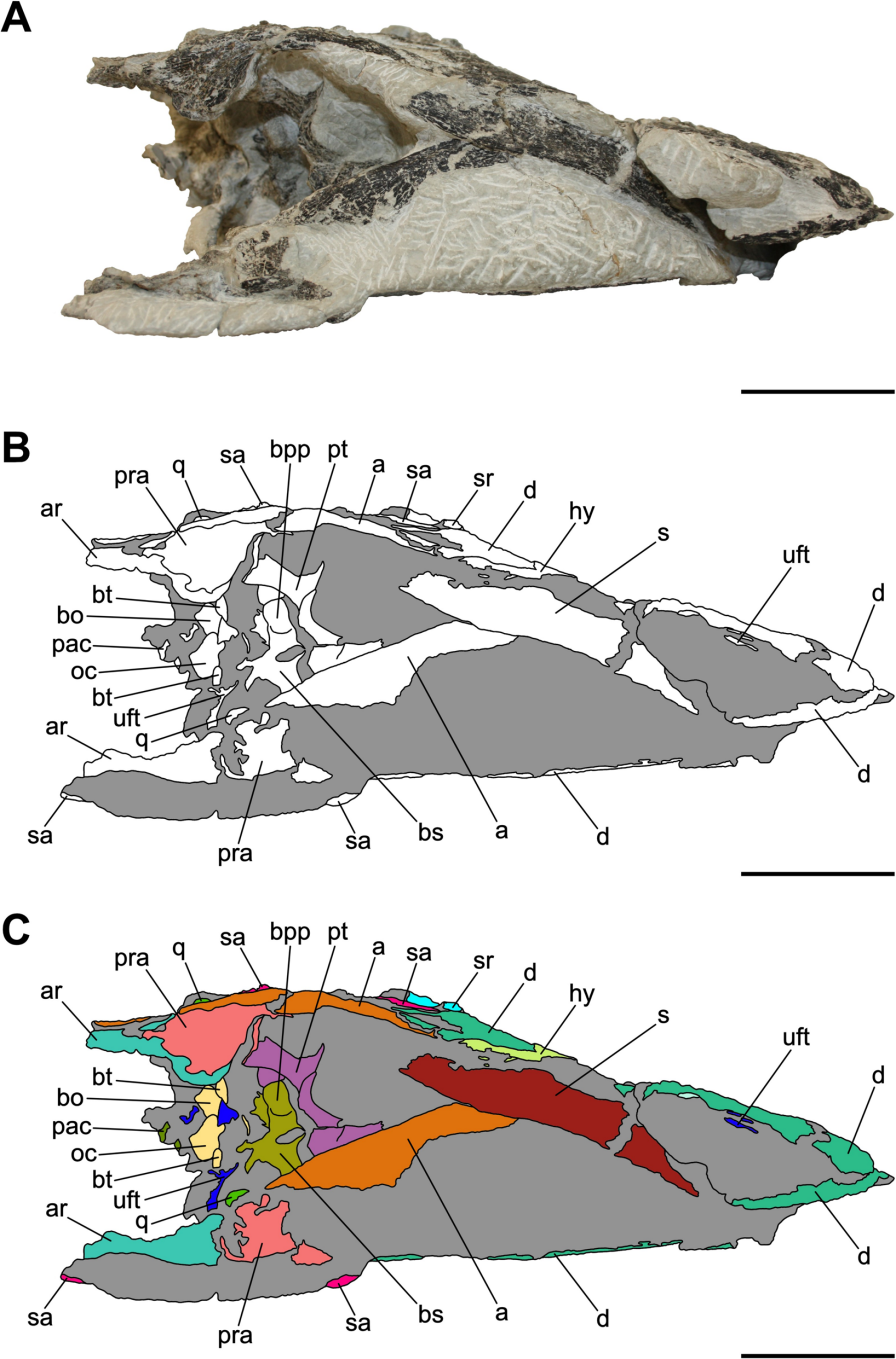
Articulated skull SMF 13.5.37 in ventral view. **A** Photograph. **B** Interpretative line drawing. White areas correspond to bones, grey surfaces represent matrix. **C** Coloured craniomandibular map. For abbreviations, see “Anatomical abbreviations” section. Scale bars equals 5 cm

Characterized by the peculiar black colour of the fossil material recovered from the same horizon (Zahner & Brinkmann, 2019), the specimen SMF 13.5.37 consists of a complete, three-dimensionally preserved skull that is not plastically deformed, differently from several *Pla. trossingensis* skeletons from the lower horizons of the Gruhalde Member (Figs. 3, 4, 5, 6, and 7) (Lallensack et al., 2021). Nonetheless, the left side of the skull is slightly distorted with some cranial bones dislocated from life position, possibly due to sediment compaction during the fossilization process. In detail, the left preorbital region has been dorsoventrally compressed, resulting in the distortion of the external naris and the antorbital fenestra as well as the fragmentation and the rostroventral displacement of both the premaxilla and the nasal (Fig. 4). Additionally, the left caudalmost cranial region is crushed, leading most of the posterior bones to be disarticulated and rostrally shifted and thus affecting the shape of several cranial openings, like the orbit and the temporal fenestrae, which are rostrocaudally compressed (Figs. 4, 6). Overall, a certain degree of general fragmentation occurs, a condition likely derived from swelling and shrinking of the encasing matrix, but also from a long-term exposition of the specimen prior to the complete burial.

The skull is subrectangular in lateral view, being rostrocaudally elongate and dorsoventrally low, whereas it is subtriangular in dorsal view with a mediolaterally tapering snout (Figs. 3, 4, 5). Similarly to several non-sauropodan sauropodomorphs (e.g. Apaldetti et al., 2011, 2014; Barrett et al., 2005; Beccari et al., 2021; Chapelle et al., 2019; Sues et al., 2004; Zhang et al., 2020), the specimen is almost twice as long as high (1.54 left; 1.78 right) and reaches its maximum mediolateral width at the level of the frontal-postorbital contact, defining a length-to-width ratio that spans from 2.67 in the left deformed side to 2.9 in the undistorted right side, being higher than in *Pla. trossingensis* (2.2) (Lallensack et al., 2021), but less than *Yunnanosaurus huangi* Young, 1942 (3.8) (Barrett et al., 2007). Noticeably, the preorbital region is 0.56 times the entire cranial length as in *Mac. itaquii*, displaying an intermediate condition between the longer morphologies of non-massopodan sauropodomorphs, e.g. *Pla. trossingensis*, and the short-snouted condition of massospondylids, e.g. *Mas. carinatus* and *Leyesaurus marayensis* Apaldetti et al., 2011 (Müller, 2020). In caudal view, the skull is taller than wide with a width-to-height ratio between 0.58 (left) and 0.61 (right), differing from the slenderer cranial proportions of *Mas. carinatus* (1.0) and the more robust ones of *Ngwevu intloko* Chapelle et al., 2019 (1.7) (Chapelle et al., 2019).

The external naris is large and subtriangular-shaped with a subhorizontal ventral margin, a vertical posterior margin and a rostroventrally bent dorsal margin. It is bordered by the premaxilla rostrodorsally and rostroventrally, the maxilla caudoventrally and the nasal dorsocaudally (Figs. 3, 4). Noticeably, the maximum rostrocaudal length of the external naris occupies the 26% and the 28% of the entire skull length, respectively in left and right lateral views, and it is proportionately longer than all non-sauropodan sauropodomorphs (Barrett et al., 2007; Chapelle et al., 2019; Zhang et al., 2020). Furthermore, it exceeds half the maximum orbit diameter as in *Mas. carinatus* (Sues et al., 2004).

The antorbital fenestra is trapezoidal and is set within a wide subtriangular antorbital fossa, similarly to non-massopodan sauropodomorphs (Figs. 3, 4), like *Pla. trossingensis* (Lallensack et al., 2021) and *Mac. itaquii* (Müller, 2020), and contrasting the triangular morphology of massopodan sauropodomorphs, as *Mas. carinatus* (e.g. Chapelle & Choiniere, 2018), *Massospondylus kaalae* Barrett, 2009, *Col. brevis* (Apaldetti et al., 2014), *Lufengosaurus huenei* Young, 1941a (Barrett et al., 2005), *Ley. marayensis* (Apaldetti et al., 2011), *Yun. huangi* (Barrett et al., 2007) and *Jingshanosaurus xinwaensis* Zhang & Yang, 1995 (Zhang et al., 2020). In detail, it is bounded by the maxilla rostrally and ventrally, the jugal caudoventrally, the lacrimal caudally and the nasal dorsally.

The right orbit is subcircular, whereas the left one is rostrocaudally compressed due to the displacement of both the jugal and the postorbital (Figs. 3, 4). Nonetheless, their rostrocaudal extent is equivalent to the 20% of the entire skull length, thus being shorter than *Col. brevis* (Apaldetti et al., 2014), *Ley. marayensis* (Apaldetti et al., 2011) and *Yun. huangi* (Barrett et al., 2007), in which it reaches the 30%. The orbit is defined by the prefrontal rostrodorsally, the lacrimal rostrally, the jugal ventrally, the postorbital caudally and the frontal dorsally.

The right supratemporal fenestra is longer than wide and it has a subtrapezoidal outline in dorsal view (Fig. 5). On the other hand, the left one is distorted due to a rostral deformation of the caudolateral process of the parietal which leads the transverse axis of the fenestra to exceed the longitudinal one, resulting in a deformed subrectangular shape (Fig. 5). The supratemporal fenestra is bordered by the frontal rostromedially, the postorbital rostrolaterally, the squamosal caudolaterally and the parietal both caudally and medially.

The right infratemporal fenestra is the only one preserved despite being partially deformed (Fig. 3), whereas the left one is completely collapsed due to taphonomic disarticulation of the caudal cranial bones (Fig. 4). In right lateral view, it is kidney-shaped and constricted at midheight with its rostroventral corner extending beneath the rear edge of the orbit, similarly to several massospondylids (e.g. Apaldetti et al., 2011, 2014; Chapelle & Choiniere, 2018). The infratemporal fenestra is bounded by the postorbital rostrodorsally, the jugal rostroventrally, the quadratojugal caudoventrally and the squamosal dorsocaudally.

The external mandibular fenestra is subtriangular in lateral view (Figs. 3, 4), with its apex pointing rostrally, and it corresponds to the 13% and the 15% of the total rostrocaudal mandibular length of the left and right ramus respectively, thus being shorter than in *Col. brevis* (20%) (Apaldetti et al., 2014). The proportion disparity is due to the disarticulation of the left angular and the rostral displacement of the left surangular (Figs. 4, 7). The external mandibular fenestra is formed by the dentary rostrodorsally, the angular ventrally and the surangular dorsocaudally.

### Premaxilla

**Fig. 8:**
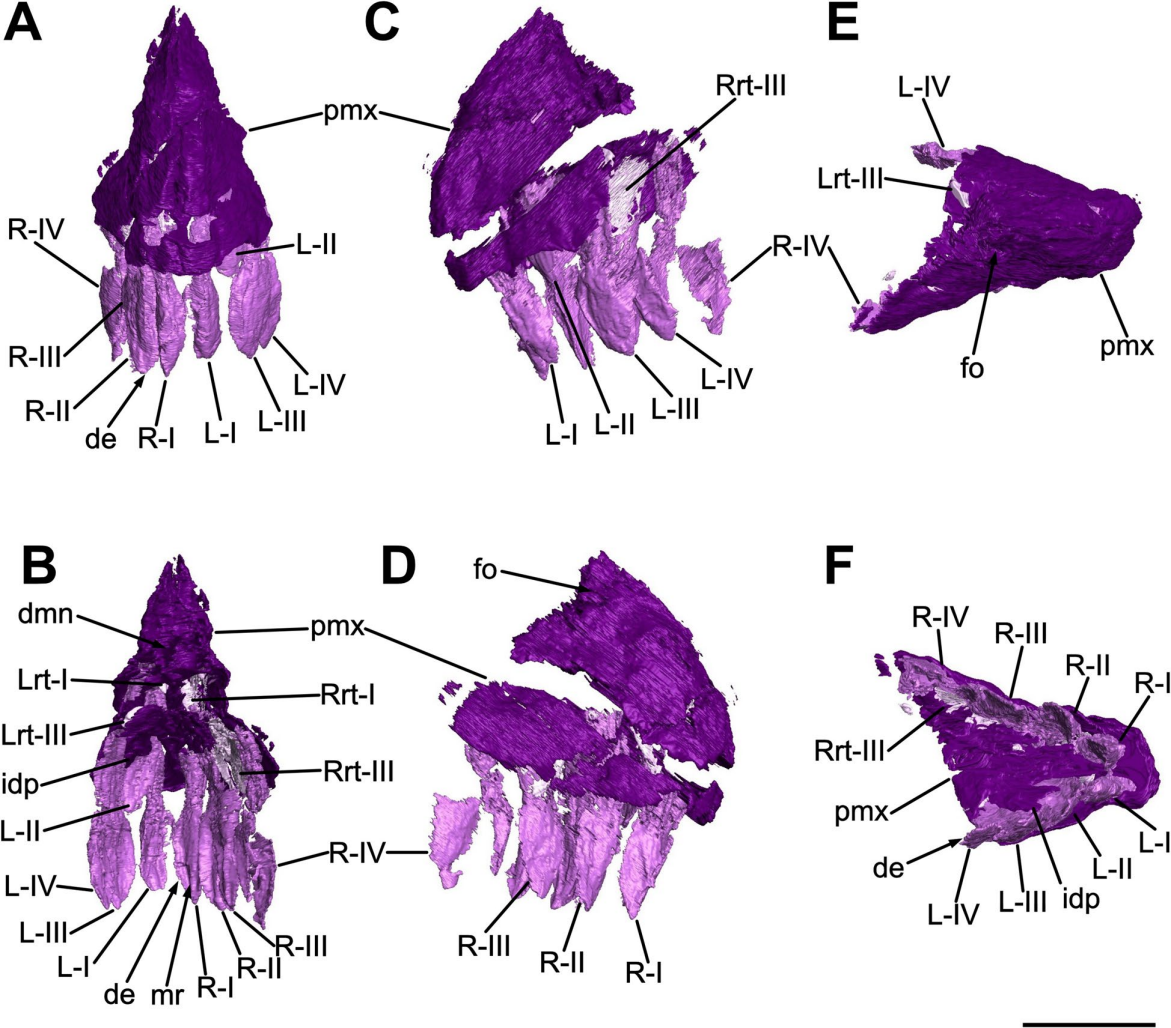
Three-dimensional rendering of the segmented premaxillae and related teeth of SMF 13.5.37. **A** Rostral view. **B** Caudal view. **C** Left lateral view. **D** Right lateral view. **E** Dorsal view. **F** Ventral view. For abbreviations, see “Anatomical abbreviations” section. Scale bar equals 2 cm

The rostralmost portion of the snout is formed by the fused premaxillae, which contribute to both the anterodorsal and the anteroventral margin of the narial fenestrae. Each element has undergone a taphonomic deformation which led the premaxillary bodies to be firstly fragmented and then laterally displaced, resulting in an inclination of approximately 10° degrees to the right of the sagittal plane. Despite not being in proper anatomical positions, it is clear that the premaxillae are complete, mediolaterally compressed and articulated caudolaterally with the maxillae and dorsocaudally with the nasals, respectively forming an inverted L-shaped contact with the former and a point contact with the latter (Figs. 3, 4, 8).

In lateral view, each premaxilla is rostrocaudally longer than dorsoventrally high and possesses a triangular outline defined by a convex premaxillary body from which two rami develop, namely the caudolateral maxillary ramus and the dorsocaudal nasal ramus (Figs. 3, 4). In dorsal and ventral view, the fused premaxillae taper rostrally defining a triangular outline, forming a U-shaped tip of around 40° gap (Fig. 8E, F).

The rostralmost portion of the premaxillary bodies is characterized by a sharp, dorsoventrally straight corner formed by a 90° angle between the ventral and the dorsal margin of each main body (Fig. 8C, D), differing from both the more acute condition of *Mas. carinatus* (Chapelle & Choiniere, 2018) and the more rounded morphology of *Pla. trossingensis* (Lallensack et al., 2021; Schaeffer, 2024). Nonetheless, as in *Mas. carinatus*, *Pla. trossingensis* and most of other non-sauropodan sauropodomorphs, the rostrum tip is more ventrally positioned than the rest of the snout (Chapelle & Choiniere, 2018; Schaeffer, 2024). This condition is determined by an anteroventral sloping of the ventral margin of the premaxillary bodies, which, in SMF 13.5.37, is about 10°–15° with respect to the maxillary alveolar margin (Fig. 3), differing from *Pla. trossingensis* (20°) (Schaeffer, 2024) and *Mas. carinatus* (35°) (Chapelle & Choiniere, 2018).

The rostrodorsal margin of the premaxillary body is convex and slopes rostroventrally forming an angle of 45° with the alveolar margin, sharing a low-snouted morphology with *Pla. trossingensis* (Prieto-Márquez & Norell, 2011; Schaeffer, 2024), *Col. brevis* (Apaldetti et al., 2014) and *Yun. huangi* (Barrett et al., 2007), but contrasting the short-snouted non-massopodan plateosaurians, like *Iss. saaneq* (Beccari et al., 2021), *Unaysaurus tolentinoi* Leal et al., 2004, (McPhee et al., 2019), and the massospondylids *Mas. carinatus* and *Ngw. intloko* (Chapelle & Choiniere, 2018; Chapelle et al., 2019).

Both maxillary rami are not well-preserved, showing certain degrees of breakage and disarticulation from the premaxillary bodies. Specifically, the right one is fragmented and displaced further back than its original position (Fig. 3), whereas the left one is only disarticulated from the premaxilla but maintaining its three-dimensional shape (Fig. 4). Generally, the maxillary ramus is subequal to the length of the premaxillary body and develops caudomedially as a concave, tapering bony wall that overlies the premaxillary ramus of the maxilla for most of its length, but neither reaching the caudoventral corner of the external naris nor the rostroventral process of the nasal. The concavity can be referred as a deep, D-shaped recess that contributes to both the rostralmost portion of the narial fossa and to the rostroventral rim of the external naris. A similar condition of the narial fossa is present in *Pla. trossingensis* (Prieto-Márquez & Norell, 2011; Schaeffer, 2024), being marked by a ventral rim and a marked concavity, but not in other plateosaurian sauropodomorphs possessing a weaker depression, like *Iss. saaneq* (Beccari et al., 2021), *U. tolentinoi* (McPhee et al., 2019), *Mac. itaquii* (Müller, 2020) and *Mas. carinatus* (Chapelle & Choiniere, 2018).

In caudal view, the premaxilla expands medioventrally above the fourth alveolus as a short, mediolaterally thin process that forms the rostralmost portion of the palate, as detected in the 3D rendering of the bone itself (Fig. 8B). Furthermore, it outlines a dorsomedial notch where the rostromedial process of the maxilla likely articulated (Fig. 8B), similarly to *Iss. saaneq* (Beccari et al., 2021), *U. tolentinoi* (McPhee et al., 2019) and *Mas. carinatus* (Chapelle & Choiniere, 2018).

The nasal rami are well preserved, despite being separated from their broad, proximal base because of the displacement of the premaxillary bodies. Defining both the rostrodorsal margin and the rounded rostroventral corner of the external naris, the nasal ramus of each premaxilla develops dorsocaudally from the rostral margin of the premaxillary body at the level of the fourth alveolus with an inclination of about ~35° to the premaxillary alveolar margin (Figs. 3, 4), similarly to *Pla. trossingensis* (35°) (Prieto-Márquez & Norell, 2011), *U. tolentinoi* (40°) (McPhee et al., 2019), *Mac. itaquii* (45°) (Müller, 2020) and *Yun. huangi* (Barrett et al., 2007), but contrasting most of the massospondylids (e.g. Chapelle & Choiniere, 2018; Chapelle et al., 2019). Characterized by a gradual dorsoventral reduction from the proximal base, the nasal ramus gradually expands mediolaterally while extending caudally, almost reaching the level of the maxillary ramus, and defining its widest point at the distalmost end where it contacts the nasal (Fig. 5), similarly to *Iss. saaneq* (Beccari et al., 2021), *Mac. itaquii* (Müller, 2020), *Pla. trossingensis* (Prieto-Márquez & Norell, 2011; Schaeffer, 2024) and *Col. brevis* (Apaldetti et al., 2014).

Neither neurovascular foramina nor subnarial foramina can be clearly identified on the lateral surfaces, even though a possible analogue of the latter, being large and oval in shape, is found at each caudal contact between the premaxillary bodies and the rostral processes of the maxillae (Fig. 4). Nonetheless, a pair of rostroventrally opening, elliptical foramina is also present at the base of each nasal ramus, at the level of the third premaxillary tooth (Figs. 3, 4, and 8D).

Each premaxilla bears four premaxillary teeth as in most plateosaurian sauropodomorphs, like the unaysaurids *U. tolentinoi* (McPhee et al., 2019) and *Mac. itaquii* (Müller, 2020), and in massospondylids as well, like *Sarahsaurus aurifontanalis* Rowe et al., 2011, *Mas. carinatus* (Chapelle & Choiniere, 2018), *Ngw. intloko* (Chapelle et al., 2019)*, Ley. marayensis* (Apaldetti et al., 2011), *Adeopapposaurus mognai* Martínez, 2009, but also as in several non-sauropodan sauropodiforms, like *Jin. xinwaensis* (Zhang et al., 2020), *Qianlong shouhu* Han et al., 2023, *Anchisaurus polyzelus* Hitchcock, 1865 (Yates, 2010) and *Melanorosaurus readi* Haughton, 1924 (Yates, 2007). Contrasting the condition of SMF 13.5.37, five or more premaxillary teeth are present in *Iss. saaneq* (Beccari et al., 2021), *Pla. trossingensis* (Lallensack et al., 2021; Prieto-Márquez & Norell, 2011; Schaeffer, 2024), *Rio. incertus* (Bonaparte & Pumares, 1995) and *Aardonyx celestae* Yates et al., 2010 (Yates et al., 2010).

The alveolar margin, which accounts for 18% of the complete upper tooth row length, is continuous, straight and inclined at about 10°–15° with respect to the maxillary alveolar margin of the jugal ramus, differently from *Pla. trossingensis* (20°) (Schaeffer, 2024), and it does not present any diastema between the last premaxillary tooth and the first maxillary tooth (Figs. 3, 4). Furthermore, the labial alveolar margin is deeper than the lingual one, leading to a partial medial exposure of the tooth roots (Fig. 8B). A putative triangular interdental plate is found in between the third and fourth tooth positions in the right premaxillary tooth row, visible in the 3D model of the premaxilla (Fig. 8F).

Noticeably, a gap is present between the tip of the snout and the first premaxillary alveolus (Fig. 8C, D, F), similarly to somatically mature individuals of *Pla. trossingensis* (Lallensack et al., 2021) and *Ley. marayensis* (Apaldetti et al., 2011).

### Maxilla

Both elements are slightly deformed, with the right one being gently bent inwards at its midlength, which partially accentuates the concave outline of its alveolar margin, whereas the left one is both fragmented along its caudal process and dorsoventrally shorter because of a downward pressed dorsal process.

Defining the caudoventral and caudal margin of the narial fenestra as well as the ventral and rostral outline of the antorbital fenestra, each maxilla possesses a triradiate lateral profile, being rostrocaudally longer than dorsoventrally high, which contacts the premaxilla rostrally, the nasal dorsally and the jugal caudally (Figs. 3, 4). The medial side of both maxillae is obscured and thus the articulation with the palatal complex cannot be determined.

In lateral view, the premaxillary ramus possesses a convex, subtrapezoidal shape which is rostrocaudally longer than dorsoventrally high, with a length: height ratio between 1.5 (left) and 1.7 (right), similarly to most basal sauropodomorphs, massopodans and basal sauropodiforms, but not to *U. tolentinoi* (McPhee et al., 2019), *Mas. kaalae* (Barrett, 2009), *Ley. marayensis* (Apaldetti et al., 2011), *Ade. mognai* (Martínez, 2009), *Yun. huangi* (Barrett et al., 2007) and *Mel. readi* (Yates, 2007). The rostral margin is rostroventrally-dorsocaudally inclined by almost 70° and perfectly matches the caudal edge of the premaxillary body, where an inverted L-shaped articulation between premaxilla and maxilla is established (Figs. 3, 4). In lateral view, as in the majority of non-sauropodan sauropodomorphs, the dorsal and ventral margins of each rostral process are subparallel, expanding dorsoventrally while extending caudally towards the nasal ramus. Furthermore, the right premaxillary ramus is rostroventrally oriented by 10°-15° with respect to the maxillary tooth row of the jugal ramus (Fig. 3), similarly to the premaxillary bodies, whereas the left element has been slightly upturned, resulting in a horizontal inclination, due to a taphonomic breakage at the base of the process subsequently followed by a dorsolateral displacement (Fig. 4). The dorsal half of the rostral process curves dorsomedially forming a medial shelf, laterally marked by a shallow groove that fades caudally at the level of the third maxillary tooth, where the articular facet for the maxillary ramus of the premaxilla is positioned. Posteriorly, the medial shelf slopes dorsocaudally, defining the caudoventral corner of the narial fenestra, and fades into the rostral margin of the nasal ramus. On the other hand, both the ventral and the alveolar margins of each premaxillary ramus are inclined accordingly to the orientation of their respective process.

The nasal ramus develops dorsocaudally from the conjunction point of the three maxillary rami, which is positioned prior to the midlength of the maxilla, specifically within the rostralmost one-third of the bone extent (Figs. 3, 4), similarly to most plateosaurians, like *Pla. trossingensis* (Prieto-Márquez & Norell, 2011; Schaeffer, 2024), *Mas. carinatus* (Chapelle & Choiniere, 2018) and *Luf. huenei* (Barrett et al., 2005), but not to *U. tolentinoi* (McPhee et al., 2019). It ascends as a laterally convex, elongated process with subparallel margins that tapers distally. Despite the rostrocaudal width of the nasal ramus does not significantly vary along almost its entire height, a slight expansion is present on the dorsal margin at the base of the caudal margin of the maxillary ramus of the nasal.

The rostral margin of the nasal ramus is convex and slopes medially, being continuous with the dorsal margin of the premaxillary ramus and defining the caudoventral margin of the narial fenestra and partially of the narial fossa. Furthermore, the rostrodorsal half of the dorsal process is overlapped by the maxillary ramus of the nasal which extensively extends caudally covering also the dorsal margin of the nasal ramus of the maxilla, thus obscuring a possible contact between the maxilla and the lacrimal (Figs. 3, 4). On the other hand, the caudal margin of the nasal ramus is concave and sharp, and it bounds both the rostral margin and the rostroventral corner of the antorbital fossa and the antorbital fenestra as well.

The right nasal ramus smoothly curves dorsocaudally with an inclination at the base of 60° and then, distally, of 55° relative to the maxillary alveolar margin of the jugal ramus (Fig. 3). On the other hand, the left nasal ramus is characterised by more acute angles, respectively 50° proximally and 30° distally, due to taphonomic deformation that led to a dorsoventral distortion of the process itself (Fig. 4). A comparable orientation of the nasal ramus is recorded in several non-sauropodan sauropodomorphs, e.g. *Pla. trossingensis* (Chapelle & Choiniere, 2018; Lallensack et al., 2021) and *Yun. huangi* (Barrett et al., 2007), but not in *U. tolentinoi* (McPhee et al., 2019) and in massospondylids, like *Col. brevis* (Apaldetti et al., 2014), *Mas. carinatus* and *Ngw. intloko* (Chapelle et al., 2019), with the exceptions of *Luf. huenei* (Chapelle & Choiniere, 2018) , *Mas. kaalae* (Barrett, 2009) and *Ley. marayensis* (Apaldetti et al., 2011). The gradual deflection occurs at the midheight of the antorbital fenestra, which coincides with the ventralmost portion of the wide contact between the nasal ramus of the maxilla and the maxillary ramus of the nasal, similarly to *Pla. trossingensis*, *Rio. incertus*, *Col. brevis* and *Luf. huenei*, but contrasting the condition of some basal sauropodiforms (Apaldetti et al., 2011).

Differently from the massospondylids *Mas. carinatus* (Chapelle & Choiniere, 2018) and *Ley. marayensis* (Apaldetti et al., 2011), no distinct ridges are present on the lateral surface of the nasal ramus. Nonetheless, one neurovascular foramen is found close to the base of the left dorsal process (Fig. 4).

Extending behind the right nasal ramus, a medially recessed bony lamina defines the rostral portion of the antorbital fossa within the front half of the antorbital fenestra (Fig. 3). The exact shape and size of the maxillary contribution to the antorbital fossa cannot be fully established because of the presence of encasing matrix on its caudal margin. Furthermore, the maxillary contribution to the left antorbital fossa is not present, either because of taphonomy or lack of preparation. Despite being fragmented into four pieces, three of which are slightly displaced, the antorbital fossa is well-developed and subtriangular in shape without presenting any promaxillary fenestra (Fig. 3), differently from the non-massopodan plateosaurians *U. tolentinoi* (McPhee et al., 2019), *Mac. itaquii* (Müller, 2020) and possibly *Iss. saaneq* (Beccari et al., 2021). Furthermore, unlikely *Pla. trossingensis* (Prieto-Márquez & Norell, 2011) but similarly to most of the basal sauropodomorphs, the rostrocaudal extent is shorter than the orbit diameter. The rostral margin is marked by the sharp, raised above caudal margin of the nasal ramus, whereas the caudal margin appears to be straight, developing from the midlength of the jugal ramus and terminating almost perpendicularly at the distalmost end of the nasal ramus, like in *U. tolentinoi* (McPhee et al., 2019) and *Mel. readi* (Yates, 2007). Despite being mostly crescent-shaped in other taxa, a similarly wide maxillary contribution to the antorbital fossa is shared with non-massopodan sauropodomorphs, like plateosaurids, e.g. *Pla. trossingensis* (Lallensack et al., 2021; Prieto-Márquez & Norell, 2011; Schaeffer, 2024), unaysaurids, e.g. *U. tolentinoi* (McPhee et al., 2019) and *Mac. itaquii* (Müller, 2020), and non-sauropodan sauropodiforms, e.g. *Aar. celestae* (Yates et al., 2010) and *Mel. readi* (Yates, 2007). Contrastingly, most massospondylids have a caudally concave and rostrocaudally reduced antorbital fossa, like *Mas. carinatus* (Chapelle & Choiniere, 2018), *Ngw. intloko* (Chapelle et al., 2019), *Ley. marayensis* (Apaldetti et al., 2011) and *Ade. mognai* (Martínez, 2009), with the sole exception of *Col. brevis*, rather resembling the condition of non-massospondylid sauropodomorphs (Apaldetti et al., 2014) and SMF 13.5.37.

The jugal ramus extends caudally to the nasal ramus, forming an elongate, distally tapering process with subparallel margins, as in early sauropodomorphs (Figs. 3, 4), that accounts for at least 65% of the maxillary length, being comparable to *Mas. carinatus* (60%) (Chapelle & Choiniere, 2018). Defining the ventral margin of both the antorbital fossa and antorbital fenestra, the dorsal margin is firstly horizontal, with a weak dorsolateral expansion just prior to the contact between maxilla and jugal, and then obliquely sloping caudoventrally along its distalmost portion, marking a dorsocaudal articulation surface for the latter bone. Similarly, the ventral margin, which defines most of the maxillary alveolar margin, is straight along its anterior half, subsequently it inflates ventrally at the same level of the dorsolateral expansion and distally it converges dorsocaudally with the dorsal margin. Remarkably, the dorsoventral deepest point of the entire process is found right before the maxilla-jugal suture, alike *Pla. trossingensis* (Prieto-Márquez & Norell, 2011; Schaeffer, 2024). The lateral surface of the jugal ramus is concave and possesses a groove of linearly arranged neurovascular foramina (Figs. 3, 4), similarly to *U. tolentinoi* (McPhee et al., 2019) and *Mac. itaquii* (Müller, 2020), that extends from the base of the nasal process until the dorsoventral expansion of the jugal ramus. Remarkably, SMF 13.5.37 possess one of the lowest counts of maxillary neurovascular foramina among early sauropodomorphs, specifically accounting for three pits per ramus. Generally, the neurovascular foramina open rostroventrally, with the exception of the distalmost one, which is ventrally directed and, as in most basal sauropodomorphs, is the largest in size (Figs. 3, 4) (e.g. Apaldetti et al., 2011; Beccari et al., 2021; Chapelle & Choiniere, 2018; Lallensack et al., 2021).

Both maxillary tooth rows are ventrally obscured by the encasing matrix, thus hindering the maxillary tooth count. Nonetheless, taking into account both the tooth size and the interalveolar space, each maxilla appears to bear around 21 alveoli, 12 of which are still occupied by teeth in the right element (Fig. 3), whereas the left one preserves only 5 (Fig. 4). Remarkably, 4 tooth positions are present within the premaxillary ramus, accounting for the 24% and the 26% of the left and the right maxillary tooth row respectively. A similar condition, regarding both the maxillary tooth count and the alveolar distribution within the maxillary tooth row, is shared with subadult individuals of *Pla. trossingensis* (22) (Lallensack et al., 2021), unaysaurids, like *U. tolentinoi* (>19) (Leal et al., 2004; McPhee et al., 2019) and *Mac. itaquii* (22) (Müller, 2020), and with some massospondylids, specifically *Luf. huenei* (20) (Barrett et al., 2005), *Col. brevis* (22) (Apaldetti et al., 2014) and fully grown specimens of *Mas. carinatus* (14–22) (Chapelle et al., 2019). On the other hand, the plateosaurids *Iss. saaneq* and *Pla. trossingensis*, especially somatically mature individuals, possess both a higher number of alveoli within the maxilla, 23–24 for the former (Beccari et al., 2021) and 23–30 for the latter (Lallensack et al., 2021; Prieto-Márquez & Norell, 2011; Schaeffer, 2024), and 5 teeth positions within the premaxillary ramus.

In lateral view, being consistent in morphology with the ventral margin of each maxilla, the left maxillary alveolar margin is straight-to-convex (Fig. 4), whereas the right one is concave-to-convex (Fig. 3), similarly to non-massopodan sauropodomorphs, e.g. *Pla. trossingensis* (Lallensack et al., 2021; Prieto-Márquez & Norell, 2011) and *Mac. itaquii* (Müller, 2020). In addition, the right maxillary tooth row extends beneath the first one-third of the orbit diameter (Fig. 3), alike *Pla. trossingensis* (Lallensack et al., 2021), *Mac. itaquii* (Müller, 2020) and *Mas. carinatus* (Chapelle & Choiniere, 2018) but not reaching its midlength as in *Col. brevis* (Apaldetti et al., 2014). Discordantly, the left maxillary tooth row extends further back because of taphonomic deformation of the orbit (Fig. 4).

### Nasal

The nasals are not well-preserved, being both fragmented and partially crushed on their dorsal surface. In contrast to the right element, which retains most of its three-dimensional shape (Figs. 3, 5), the left element is downward displaced at the level of the nasal process of the left maxilla due to taphonomic distortion (Figs. 4, 5).

In lateral view, the nasal possesses a tetraradiate profile with subhorizontal dorsal margin, outlining the dorsal rim of both the external naris and the antorbital fenestra, and articulates medially with its counterpart, rostrally with the premaxilla, rostroventrally with the maxilla, dorsolaterally with the lacrimal and the prefrontal as well, and caudally with the frontal (Figs. 3, 4). Furthermore, an oval shaped internasal depression runs longitudinally along the medial articulation between the nasals, restricting the contact to their rostralmost and caudalmost ends (Fig. 5), as in several sauropodomorphs like *Pla. trossingensis*, *Mac. itaquii*, *Mas. carinatus*, *Ngw. intloko*, *Luf. huenei, Ade. mognai* and *Mel. readi* (Chapelle et al., 2019; Lallensack et al., 2021; Müller, 2020; Schaeffer, 2024). Nonetheless, it is not clear whether this feature might be considered a fenestra rather than a depression because of scarce preservation.

Dorsally, both elements consist of a longitudinally-stretched, dorsolaterally convex triangular main body, being rostrocaudally longer than wide, that defines the rostral-to-central portion of the skull roof as in most basal sauropodomorphs, e.g. *Iss. saaneq* (Beccari et al., 2021), *Mac. itaquii* (Müller, 2020), *Col. brevis* (Apaldetti et al., 2014) and *Mas. carinatus* (Chapelle & Choiniere, 2018). Remarkably, the nasals extend for more than half of the rostrocaudal length of the entire skull, specifically for the 57%. A comparable condition is present only in *Pla. trossingensis* (Galton & Upchurch, 2004; Lallensack et al., 2021; Prieto-Márquez & Norell, 2011; Schaeffer, 2024).

From the main body of the nasal three distinct processes develop, namely the rostrodorsal premaxillary ramus, the rostroventral maxillary ramus and the caudal frontal ramus. The right nasal is used as reference for the former two processes, whereas the left one for the latter.

The premaxillary ramus is a dorsoventrally thin, splint-like process that mediolaterally tapers towards the point of contact with the premaxillary nasal process, whereas proximally it broadens both transversally and dorsoventrally (Fig. 3). Defining the dorsal margin and part of the dorsocaudal corner of the narial fenestra, it curves rostroventrally with an angle of 30° with respect to the premaxillary alveolar margin, being more horizontally oriented than non-massopodan sauropodomorphs, e.g. *Pla. trossingensis* (Lallensack et al., 2021; Prieto-Márquez & Norell, 2011) and *Mac. itaquii* (Müller, 2020), but not as the condition in the massospondylid *Mas. carinatus* (Chapelle & Choiniere, 2018). However, the rostrocaudal length of the premaxillary ramus is comparable to non-massopodan sauropodomorphs, like plateosaurids (e.g. Beccari et al., 2021; Prieto-Márquez & Norell, 2011).

The maxillary ramus of the nasal is a proximally broad, triangular shaped bony lamina that projects rostroventrally (Fig. 3). Its dorsal margin is continuous with the ventral margin of the premaxillary ramus, encompassing part of the dorsocaudal corner and the dorsal half of the caudal margin of the narial fenestra, whereas the ventral margin overlaps the dorsal process of the maxilla defining a curving maxilla-nasal suture.

Possessing a concave lateral surface, the rostroventral process of the nasal sharply tapers onto the maxillary nasal ramus, extending until the midheight of the latter, where a shift in the inclination occurs (see “Maxilla” section), and without reaching either the caudoventral corner of the narial fenestra or the caudolateral process of the premaxilla (Fig. 3). A relatively short maxillary ramus of the nasal that fails to contact the premaxilla is present in most basal sauropodomorphs (Galton & Upchurch, 2004; Yates, 2003a), except for *Efr. minor* and *Pla. trossingensis* (Lallensack et al., 2021; Schaeffer, 2024). The premaxillary ramus and the maxillary ramus diverge from each other with an angle of 55°, differently from *U. tolentinoi* (70°) (McPhee et al., 2019).

In lateral view, behind the broad base of the rostrodorsal process, a weak depression separates the anterior rami from the main body of the nasal, which in turn defines a swollen, convex apex dorsally to the rostroventral process (Figs. 3, 4), similarly to *Pla. trossingensis* (Lallensack et al., 2021; Prieto-Márquez & Norell, 2011), *Mas. carinatus* (Chapelle & Choiniere, 2018) and *Col. brevis* (Apaldetti et al., 2014), but not as pronounced as in *Ngw. intloko* (Chapelle et al., 2019) and *Luf. huenei* (Barrett et al., 2005). Caudally to the dorsal apex, the main body of the nasal flares dorsolaterally forming a subtriangular shelf that reaches the maximum mediolateral width at the rostral contact with the lacrimal, being twice as wide at the base of the premaxillary ramus (Figs. 3, 4), similarly to *Pla. trossingensis* (Schaeffer, 2024). Despite most of the nasals and the left lateral shelf as well are fragmented and crushed, it is likely that the dorsal surface was convex, with the exception of the median internasal depression.

In lateral view, the lateral flange extends rostrocaudally from the distal end of the nasal ramus of the maxilla to the rostral process of the lacrimal, defining an extensive, slightly ventrolaterally inclined overhang that marks the entire dorsal rim of the antorbital fenestra and that covers a possible contact between the maxilla and the lacrimal. Discordantly to massospondylids, a similar morphology is retained in non-massopodan sauropodomorphs, like plateosaurids and unaysaurids, despite in *Mac. itaquii* the lateral shelf does not cover the maxilla-lacrimal suture (Beccari et al., 2021; Bonaparte & Pumares, 1995; Lallensack et al., 2021; Müller, 2020). Clearly visible on the left element, the lateral surface of the flange protrudes caudally beneath the rostral ramus of the lacrimal as a pointing, hook-like prong (Fig. 4), generally referred as caudolateral process, contributing to the nasal-lacrimal contact, as in several basal sauropodomorphs (Langer & Benton, 2006), like *Eor. lunensis* (Sereno et al., 2013), *Bur. schultzi* (Müller et al., 2018a), *Pla. trossingensis* (Lallensack et al., 2021; Schaeffer, 2024), *Mac. itaquii* (Müller, 2020) and *Mel. readi* (Yates, 2007), but also theropods (Langer & Benton, 2006).

**Fig. 9:**
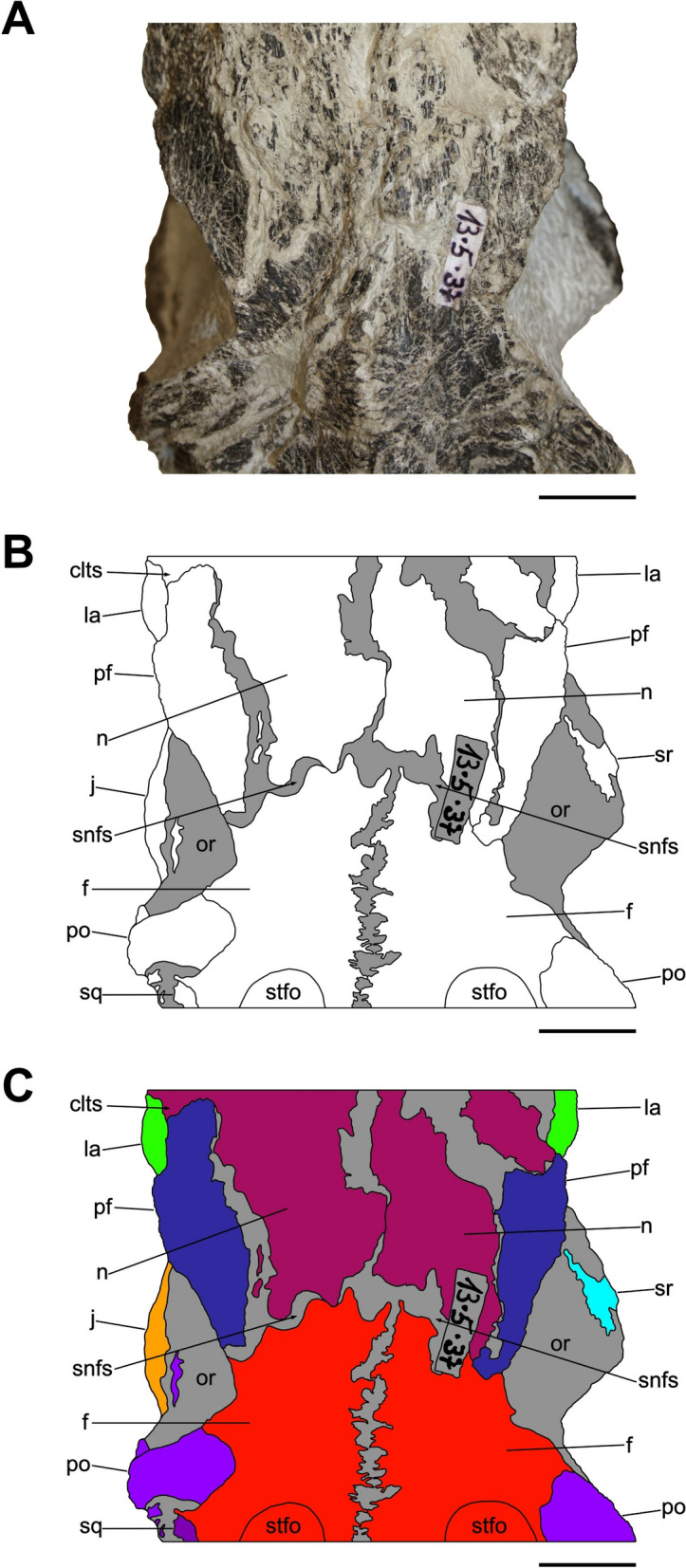
Close-up of the nasal and frontal autapomorphies of SMF 13.5.37 in dorsal view. **A** Photograph. **B** Interpretative line drawing. White areas correspond to bones, grey surfaces represent matrix. **C** Coloured craniomandibular map. For abbreviations, see “Anatomical abbreviations” section. Scale bars equals 2 cm

On the left nasal, at the same level of the hook-like process but dorsally, a second caudolateral projection extends caudally between the lacrimal and the prefrontal as a triangular spur that envelops the rostral margins of the latter bones within two distinct embayments, the lateralmost of which is continuous with the dorsal margin of the caudolateral prong (Figs. 3, 5, and 9). Among non-sauropodan sauropodomorphs, a similar process is shared only with *Arcusaurus pereirabdalorum* Yates et al., 2011, despite being larger in size and ornamented. Taking into account the stratigraphic gap and the different phylogenetic position, this additional caudolateral process of the nasal is herein considered a potential autapomorphy of SMF 13.5.37 convergent in *Arc. pereirabdalorum* (Yates et al., 2011).

The nasal extensively continues caudomedially forming the subrectangular frontal ramus, which laterally contacts the prefrontal and caudally the frontal (Figs. 5 and 9). The slightly concave nasal-prefrontal contact extends along the 87% of the medial margin of the prefrontal, greater than in *Pla. trossingensis* (70%) (Lallensack et al., 2021).

The nasal possesses a caudal peg-like process, visible only in the left element, that slots in between the prefrontal and the frontal, defining an S-shaped nasal-frontal suture that is shallowly depressed in regard to the main dorsal surface of the nasal itself (Figs. 5 and 9). On the other hand, the caudomedial portion of this contact fades medially because of scarce preservation, even though it was likely to be either mediolaterally straight or slightly convex, based on the rostrodorsal margin of the nasal process of the right frontal. Nonetheless, SMF 13.5.37 remarkably differs from both the straight and V-shaped nasal-frontal contacts of basal sauropodomorphs (e.g. Lallensack et al., 2021; Martínez & Alcober, 2009; Müller, 2020; Sereno et al., 2013) and from the oblique, more medially placed suture of some massospondylids and basal sauropodiforms as well (e.g. Apaldetti et al., 2014; Rowe et al., 2011).

An analogous caudal process is present in *Mac. itaquii*, *Sar. aurifontanalis*, *Ley. marayensis*, *Luf. huenei* and *Ade. mognai* but it is different in morphology, having a triangular outline that is remarkably larger in size in all cited taxa. In addition, they differ from the condition of SMF 13.5.37 due to a rostromedially-caudolaterally oblique suture that is defined by an overlapping of the nasal on the frontal, rather than an interlocking, S-shaped contact (Apaldetti et al., 2011; Barrett et al., 2005; Martínez, 2009; Müller, 2020; Rowe et al., 2011). Furthermore, some specimens of *Pla. trossingensis* (e.g. SMF 12.3, SMF 15.4) show an apparent comparable condition, but it is because of taphonomic distortion that led the nasals to be slightly displaced caudally and overlapping the frontal (Lallensack et al., 2021).

### Lacrimal

Both lacrimals are well-preserved, despite some minor degrees of fragmentation and deformation, respectively related to the dorsal half of the right element (Fig. 3) and the main shaft of the left one (Fig. 4). Furthermore, the ventral ramus of the right lacrimal is partially hidden by the rostroventrally displaced sclerotic ring.

In lateral view, bounding the dorsocaudal margin of the antorbital fenestra and the rostroventral margin of the orbit, the lacrimal possesses an inverted L-shaped outline accounting for two perpendicularly merging processes, specifically a rostral ramus that is overlapped rostrodorsally by the nasal and caudomedially by the prefrontal and a caudoventral ramus distally contacting the jugal (Figs. 3, 4). As in several basal sauropodomorphs (e.g. Apaldetti et al., 2014; Lallensack et al., 2021; Pretto et al., 2019; Zhang et al., 2020), the right lacrimal is rostroventrally inclined by 70° with respect to the dorsal margin of the jugal ramus of the maxilla (Fig. 3), whereas the right bone shows a more acute angle, 65° (Fig. 4), due to taphonomic deformation. Nonetheless, it differs from the lowered angled condition present in some specimens of *Pla. trossingensis* (Lallensack et al., 2021; Schaeffer, 2024), *Iss. saaneq* (Beccari et al., 2021), *Mas. carinatus* (Chapelle & Choiniere, 2018), *Mas. kaalae* (Barrett, 2009), *Luf. huenei* (Barrett et al., 2005), as well as from the more perpendicular morphology of *Mac. itaquii* (Müller, 2020) and *Rio. incertus* (Bonaparte & Pumares, 1995).

The rostral process of the lacrimal is mostly obscured by the overlapping nasal and prefrontal, hindering a proper measurement of the ramus itself and making a possible contact with the maxilla not visible. However, it is clear that the lacrimal is visible in dorsal view (Figs. 5 and 9), likely more rostrally placed than the prefrontal like *Pla. trossingensis* (Lallensack et al., 2021), *Luf. huenei* (Barrett et al., 2005), *Yun. huangi* (Barrett et al., 2007) and *Jin. xinwaensis* (Zhang et al., 2020), whereas in ventral view it laterally outreaches the level of the maxilla, but neither as prominently as in *Eor. lunensis* (Sereno et al., 2013) and *Bur. schultzi* (Müller et al., 2018a) nor forming a lacrimal knob as in some specimens of *Mas. carinatus* (Sues et al., 2004) and *Mel. readi* (Yates, 2007).

In lateral view, the rostroventral margin of the rostral process is characterized by an anteriorly opening notch that receives the caudolateral hook-like process of the nasal (Figs. 3 and 4), similarly to *Pla. trossingensis* (Lallensack et al., 2021). Moreover, as in most of the basal sauropodomorphs (e.g. Chapelle et al., 2019; Lallensack et al., 2021; Müller, 2020; Schaeffer, 2024), a rostrocaudally oriented ridge is present, delimiting the lateral surface from the dorsal one and fading caudally towards the rounded, convex caudolateral corner of the lacrimal.

An extensive, lateral flange flares rostroventrally extending from the ventral margin of the rostral process to the midshaft of the ventral ramus, covering the dorsocaudal corner of the antorbital fenestra and facing caudolaterally (Figs. 3, 4). A similar morphology was thought to be an autapomorphy of *Pla. trossingensis* (Yates, 2003a), however it is present in many other taxa, like *Iss. saaneq* (Beccari et al., 2021) and *Mac. itaquii* (Müller, 2020), whereas in *Efr. minor*, *Sel. gracilis* (Yates, 2003a) and in several massospondylids, e.g. *Sar. aurifontanalis* (Rowe et al., 2011) *Mas. carinatus* (Chapelle & Choiniere, 2018), *Ngw. intloko* (Chapelle et al., 2019), *Luf. huenei* (Barrett et al., 2005), *Ade. mognai* (Martínez, 2009), it is not as developed as in the aforementioned sauropodomorphs. The rostroventral margin of this lateral overhang is convex, as in some specimens of *Pla. trossingensis* (Lallensack et al., 2021), *Mac. itaquii* (Müller, 2020) *Ngw. intloko* (Chapelle et al., 2019) and *Luf. huenei* (Barrett et al., 2005), but it is concave in *Iss. saaneq* (Beccari et al., 2021) and *Mas. carinatus* (Chapelle & Choiniere, 2018).

In lateral view, the ventral ramus of the lacrimal is hourglass-shaped with a constriction at the midshaft that separates the dorsal half, that consists of the wide lateral flange, from the ventral half, which is rostrocaudally expanded towards the distal end (Figs. 3, 4) as in the majority of non-sauropodan sauropodomorphs, but not in *Sar. aurifontanalis* (Rowe et al., 2011). The caudal surface of this process is concave, as notable in the left lacrimal (Fig. 4), and its dorsal half articulates caudomedially with the lacrimal ramus of the prefrontal, as in *Pla. trossingensis* (Prieto-Márquez & Norell, 2011; Schaeffer, 2024).

The main shaft of the ventral ramus of the lacrimal is anteroposteriorly thin, as in several massospondylids like *Mas. carinatus* (Chapelle & Choiniere, 2018), and it is mostly formed by a rostroventrally oriented, concave ridge that distally curves caudoventrally, defining the caudal margin of the ventral half of the lacrimal as well as the rostroventral rim of the orbit similarly to *Bagualosaurus agudoensis* Pretto et al., 2019 and *Mac. itaquii* (Müller, 2020). This ridge fades rostroventrally into a medially recessed, triangular lamina that extends along the rostroventral third of the ventral process and contributes to the caudoventral corner of both the antorbital fossa and the antorbital fenestra as well (Figs. 3, 4). A fin-like lamina is present in almost all non-sauropodan sauropodomorphs, but it is absent in the massospondylid *Sar. aurifontanalis* (Rowe et al., 2011) and in some basal sauropodiforms, e.g. *Mel. readi* and *Anc. polyzelus* (Barrett, 2009; Yates, 2004, 2007). The rostral margin of the medial lamina is dorsocaudally-rostroventrally oriented and possesses a straight edge, comparable to non-massopodan sauropodomorphs, like some specimens of *Pla. trossingensis* (e.g. SMF 15.4; SMF 16.1; Lallensack et al., 2021), *Iss. saaneq* (Beccari et al., 2021) and *Mac. itaquii* (Müller, 2020), but not in most massospondylids, as *Mas. kaalae* (Barrett, 2009), *Ngw. intloko* (Chapelle et al., 2019), *Luf. huenei* (Barrett et al., 2005), *Ley. marayensis* (Apaldetti et al., 2011) and *Ade. mognai* (Rowe et al., 2011), in which it is concave.

Distally, the ventral ramus of the right lacrimal is laterally overlapped by the rostral process of the jugal, with the latter contributing to the ventral margin of the lacrimal contribution to the antorbital fossa and the caudalmost portion of the ventral margin of the antorbital fenestra as well (Fig. 3). This condition is exaggerated in the left lacrimal due to a rostral displacement of the left jugal (Fig. 4). A similar morphology is present in non-massopodan sauropodomorphs, like plateosaurids (Beccari et al., 2021; Lallensack et al., 2021; Schaeffer, 2024), in non-sauropodiform massopodans, like *Rio. incertus* (Bonaparte & Pumares, 1995) and massospondylids (Apaldetti et al., 2011; Barrett et al., 2005; Chapelle et al., 2019), and also in basal sauropodiforms (Pol & Powell, 2007; Yates, 2010; Yates et al., 2010).

### Prefrontal

The prefrontals are among the best-preserved bones in the entire skull, with the right element missing just the rostralmost portion of the dorsal lamina and the left one being minimally displaced rostroventrally (Fig. 5). In lateral view, as in the majority of sauropodomorphs, the prefrontal is characterized by a T-shaped outline accounting for a main rostrodorsal flange, a dorsocaudal process and a ventral process, with the latter two rami defining the rostrodorsal rim of the orbit (Figs. 3, 4). On the other hand, it is rhomboidal in dorsal view (Fig. 5), being relatively longer than wide, similarly to non-massopodan sauropodomorphs (e.g. Beccari et al., 2021; Lallensack et al., 2021; Müller, 2020) and not to the elongated forms of massospondylids and basal sauropodiforms (e.g. Apaldetti et al., 2011; Barrett et al., 2005, 2007; Chapelle & Choiniere, 2018; Martínez, 2009; Pol & Powell, 2007). However, it differs from non-massopodan sauropodomorphs in accounting for more than the 50%, specifically 55%, of the mediolateral width of the dorsal surface at the widest point of the skull and thus being closer to the stouter and wider morphologies of *Col. brevis* (Apaldetti et al., 2014) and *Mel. readi* (Yates, 2007), which contribute for the 60%.

The dorsal lamina is a dorsoventrally thin bony sheet, almost perpendicular to the lateral surface of the prefrontal, that extends both rostrally and medially. Possessing a flat-to-concave surface, it tapers towards the blunt, distal end where it slightly overlaps the nasal, in turn marking the medial margin of the additional caudolateral process of the latter bone (Fig. 5). Contrastingly to the distinguishing condition of *Pla. trossingensis* (Lallensack et al., 2021; Schaeffer, 2024), a rostrolateral notch on the anterior margin of the prefrontal is missing in SMF 13.5.37.

Even though the medial margin of the dorsal shelf of the prefrontal is not well-preserved and rather fragmented, it appears to be convex, thus marking a curved-to-straight medial contact with the nasal. In dorsal view, the left prefrontal reaches the same level of the lacrimal due to a rostral displacement, whereas in the right one it is not possible to determine it (Figs. 5, 9). However, it is likely that the prefrontal did not reach the dorsally exposed anterior tip of the lacrimal, like *Pla. trossingensis* (Lallensack et al., 2021).

The dorsal lamina expands mediolaterally while extending caudally and it extensively overlaps the lacrimal, reaching its widest extent at the caudalmost contact with the latter bone (Figs. 5, 9) as in *Yun. huangi* (Barrett et al., 2007). In lateral view, the dorsal shelf is continuous with the base of the ventral process and envelops the dorsocaudal corner of the lacrimal with a ventrally oriented, hook-like flange (Fig. 4). A comparable morphology is recorded in *Pla. trossingensis* (Lallensack et al., 2021; Prieto-Márquez & Norell, 2011; Schaeffer, 2024), *Mac. itaquii* (Müller, 2020) *Luf. huenei* (Barrett et al., 2005) and *Yun. huangi* (Barrett et al., 2007), but not in *Mas. carinatus* (Chapelle & Choiniere, 2018). Placed at the same height of the lacrimal ridge, a rostrocaudally oriented and weakly bulging ridge marks the boundary between the dorsal and the lateral surface of the prefrontal, and caudally it fades into the lateral margin of the frontal process.

In lateral view, the frontal process of the prefrontal is dorsoventrally thick proximally, whereas it thins while extending caudally, forming an overhanging, sharp ridge that delimits the rostrodorsal margin of the orbit (Figs. 3, 4), similarly to many sauropodomorphs, e.g. *Pla. trossingensis* and *Mas. carinatus* (Chapelle & Choiniere, 2018; Lallensack et al., 2021). In dorsal view, at the level of the lacrimal-prefrontal contact, the caudal ramus is mediolaterally broad, but suddenly tapers distally into a subtriangular, slightly concave process that is longer than the rostral lamina (Fig. 5), matching in morphology to non-massopodan sauropodomorphs, especially plateosaurids and unaysaurids (e.g. Müller, 2020; Schaeffer, 2024), and differing from the strap-like caudal process of non-sauropodiform massopodans and basal sauropodiforms (e.g. Barrett et al., 2005, 2007; Chapelle & Choiniere, 2018; Martínez, 2009; Yates, 2010). Roughly as wide as a fourth of the widest point of the prefrontal, the distalmost portion of the frontal process is subsquared and perfectly fits into a notch located on the lateral margin of the frontal, thus defining a relatively short prefrontal-frontal contact.

Given its relatively long caudal extent, the prefrontal reaches the midlength of the orbital diameter (Figs. 3, 4), differently from other non-massopodan sauropodomorphs like *Eor. lunensis*, *Bur. schultzi*, *Iss. saaneq* and *Mac. itaquii* (Beccari et al., 2021; Müller, 2020) and from the massospondylid *Ley. marayensis* (Apaldetti et al., 2011). However, contrasting most of the massospondylids and rather resembling *Pla. trossingensis* (Lallensack et al., 2021; Schaeffer, 2024; Yates, 2003a), *Luf. huenei* (Barrett et al., 2005) and *Mel. readi* (Yates, 2007), the prefrontal reduces the frontal contribution to the orbit. Nevertheless, both a complete exclusion of the frontal from the orbital margin and a prefrontal-postorbital contact are not recorded in SMF 13.5.37, differently from some specimens of *Pla. trossingensis*, e.g. AMNH FABR 6810 (Prieto-Márquez & Norell, 2011).

The lacrimal ramus of the prefrontal develops as a ventrally oriented splint-like process that articulates caudomedially with the caudal surface of the ventral process of lacrimal, extending along this latter for more than two-thirds of its length and bounding most of the rostral orbital margin (Fig. 4), as in the great majority of non-sauropodan sauropodomorphs, with the exceptions of *Mas. kaalae* (Barrett, 2009) and *Luf. huenei* (Barrett et al., 2005). In caudolateral view, given the weak degree of displacement and disarticulation of the left prefrontal, it is visible that the ventral half of the lacrimal ramus is twisted roughly at its midheight, leading the distal tip to be ventrolaterally projected, as in *Pla. trossingensis* (Lallensack et al., 2021; Prieto-Márquez & Norell, 2011), *Mac. itaquii* (Müller, 2020) and *Mas. carinatus* (Chapelle & Choiniere, 2018).

### Frontal

The right frontal is three-dimensionally preserved and still in articulation with the surrounding bones, whereas the left is rather crushed and highly fragmented, showing open sutures with the nasal and the prefrontal due to taphonomic displacement of the latter elements (Figs. 5, 9). In dorsal view, roofing most of the caudal half of the skull, each frontal has a subtrapezoidal shape, accounting for a rostral and a caudolateral process, with a length-to-width ratio of 1.47, thus being longer than wide as in most non-sauropodan sauropodomorphs except the massospondylid *Sar. aurifontanalis* (Rowe et al., 2011) and *Ngw. intloko* (Chapelle et al., 2019), in which the length is either subequal to or shorter than the width, respectively. Reaching its widest point at the anterior contact with the postorbital (Figs. 5, 9), like almost all basal sauropodomorphs, e.g. *Pla. trossingensis* (Lallensack et al., 2021), *Col. brevis* (Apaldetti et al., 2014) and *Luf. huenei* (Barrett et al., 2005), the frontal firstly gets constricted along its contribution to the orbital margin and then tapers rostrally at the level of the contact with the nasal and prefrontal. The medial interfrontal suture is interdigitated rather than straight (Figs. 5, 9), as in *Luf. huenei* (Barrett et al., 2005), *Mel. readi* (Yates, 2007) and partially in *Ley. marayensis* (Apaldetti et al., 2011), with each frontal being medially raised dorsally and forming a domed structure that runs along the entire length of the bone, from the rostralmost contact with the nasal to the caudal frontal-parietal suture, more prominently bowed than *Pla. trossingensis* (Lallensack et al., 2021; Prieto-Márquez & Norell, 2011; Schaeffer, 2024), *Mas. carinatus* (Chapelle & Choiniere, 2018), *Yun. huangi* (Barrett et al., 2007) and *Mus. patagonicus* (Pol & Powell, 2007). In dorsal view, the nasal ramus of the frontal is a subtrapezoidal, shallowly concave process that tapers rostrally and possesses two notches for the articulation with the nasal rostrally and the prefrontal caudolaterally, in turn defining a triangular flap in between the latter two bones (Figs. 5, 9). A protruding flange between the nasal and the prefrontal is present in some basal sauropodiforms, as *Jin. xinwaensis* and *Mel. readi* (Yates, 2007; Zhang et al., 2020), but being lager and more rostrally pointing. The nasal-frontal suture is more laterally defined by an exposed S-shaped interlocking contact (Figs. 5, 9), whereas medially it appears to be either straight or slightly concave or even slightly overlapping, however it is not clear due to scarce preservation. Differently from the indentation for the prefrontal on the rostrolateral margin of the frontal, which widely occurs in basal sauropodomorphs as either a slot or a sulcus (e.g. Barrett, 2009; Beccari et al., 2021; Chapelle & Choiniere, 2018; Lallensack et al., 2021; Martínez, 2009), the notch for the nasal appears to be unique of SMF 13.5.37 given that it is not present in any other taxa. Accordingly, all basal sauropodomorphs seem to have an overlapping nasal-frontal contact, whose extent can vary among different taxa, with possibly a rostral concavity for the reception of the medial side of the nasal, but neither being not exposed in dorsal view nor forming an S-shaped suture (e.g. Apaldetti et al., 2011). Müller (2020) referred to *Mac. itaquii* as possessing two distinct slots on the frontal, one of which for the caudolateral process of the nasal, being triangular-shaped in dorsal view. However, rather than interlocking with the frontal in a notch of the latter, the nasal of *Mac. itaquii* seems to be overlapping and defining a slightly oblique or transverse suture, as also reported by Müller (2020), as best seen in the specimen CAPPA/UFSM 0001b. For these reasons, the notch on the rostral ramus of the frontal for the caudolateral, peg-like process of the nasal, in combination with an unusual, dorsally exposed, S-shaped nasal-frontal suture, is herein considered as an autapomorphy of SMF 13.5.37.

In dorsal view, behind the indentation for the prefrontal, the lateral margin of the frontal becomes concave, reaching the constricted-most point at its midlength, from which, subsequently, it expands caudolaterally towards the postorbital ramus (Figs. 5, 9). In lateral view, this region corresponds to the contribution of the frontal to the dorsal orbital margin, which is dorsoventrally thin, slightly raised dorsolaterally and rostrocaudally short compared to most non-sauropodiform sauropodomorphs (Fig. 4) (e.g. Pol et al., 2012; Müller, 2020), especially massospondylids (e.g. Apaldetti et al., 2011; Chapelle & Choiniere, 2018; Chapelle et al., 2019; Martínez, 2009; Rowe et al., 2011), and basal sauropodiforms (e.g. Barrett et al., 2007; Pol & Powell, 2007; Wang et al., 2020; Zhang et al., 2020). Nonetheless, it is not as reduced as in *Pla. trossingensis* (Lallensack et al., 2021; Prieto-Márquez & Norell, 2011; Schaeffer, 2024), *Sel. gracilis* (Yates, 2003a), *Luf. huenei* (Barrett et al., 2005) and *Mel. readi* (Yates, 2007), but rather resembles the intermediate condition found in *Col. brevis* (Apaldetti et al., 2014). Caudally, the frontal broadens forming a subrectangular, lateral flange, namely the postorbital ramus, whose main axis is slightly longer than the one of the nasal ramus. In dorsal view, this caudolateral process possesses a weakly concave notch for the reception of the postorbital, being visible in the right element, whereas in the left one this area is highly fragmented (Figs. 5, 9). Despite an interlocking suture or a lap-joint cannot be precisely assessed to the frontal-postorbital contact, it is clear that, in lateral view, the frontal does not exclude the postorbital from the contribution to the dorsal margin of the orbit (Figs. 3, 4), as in plateosaurian sauropodomorphs, like *Pla. trossingensis*, *Mac. itaquii*, *Sar. aurifontanalis*, but differently from massospondylids (e.g. Apaldetti et al., 2011, 2014; Chapelle & Choiniere, 2018; Chapelle et al., 2019), except *Luf. huenei* (Barrett et al., 2005). Remarkably, a slender, finger-like flange placed rostrally to the frontal-postorbital contact is not present, contrasting the condition of some *Pla. trossingensis* specimens (e.g. SMF 15.4; SMNS 13200) (Lallensack et al., 2021; Schaeffer, 2024), *Mel. readi* (Yates, 2007) and *Xingxiulong chengi* Wang et al., 2017 (Wang et al., 2020). In dorsal view, the caudal margin of the frontal corresponds to the frontal-parietal suture, which is sharp and transverse and not interdigitated (Figs. 5, 9), thus not like *Bur. schultzi* (Müller et al., 2018a), *Pla. trossingensis* (Lallensack et al., 2021; Prieto-Márquez & Norell, 2011), *U. tolentinoi* (McPhee et al., 2019), *Col. brevis* (Apaldetti et al., 2014) but rather as in *Mas. carinatus* (Chapelle & Choiniere, 2018). Medial to the articulation with the postorbital and partially contributing to the definition of the frontal-parietal suture, a deep, mediolaterally oriented, oval depression opens caudally towards the supratemporal fenestra (Figs. 5, 9). Being sharply defined by raised margins, it represents the contribution of the frontal to the supratemporal fossa as well as the attachment point for the *M. pseudotemporalis superficialis* (Button et al., 2016), similarly to basal sauropodomorphs.

Nonetheless, SMF 13.5.37 remarkably differs from non-sauropodan sauropodomorphs in contributing to the rostral margin of the supratemporal fenestra (Figs. 5, 9), a condition shared only with *X. chengi* (Wang et al., 2020). According to this, the general condition of non-sauropodan sauropodomorphs consists in the exclusion of the frontal from the supratemporal fenestra due to either a postorbital-parietal or parietal-laterosphenoid contact (e.g. Apaldetti et al., 2011, 2014; Chapelle & Choiniere, 2018; Lallensack et al., 2021; Müller, 2020; Müller et al., 2018a). A putative contribution is referred also to Carnian taxa, e.g. *Eor. lunensis* (Sereno et al., 2013), however, given the poor preservation, it is impossible to clearly state it. The frontal articulates caudoventrally with the laterosphenoid, a contact visible in dorsocaudal view of the right element.

### Jugal

The jugals are well-preserved, displaying some minor fractures along the main rami in both bones and a low degree of deformation in the left one. The caudal half of the right element is slightly displaced ventrally, not properly contacting the postorbital and the quadratojugal (Fig. 3), whereas the left one is fully disarticulated and more rostrally placed than its life position due to taphonomy (Fig. 4). The jugal is a triradiate, Y-shaped bone with a dorsoventrally convex lateral surface, accounting for three main processes, namely the maxillary ramus, the postorbital ramus and the quadratojugal ramus.

Being the longest of the jugal rami, the maxillary ramus is a robust, tab-like process with subparallel margins that tapers distally, reaching the caudoventral corner of the antorbital fenestra (Figs. 3, 4) as in several non-sauropodiform sauropodomorphs, like *Bur. schultzi* (Müller et al., 2018a), *Pla. trossingensis* (Lallensack et al., 2021), *Rio. incertus* (Bonaparte & Pumares, 1995), *Sar. aurifontanalis* (Rowe et al., 2011), *Ngw. intloko* (Chapelle et al., 2019), *Col. brevis* (Apaldetti et al., 2014), *Luf. huenei* (Barrett et al., 2005), and some sauropodiforms as well, like *Anc. polyzelus* (Yates, 2010), *Aar. celestae* (Yates et al., 2010) and *Mus. patagonicus* (Pol & Powell, 2007). Visible in the right element, along the distal two-thirds, it articulates with the jugal ramus of the maxilla in a stepped, irregular caudoventrally-rostrodorsally oriented suture, whereas a dorsomedial contact with the lacrimal is established along the distalmost third (Fig. 3).

As most sauropodomorphs, except *Luf. huenei* (Barrett et al., 2005) and *Jin. xinwaensis* (Zhang et al., 2020), the dorsal margin of the rostral process extensively contributes to the ventral rim of the orbit, providing it with a dorsocaudally concave outline in lateral view, whereas it becomes straighter rostrally, with a faint apex at the lacrimal-jugal contact (Figs. 3, 4). Beneath the orbit, the jugal accounts for a minimum dorsoventral height that corresponds to the 12% of the rostrocaudal length of the jugal (measured from the rostral tip of the jugal to the anteroventral corner of the infratemporal fenestra), being comparable to non-massopodan sauropodomorphs rather than massospondylids (e.g. Apaldetti et al., 2011, 2014), in which it is closer to 20%. Moreover, the surface of the suborbital region is dorsoventrally swollen and bulging laterally, but neither forming a distinct ridge, as in *Pla. trossingensis* (Lallensack et al., 2021; Schaeffer, 2024), *Iss. saaneq* (Beccari et al., 2021), *Mac. itaquii* (Müller, 2020) and *Ngw. intloko* (Chapelle et al., 2019), nor a boss, like *Luf. huenei* (Barrett et al., 2005).

The postorbital ramus is a dorsocaudally projecting, triangular process with a concave-to-flat lateral surface that tapers distally (Figs. 3, 4). This process is the shortest and the stoutest of the jugal rami, as in almost all non-sauropodan sauropodomorphs, with the exception of *U. tolentinoi* in which it is the most developed (McPhee et al., 2019). Its rostral margin is concave and forms the caudoventral corner of the orbit, whereas the caudal margin is proximally concave and distally straight and contributes to the definition of both the rostroventral corner and the caudal half of the anterior margin of the infratemporal fenestra. Given the taphonomic displacement, a rostromedially oriented, lightly recessed flange is visible on the rostral margin of the left postorbital ramus, representing the articular facet for the caudoventral margin of the jugal ramus of the postorbital (Fig. 4), similarly to *Pla. trossingensis* (Prieto-Márquez & Norell, 2011) and *Mas. carinatus* (Chapelle & Choiniere, 2018). In lateral view, the dorsocaudal process diverges from the maxillary ramus by approximately 140° (left), 150° (right), like *Pla. trossingensis* (145°) (Prieto-Márquez & Norell, 2011) and *Iss. saaneq* (137°) (Beccari et al., 2021).

The quadratojugal ramus is a caudoventrally oriented, narrow process that is slightly shorter than the maxillary ramus and that forms an angle of 100° with the postorbital ramus, similarly to non-massopodan sauropodomorphs, like *Pla. trossingensis* (80–95°) (Prieto-Márquez & Norell, 2011), *U. tolentinoi* (70°) (McPhee et al., 2019), *Mac. itaquii* (90°) (Müller, 2020) and the massospondylid *Col. brevis* (90°) (Apaldetti et al., 2014), whereas most of massopodans and sauropodiforms account for more acute gaps, e.g. *Mas. carinatus* (40°) (Chapelle & Choiniere, 2018), *Ngw. intloko* (60°–70°) (Chapelle et al., 2019), *Luf. huenei* (50°) (Barrett et al., 2005), *Mus. patagonicus* (45°) (Pol & Powell, 2007), *Jin. xinwaensis* (50°) (Wang et al., 2020; Zhang & Yang, 1995). While extending caudally, it tapers to a needle-like tip, differently from the bifurcating morphologies of some specimens of *Pla. trossingensis* (Lallensack et al., 2021; Schaeffer, 2024). Furthermore, two-thirds of its extent are dorsally overlapped by the jugal ramus of the quadratojugal (Fig. 3), opposing the overlaying condition of massopodans (Martínez, 2009). Its dorsal margin is concave and defines part of the rostroventral corner of the infratemporal fenestra as well as half of the infratemporal bar, like *Pla. trossingensis* (Prieto-Márquez & Norell, 2011), and differently from either some massospondylids that account for two-thirds of it, e.g. *Ley. marayensis* (Apaldetti et al., 2011), *Ade. mognai* (Martínez, 2009), or the short contribution, roughly one-quarter, of sauropodiforms, like *Mus. patagonicus* (Pol & Powell, 2007) and *Mel. readi* (Yates, 2007). Contrasting the condition of non-massopodan sauropodomorphs and *Col. brevis* (Apaldetti et al., 2014), the rostroventral corner of the infratemporal fenestra reaches the rear half of the orbit (Fig. 3), as in the majority of massospondylids, like *Sar. aurifontanalis* (Rowe et al., 2011), *Mas. carinatus* (Chapelle & Choiniere, 2018), *Luf. huenei* (Barrett et al., 2005), and of sauropodiforms, like *Mel. readi* (Barrett, 2007), *Anc. polyzelus* (Yates, 2010) and *Aar. celestae* (Yates et al., 2010).

### Postorbital

The postorbitals are partially preserved, both showing a certain degree of fragmentation and displacement as well, specifically with the right one being slightly pushed up dorsocaudally (Fig. 3) and with the left bone being more rostroventrally placed, resulting in a rostrocaudal constriction of the orbital diameter (Fig. 4). As a consequence, neither of the two elements is in articulation with the respective jugal. Furthermore, the left postorbital is almost missing the entire caudal process.

The postorbital is a triradiate, Y-shaped bone accounting for three main rami. Being robustly built like *Pla. trossingensis* and *Col. brevis* (Apaldetti et al., 2014; Lallensack et al., 2021), but not as much as *Luf. huenei* (Barrett et al., 2005) or *Mel. readi* (Yates, 2007), it contacts the frontal rostromedially, the laterosphenoid ventromedially, the jugal caudoventrally and the squamosal caudomedially. Furthermore, it contributes to both the dorsocaudal and caudal margins of the orbit and separates the temporal fenestrae. In rostral view, the converging point of the three rami of the postorbital is more laterally inflated than the rest of the bone surface, resulting in a swollen apex and a C-shaped outline.

The frontal ramus is a tab-like process being slightly shorter than the ventral process, but longer than the caudal one, and it develops rostrodorsally in lateral view, whereas in dorsal view it is rostromedially oriented (Figs. 5, 9). It is dorsoventrally thin along its entire extent, but it mediolaterally widens distally, forming a rounded end, as in *Pla. trossingensis* (AMNH FARB 6810) (Prieto-Márquez & Norell, 2011), that slots into a caudolateral notch on the frontal, so defining the frontal-postorbital contact, whose nature, either an interlocking or overlapping contact, cannot be properly established though (see “Frontal” section) (Figs. 5, 9). This condition differs from both the bifurcated morphology present in basal sauropodomorphs, like some specimens of *Pla. trossingensis* (e.g. SMNS 13200, Schaeffer, 2024), *Iss. saaneq* (Beccari et al., 2021), *Mac. itaquii* (Müller, 2020), *Sar. aurifontanalis* (Rowe et al., 2011), *Luf. huenei* (Barrett et al., 2005) and the tapering distal end of massospondylids, e.g. *Mas. carinatus*, *Col. brevis* and *Ade. mognai* (Apaldetti et al., 2014; Barrett et al., 2005).

Visible in right dorsocaudal view, the frontal ramus articulates ventromedially with the laterosphenoid, whereas a parietal-postorbital contact seems to be absent. Accordingly, the frontal is not excluded from the supratemporal fenestra, contrasting the condition of almost all non-sauropodan sauropodomorphs (see “Frontal” section) (e.g. Apaldetti et al., 2011, 2014; Barrett et al., 2005, 2007; Chapelle & Choiniere, 2018; Lallensack et al., 2021; Martínez, 2009; Müller, 2020; Müller et al., 2018a; Rowe et al., 2011; Yates, 2007).

In dorsal view it forms the rostrolateral rim of the supratemporal fenestra (Fig. 5), whereas, in lateral view, the lateral margin of this process is continuous with the external edge of the frontal and is concave, defining both part of the dorsal margin and the dorsocaudal corner of the orbit (Figs. 3, 4), the latter of which is unornamented, differently from the rugose pattern present in *Pla. trossingensis* (Lallensack et al., 2021; Prieto-Márquez & Norell, 2011; Schaeffer, 2024). Furthermore, as in *Mas. carinatus*, *Ngw. intloko* (Chapelle et al., 2019) and *Mel. readi* (Yates, 2007), it projects laterally, forming a weakly raised, overhanging shelf over the orbit, but without developing a flange as in *Eor. lunensis* (Sereno et al., 2013), *Bur. schultzi* (Müller et al., 2018a) and *Mac. itaquii* (Müller, 2020).

The squamosal ramus is a mediolaterally slender, dorsoventrally broad process that tapers distally, defining a longer than wide, triangular outline in lateral view and being shorter than the frontal ramus (Fig. 3). Despite the distalmost end of the ramus is encased by the matrix, the dorsal rim of it is visible in medial view, confirming the decrease in height towards the squamosal. In dorsal view, it extends caudomedially forming the caudolateral margin of the supratemporal fenestra, providing the latter with a bowed outline (Figs. 5, 9). On the other hand, in lateral view, the ventral margin of the squamosal ramus marks both the rostrodorsal corner and the dorsal margin of the infratemporal fenestra (Figs. 3, 4).

Developing caudally above the postorbital midheight, it possesses a subhorizontal dorsal margin that diverges from the frontal ramus by 135° in the right element and 140° in the left one, being wider than *Pla. trossingensis* (110°) (Lallensack et al., 2021; Schaeffer, 2024) and *Mas. kaalae* (120°) (Barrett, 2009), but comparable to *Iss. saaneq* (134°–149°) (Beccari et al., 2021). As a consequence of the stepped dorsal margin of the postorbital, the supratemporal fenestra is exposed in lateral view (Fig. 3) as in most basal sauropodomorphs (e.g. Apaldetti et al., 2014; Barrett et al., 2007; McPhee et al., 2019; Prieto-Márquez & Norell, 2011; Yates, 2007). The squamosal-postorbital contact cannot be defined due to both the incompleteness of the left postorbital and the taphonomic displacement of the right squamosal.

Accounting for the longest extent among the postorbital rami, the jugal ramus is a rostroventrally oriented, tongue-like process with subparallel, convex-to-concave margins that gradually tapers distally into a sharp point (Figs. 3, 4). The dorsal half is rather slender and straight with a shallowly concave surface directed caudolaterally, differently from the more rostrally curved, flat ventral half which shows a curvature similar to those of non-massopodan sauropodomorphs, e.g. *Pla. trossingensis* (Lallensack et al., 2021) and *Mac. itaquii* (Müller, 2020), rather than the more acute morphology of massopodans, especially massospondylids (e.g. Apaldetti et al., 2011; Barrett et al., 2005, 2007; Chapelle & Choiniere, 2018; Martínez, 2009; Rowe et al., 2011). Despite both postorbitals are not in articulation with the respective jugals, it is clear that the distal caudoventral margin of the ventral process extensively overlapped the dorsocaudal margin of the postorbital ramus of the jugal, thus forming the concave, caudoventral margin of the orbit. The medial surface of the descending ramus is obscured by the matrix, therefore making not possible to check whether its transverse width is greater than its rostrocaudal length at midshaft or not.

### Parietal

The parietals are taphonomically deformed, each of which showing different distortion degrees. In detail, the right element has been lifted up dorsocaudally, resulting in an almost complete disarticulation, retaining only the contact with the right frontal, and being fragmented at multiple levels, especially at the proximal base of both the rostrolateral and caudolateral process, with the latter missing its dorsal half (Figs. 3, 4). On the other hand, the left parietal is rather well preserved, but its caudolateral process has been plastically displaced rostrally, being horizontal in dorsal view, thus noticeably constricting the rostrocaudal length of the left supratemporal fenestra and consequently switching the main axis of the latter from the longitudinal to the transverse one (Fig. 5).

Together forming an hourglass-shaped outline in dorsal view (Fig. 5), the parietals roof the caudalmost portion of the skull, each possessing a rostrolateral and a caudolateral process, with the former articulating with the frontal and the latter with the squamosal, the supraoccipital and the otoccipital. Ventral to the main body, a wide contact with the laterosphenoid is established.

Considering the displacement of the right element with a split line occurring along the bone medial margin, it is likely that the parietals were not fused together. Furthermore, there is no clear evidence of a possible sagittal crest at the medial contact between the parietals.

Rostrally, the parietal is mediolaterally expanded, accounting for a short, subtriangular rostrolateral process that develops perpendicularly from the main body (Fig. 5), being 1.9 times longer than the minimum width of the latter, less than *Pla. trossingensis* (Prieto-Márquez & Norell, 2011). Extending ventrolaterally, it tapers distally, reaching the midlength of the frontal contribution to the supratemporal fossa and without contacting the postorbital (see, “Frontal” and “Postorbital” sections), differently from the longer morphologies of non-sauropodiform sauropodomorphs, like *Bur. schultzi* (Müller et al., 2018a), *Pla. trossingensis* (Lallensack et al., 2021; Scheffer, 2024), *Iss. saaneq* (Beccari et al., 2021), *Mac. itaquii* (Müller, 2020), *Rio. incertus* (Bonaparte & Pumares, 1995), *Sar. aurifontanalis* (Rowe et al., 2011), *Mas. carinatus* (Chapelle & Choiniere, 2018), *Ngw. intloko* (Chapelle et al., 2019), *Col. brevis* (Apaldetti et al., 2014), *Luf. huenei* (Barrett et al., 2005), *Ley. marayensis* (Apaldetti et al., 2011), *Ade. mognai* (Martínez, 2009), and basal sauropodiforms, e.g. *Yun. huangi* (Barrett et al., 2007), *X. chengi* (Wang et al., 2020), *Jin. xinwaensis* (Zhang et al., 2020), *Anc. polyzelus* (Yates, 2010) and *Mel. readi* (Yates, 2007). Furthermore, the lateral surface of the rostrolateral process is vertically oriented and faces caudally, thus contributing to the caudal edge of the supratemporal fossa and to the rostral rim of the supratemporal fenestra (Fig. 5). Remarkably, this process does not take part to the floor of the supratemporal fossa, contrasting the condition of several basal sauropodomorphs that are characterized by a stepped, excavated eminence (e.g. Apaldetti et al., 2011, 2014; Chapelle et al., 2019; Lallensack et al., 2021; Martínez, 2009; McPhee et al., 2019; Müller, 2020; Müller et al., 2018a; Rowe et al., 2011).

The dorsal margin of the rostrolateral process, which corresponds to the rostral edge of the parietal, defines a transverse, straight frontal-parietal suture that is slightly elevated with respect to the dorsal surfaces of the two latter bones, differently from the concave or convex outlines of other taxa, like *Efr. minor* and *Mas. carinatus* (Chapelle & Choiniere, 2018). On the other hand, the ventral margin of it contacts the laterosphenoid horizontally.

In dorsal view, the parietal is mediolaterally constricted behind the rostrolateral flaring, reaching its minimum breadth at the midheight and providing the main body with a laterally concave outline (Fig. 5). Sloping rostroventrally in lateral view, the dorsal surface of the bone is flat and separated from the lateral surface by a weakly developed, longitudinal ridge, which marks also the medial boundary of the supratemporal fossa (Fig. 5). This ridge is concave and runs along the entire rostrocaudal length of the parietal, distally fading into the dorsal margin of the caudolateral process. Being continuous with the rostrolateral process, the lateral surface of the parietal is dorsoventrally tall and convex and laterally folds downwards, defining the medial wall of both the supratemporal fossa and fenestra (Figs. 3, 5).

Distally, the parietal is gently deflected caudolaterally and it develops as a dorsoventrally broad, mediolaterally flat wing-like lamina, namely the squamosal ramus, that is 1.4 times longer than the rostrocaudal length of the main body (Fig. 5), thus being slightly shorter than *Pla. trossingensis* (1.8, AMNH FARB 6810; 2.0, SMNS 13200) (Prieto-Márquez & Norell, 2011; Schaeffer, 2024). Bounding the caudal margin of the supratemporal fenestra, the caudolateral process decreases in height while extending ventrolaterally, being visible in caudal view. Furthermore, it widens distally, terminating with an oval articular facet for the squamosal that is preserved only in the left parietal (Fig. 4).

In dorsal view, the sharp dorsal margins of the caudolateral processes together form a rostrally-pointing, V-shaped outline of 145° in gap (Fig. 5). In detail, the right caudolateral process diverges from the main axis of the skull by an angle of 65°, whereas the left one by 80°. The discrepancy between the two rami is due to plastic deformation of the left element. Nonetheless, the right one, which seems to be only fragmented and dorsally displaced, bears an angle that is wider than the general condition of non-sauropodan sauropodomorphs, which is 45° (Apaldetti et al., 2011, 2014; Pol & Powell, 2007; Prieto-Márquez & Norell, 2011; Schaeffer, 2024; Zhang et al., 2020), but similar to the massospondylids *Ngw. intloko* (Chapelle et al., 2019) and *Luf. huenei* (Barrett et al., 2005).

In caudal view, the parietal articulates with the supraoccipital dorsocaudally, with the latter protruding in between the squamosal rami of the former (Fig. 6). Moreover, being visible only in the left element, the caudoventral surface of the caudolateral process is overlapped by the paroccipital process of the left otoccipital, leaving the dorsocaudal part exposed caudally. Neither the postparietal fenestra nor the posttemporal fenestrae can be detected due to taphonomic deformation.

### Squamosal

The squamosals are the least preserved cranial bones in SMF 13.5.37. In detail, the left element is ventromedially displaced within the deformed infratemporal fenestra and highly fragmentary, being completely shattered with only the caudoventral process recognisable (Fig. 4). On the other hand, the right bone is more complete, despite both missing the rostralmost portion of the rostrolateral process and the entire ventral process and presenting a longitudinal fracture along its dorsal surface. Furthermore, the right squamosal is tilted rostrodorsally and dislocated dorsocaudally, resulting in total disarticulation from the surrounding bones (Fig. 3).

The squamosal is a tetraradiate bone that bounds the dorsocaudal corner of the infratemporal fenestra and the caudolateral corner of the supratemporal fenestra as well (Fig. 3). Characterized by four distinct rami, it originally articulated rostrolaterally with the postorbital, rostromedially with the parietal, rostroventrally with the quadratojugal, caudoventrally with the quadrate and caudomedially with the otoccipital.

Present only in the right squamosal, the postorbital ramus is a proximally broad, mediolaterally thin, triangular lamina that faces dorsolaterally while extending rostrolaterally and that contributes to the supratemporal bar, defining both the dorsocaudal margin of the infratemporal fenestra and the caudolateral margin of the supratemporal fenestra (Fig. 3). Despite likely missing the rostralmost portion, thus hindering a proper measurement of its length, it appears to have been shorter than the rostromedial process. On the lateral surface, a shallow groove represents at least the caudalmost portion of the articular surface for the squamosal ramus of the postorbital, similar to several non-sauropodan sauropodomorphs, e.g. *Pla. trossingensis* (Lallensack et al., 2021; Prieto-Márquez & Norell, 2011) and contrasting the forked morphology of *Mas. carinatus* (Chapelle & Choiniere, 2018).

In the right squamosal, the parietal ramus is separated from the main body and slightly elevated with respect to the latter due to taphonomic displacement (Fig. 3). On the other hand, a putative, highly incomplete distalmost portion of the left parietal ramus is found medial to the base of the squamosal ramus of the left postorbital (Fig. 4). The parietal ramus is a mediolaterally thin, elongated tabular process that is rostromedially oriented, originally contributing to the caudal wall of the supratemporal fenestra. Being dorsoventrally high for almost its entire extent, it tapers distally, forming a triangular end (Fig. 3). Its caudal surface, which faces dorsocaudally, is characterized by a shallow concavity that corresponds to the articular surface for the caudolateral process of the parietal. In dorsal view, the dorsal margin of the parietal ramus is continuous with the rostrolateral process, defining the caudolateral corner of the supratemporal fenestra and forming a broad, U-shaped notch of roughly 80° in angle (Fig. 5). Despite this gap might have been exaggerated taphonomically, it is comparable to the massospondylids *Sar. aurifontanalis*, *Ngw. intloko*, *Col. brevis* and *Luf. huenei* (85°) (Chapelle et al., 2019), rather than the more acute condition present in Plateosauridae, e.g. *Pla. trossingensis* (60°) (Lallensack et al., 2021), *Iss. saaneq* (45°) (Beccari et al., 2021), and in the massopodans *Mas. carinatus* (50°) (Chapelle & Choiniere, 2018) and *Yun. huangi* (50°) (Barrett et al., 2007).

In dorsal view, behind the rostral processes, the right squamosal tapers mediolaterally towards its caudal end, whereas it folds ventrally to form the quadratojugal ramus in lateral view. Much of the descending process is missing, with only the broad, triangular proximal base being scarcely preserved. Fragments of the left ventral process of the squamosal are found scattered within the left infratemporal fenestra (Fig. 4).

Diverging perpendicularly from the quadratojugal ramus as in other basal sauropodomorphs (Apaldetti et al., 2014), the otoccipital ramus is a mediolaterally thin, subrectangular process that extends caudoventrally for a short distance with a caudomedial inclination of 45° to the sagittal plane. In lateral view, it possesses a blunt distal end (Fig. 3), differently from either the tapering or the hook-like morphologies present in other sauropodomorphs, like *Luf. huenei* and *Yun. huangi* (Barrett et al., 2005, 2007). On the other hand, the left caudoventral process misses its caudoventral portion, in turn having a sharp ventral margin that defines a triangular outline. Originally, the medial surface of this ramus contacted the notched lateral surface of the paroccipital processes of the otoccipital, however this is not clearly visible due to the high degree of disarticulation. The proximal region of the otoccipital ramus is characterized by a ventromedially recessed cavity for the reception of the head of the quadrate, namely the quadrate cotyle, which is also defined rostrally by the caudal margin of the descending process.

### Quadratojugal

The right quadratojugal is perfectly preserved, with just the distal end of the dorsal process missing its tip and being slightly displaced rostroventrally from the rest of the bone (Fig. 3). On the other hand, the left element is fragmented, with most of the lateral surface abraded, and having the rostrodorsal process partially obscured by the left jugal (Fig. 4).

The quadratojugal is a U-shaped bone encompassing the caudoventral corner of the infratemporal fenestra (Figs. 3, 4). Medially resting on the lateral surface of the quadrate, it accounts for a main body and two main rami, namely the jugal ramus and the squamosal ramus.

The main body of the quadratojugal is a rostroventrally wide, subtriangular lamina that extends caudoventrally into an expanded, rounded end that medially envelops the ectocondyle of the quadrate. Its lateral surface is featureless, with a convex proximal half and a concave distal half. A similarly well-developed caudoventral flange is present in *Pla. trossingensis* (Lallensack et al., 2021; Schaeffer, 2024), *Luf. huenei* (Barrett et al., 2005) and *Jin. xinwaensis* (Zhang et al., 2020).

The main body is marked by a convex caudal margin, continuous with the squamosal ramus, whereas the rostroventral margin is concave and forms a broad notch with the jugal ramus. The jugal ramus is a rostrodorsally oriented, triangular process with a shallowly concave lateral surface that tapers distally into a narrow point (Figs. 3, 4). In lateral view, differently from the slenderer morphologies of several basal sauropodomorphs (e.g. Chapelle & Choiniere, 2018; Müller, 2020), it is characterized by an inflated proximal base that represents the 55% and the 60% of the main body height, respectively in the right and left bone, contrasting *Pla. trossingensis* in which accounts only for the 35%, except for taphonomically deformed specimens that can reach the 50% (Lallensack et al., 2021; Schaeffer, 2024).

The dorsal margin of the jugal ramus is concave and forms both the caudal half of the ventral margin and part of the caudoventral corner of the infratemporal fenestra (Figs. 3, 4). Conversely, the ventral margin is convex and defines an extensive contact with the dorsal edge of the quadratojugal ramus of the jugal, overlaying it for at least two-thirds of its entire length and without reaching the orbit, differently from *Mel. readi* (Yates, 2007). Remarkably, the jugal-quadratojugal suture is comparable in many sauropodomorphs, but not in most massospondylids, like *Mas. carinatus* (Chapelle & Choiniere, 2018), *Ley. marayensis* (Apaldetti et al., 2011), *Ade. mognai* (Martínez, 2009), and sauropodiforms, e.g. *Yun. huangi* (Barrett et al., 2007), *X. chengi* (Wang et al., 2020), *Mel. readi* (Yates, 2007), in which the rostrodorsal process articulates with the ventrolateral margin of the jugal rather than the dorsal one.

The squamosal ramus is a proximally expanded, distally tapering process that is dorsally oriented, medially articulating with the quadrate and reaching the midshaft of the latter with a rounded distal tip (Figs. 3, 4). A possible contact with the squamosal cannot be defined due to taphonomic fragmentation.

Similar to *Eor. lunensis* (Sereno et al., 2013) and *Pla. trossingensis* (Schaeffer, 2024), it is characterized by a sinuous caudal margin, which is caudoventrally convex and dorsocaudally concave, and by a concave rostral margin, which partially defines both the caudoventral corner and the caudal rim of the infratemporal fenestra. The lateral surface of the ascending process is caudally concave and rostrally convex, likely following the shape of the quadrate (Figs. 3, 4).

Remarkably, despite being almost subequal in length, the squamosal ramus appears to be slightly longer than the jugal ramus as in *Eor. lunensis* (Sereno et al., 2013), contrasting the condition of the majority of non-sauropodan sauropodomorphs, in which the latter exceeds the former in extension (e.g. Chapelle & Choiniere, 2018; Lallensack et al., 2021; Langer & Benton, 2006; Martínez, 2009; Müller, 2020). Furthermore, the two quadratojugal rami diverge from each other with an angle of 90° in the left bone and 95° in the right element, similarly to the non-massopodan sauropodomorphs *Iss. saaneq* (84°) (Beccari et al., 2021) and *Mac. itaquii* (90°) (Müller, 2020), but also the massospondylids *Col. brevis* (80°) and *Ley. marayensis* (90°) (Apaldetti et al., 2011, 2014), as well as the sauropodiforms *X. chengi* (90°) (Wang et al., 2020) and *Mus. patagonicus* (80°) (Pol & Powell, 2007). On the other hand, *Pla. trossingensis* and most of the taxa belonging to Massospondylidae show more acute morphologies (Barrett et al., 2005; Chapelle & Choiniere, 2018; Chapelle et al., 2019; Martínez, 2009; McPhee et al., 2019; Prieto-Márquez & Norell, 2011; Rowe et al., 2011). The combination of both the perpendicular disposition of the two processes and the expanded web of bone in between results in a caudoventral corner of the infratemporal fenestra that does not extend as caudoventrally as in *Pla. trossingensis* (Lallensack et al., 2021; Prieto-Márquez & Norell, 2011; Schaeffer, 2024), *Ngw. intloko* (Chapelle et al., 2019), *Col. brevis* (Apaldetti et al., 2014) and *Luf. huenei* (Barrett et al., 2005).

### Quadrate

The right quadrate is well preserved, with just the rostrolateral flange being partially incomplete along its dorsolateral edge, and it is in its anatomical position, still in articulation with the right hemimandible (Fig. 3). Conversely, the left element is complete, but its shaft is slightly deformed, showing a more curved outline in lateral view and a straight medial margin due to taphonomic compaction. Furthermore, it is rostroventrally displaced and more anteriorly placed to the glenoid fossa of the left articular, as visible in caudomedial view (Fig. 4). Finally, none of the quadrates exhibit a proper contact with the respective squamosal, due to a scarce preservation of the latter, nor the rostromedial flange, which is laterally obscured by the matrix and medially covered by the pterygoid.

The quadrate is a robust, columnar bone that contacts the squamosal dorsally, the quadratojugal ventrolaterally and the pterygoid medially and that articulates ventrally with the articular, defining the craniomandibular joint. Forming the caudal margin of the entire skull, it accounts for a main shaft, a dorsal head, two distinct flanges and a double condylar surface.

In the right bone, the shaft is dorsoventrally elongated, being 1.37 times the dorsoventral height of the rostrum, a ratio that is higher only in *Bur. schultzi* and *Iss. saaneq* according to Beccari et al. (2021). On the other hand, this proportion cannot be properly calculated in the other quadrate given that the left side of the snout is highly deformed. Furthermore, the main body of the quadrate is mediolaterally thin along its dorsal two-thirds but widens while extending ventrally. The dorsalmost portion of it is rounded and forms the quadrate head that originally articulated with the quadrate cotyle present on the ventral surface of the squamosal. Taking into account that a lateral bony sheet is missing in the latter element, it is likely that the quadrate head was laterally exposed while contacting the squamosal, as in most basal sauropodomorphs.

In lateral view, the right quadrate shaft has a rostrally convex outline given by the rostrolateral flange, whereas the caudal margin is gently concave, almost straight (Fig. 3), contrasting the taphonomically arched left bone (Fig. 4). The more linear posterior outline does not match with the highly curving morphology of *Pla. trossingensis* (e.g. Lallensack et al., 2021), but rather does with several other non-sauropodan sauropodomorphs, especially massospondylids (e.g. Apaldetti et al., 2011, 2014; Chapelle & Choiniere, 2018; Chapelle et al., 2019). Differently, in caudal view, both quadrates show a sinuous caudomedial edge, which, in the undeformed right element, is laterally bowed at each extremity forming an angle of approximately 150° (Fig. 6) that is wider than the one in *Mas. carinatus* (135°) (Chapelle & Choiniere, 2018).

The caudal surface of the quadrate is convex along its ventral one-third, whereas the remaining dorsal two-thirds are shallowly concave. The inflection point is highlighted by the occurrence of a deep, dorsoventrally oriented fossa that is located laterally at the level of the midshaft, clearly visible in the left bone (Fig. 6). A similar feature is reported in *Mas. carinatus* (Chapelle & Choiniere, 2018).

Following the change in concavity, the caudolateral margin of the quadrate flares rostrolaterally, in turn defining a laterally projecting, thin flange that is more dorsoventrally developed than mediolaterally. Both the more lateral orientation and the reduced surface extent resemble the morphology of *Mac. itaquii* (Müller, 2020), but differ from the rostrolaterally expanded, D-shaped condition of *Pla. trossingensis* (Lallensack et al., 2021; Prieto-Márquez & Norell, 2011; Schaeffer, 2024). Its rostroventral margin articulates with the squamosal ramus of the quadratojugal, whereas its dorsolateral edge likely sutured with the quadratojugal ramus of the squamosal, despite this latter contact is not preserved.

In medial view, originating from the caudomedial margin of the bone, the quadrate possesses a wide, rostromedially oriented flange that extensively overlaps the quadrate wing of the pterygoid. Mostly obscured laterally by the encasing matrix, only the caudalmost portion of the medial surface of this process is visible in the left element, showing a larger, subtriangular extent compared to the rostrolateral flange, as in all sauropodomorphs. Furthermore, it appears that the two flaring processes diverge from each other almost perpendicularly, despite a proper angle cannot be established.

The ventralmost region of the quadrate possesses a double condylar surface that accounts for an entocondyle and an ectocondyle, which are respectively rostromedially and transversely oriented, together articulating with the glenoid fossa of the articular and being ventrally offset with respect to the maxillary tooth row. Moreover, the medial condyle exceeds the ventral extent of the lateral condyle, resulting in a more ventral placement than the latter (Fig. 6), as in several sauropodomorphs (e.g. Apaldetti et al., 2011, 2014; Beccari et al., 2021; Lallensack et al., 2021; Müller, 2020). However, this condition differs from some specimens of *Pla. trossingensis*, in which both the condyles are positioned at the same level (Chapelle & Choiniere, 2018; Prieto-Márquez & Norell, 2011).

A distinct quadrate foramen is not detected. Nonetheless, a possible opening is present in the left quadrate, precisely in between the lateral margin of the quadrate fossa and the medial margin of the quadratojugal (Fig. 6). Taking into account the degree of both disarticulation and distortion of the left side of the skull, this opening is herein considered as a taphonomic artifact rather than the quadrate foramen.

### Supraoccipital

The supraoccipital is fragmentary on the right half, which is additionally lifted dorsally, leading to a steeper dorsoventral inclination and a medial folding, whereas the left side is undistorted and properly placed anatomically (Fig. 6). The supraoccipital is a diamond-shaped bony plate that tapers both dorsally and ventrally, forming the dorsocaudal region of the braincase and the dorsal margin of the foramen magnum as well. It articulates dorsolaterally with the caudal surface of the caudolateral processes of the parietal and ventrolaterally with the base of the paroccipital processes of the otoccipital. The latter contact is marked by a straight suture, as in *Yun. huangi* (Barrett et al., 2007) and *Mel. readi* (Yates, 2007), that extends from the dorsolateral margin of the bone to the foramen magnum.

The supraoccipital is almost as high as wide, having a height-to-width ratio of 0.98 and reaching its maximum mediolateral breadth at one-third from the ventralmost margin, as in *Pla. trossingensis* (Lallensack et al., 2021). A similar proportion, with the width exceeding the height, is present in several non-massopodan sauropodomorphs (e.g. Galton & Kermack, 2010; Galton, 1985b; Martínez & Alcober, 2009; Müller et al., 2018a), in massopodans, as *Mas. carinatus* and *Col. brevis* (e.g. Apaldetti et al., 2014; Chapelle & Choiniere, 2018; Chapelle et al., 2019), and also in basal sauropodiforms, like *Jin. xinwaensis* and *Mus. patagonicus* (Pol & Powell, 2007; Zhang et al., 2020). However, given the low ratio, the condition of SMF 13.5.37 is also comparable to some specimens of *Pla. trossingensis* (Lallensack et al., 2021) and *Mel. readi* (Yates, 2007), in which both the vertical and horizontal dimensions are subequal to each other.

In left lateral view, it is rostrodorsally inclined with its dorsal apex reaching the level of the basipterygoid processes, sloping at an angle close to 45°–50° like *Pan. caducus* (Galton & Kermack, 2010), *Bur. schultzi* (Müller et al., 2018a), *Pla. trossingensis* (Galton & Upchurch, 2004; Lallensack et al., 2021; Scheffer, 2024), *Sar. aurifontanalis* (Rowe et al., 2011), *Col. brevis* (Apaldetti et al., 2014) and *Luf. huenei* (Barrett et al., 2005). However, this grade is exaggerated in the right half, being close to 70°, due to taphonomic displacement (Fig. 6).

**Fig. 10:**
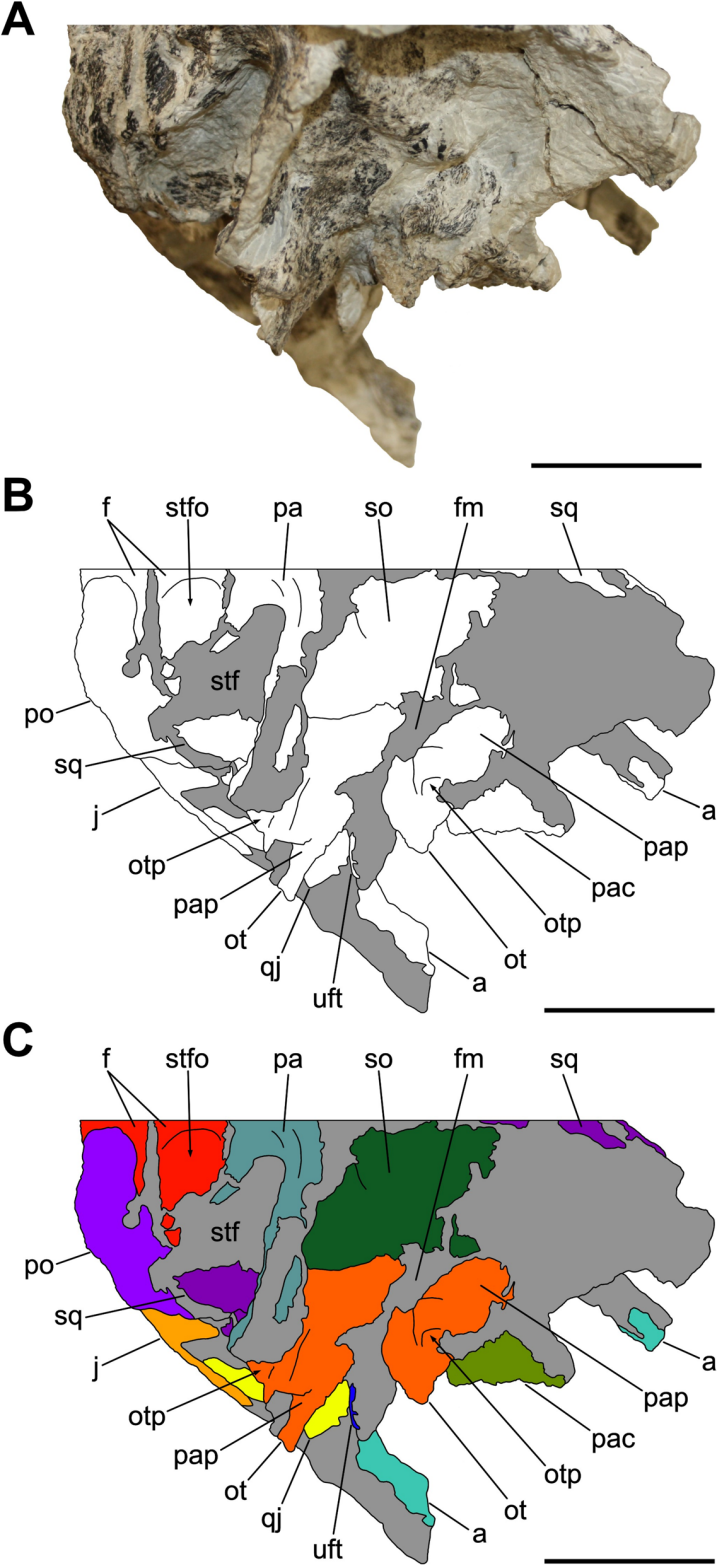
Close-up of the otoccipital autapomorphy of SMF 13.5.37 in left dorsomedial view. **A** Photograph. **B** Interpretative line drawing. White areas correspond to bones, grey surfaces represent matrix. **C** Coloured craniomandibular map. For abbreviations, see “Anatomical abbreviations” section. Scale bars equals 3 cm

Similar to many sauropodomorphs (e.g. Barrett et al., 2005, 2007; Chapelle & Choiniere, 2018; Schaeffer, 2024; Sereno et al., 2013; Wang et al., 2020; Yates, 2007; Zhang et al., 2020), the caudal surface of the supraoccipital possesses a longitudinal, dorsoventrally inflated median ridge that is laterally flanked by a shallow concavity on each side. This convexity corresponds to the nuchal crest, which is the insertion point for the nuchal ligaments (Figs. 6, 10).

The deformation of both the right side of the supraoccipital and the left parietal does not allow the detection of the postparietal fenestra (Knoll et al., 2012). Furthermore, no foramina for the *vena capitis dorsalis* are found.

### Otoccipital

The otoccipitals are mostly obscured by the matrix with only the paroccipital processes visible in caudal view. The left element is the most complete, even though it has been plastically displaced rostrally and fragmented in different points (Figs. 6, 10). Conversely, the right bone preserves exclusively the distal half of the paroccipital process, which is dislocated on top of the occipital condyle and exposed laterally in dorsal view (Figs. 6, 10). Based on Sampson and Witmer (2007), the otoccipital is formed by the co-ossified exoccipitals and opisthotics, as the case of SMF 13.5.37 in which no clear suture between the latter cited bones is visible.

The otoccipital forms the caudolateral region of the braincase and comprises an elongated caudolateral process, namely the paroccipital process, and a caudoventrally projecting process that respectively corresponds to the opisthotic and exoccipital contributions to the otoccipital. The former contacts the basioccipital dorsomedially, the parietal rostrodorsally and the squamosal rostrolaterally, whereas the latter articulates caudoventrally with the basioccipital.

The paroccipital process is a dorsoventrally high, mediolaterally thin wing-like process that extends caudolaterally with a weak ventral orientation. In dorsal view, it diverges from the sagittal plane of the skull with an angle of 80° (Fig. 6), being wider than any other sauropodomorph (e.g. Chapelle et al., 2019). However, this feature is highly exaggerated by a plastic deformation that led the entire process to be more rostrally placed. According to this, in lateral view, the distalmost end of it almost reaches the level of the basal tuberae, a condition not present in any sauropodomorph (Fig. 4). On the other hand, it seems more plausible that the paroccipital process was oriented parallelly to the caudolateral process of the parietal, thus having a sub-similar divergence angle of the latter, which is 65° (see “Parietal” section). A comparable orientation is only present in *Ngw. intloko* (Chapelle et al., 2019).

In caudal view, the paroccipital process is characterized by a horizontal ventral margin and a ventrally sloping dorsal margin that together provide the process with a distally tapering outline, specifically terminating in a dorsally rounded, ventrally blunt end (Fig. 6). Nonetheless, the left paroccipital process possesses a distally stepped, partially concave dorsal margin, possibly attributable to either a taphonomic deformation or a breakage (Fig. 6).

The lateral surface of the paroccipital process is marked by a distal concavity that represents the articular surface for the squamosal. Noteworthily, the latter is dorsally bordered by an unusual triangular spur that develops perpendicularly from the dorsal margin of both the paroccipital processes, clearly visible in the right one (Figs. 6, 10). The dorsal surface of this additional projection likely defines the articular surface for the parietal. A similar protruding flange is absent in all other known sauropodomorphs, which rather show either a flat or a slightly convex lateral surface of the paroccipital process. For this reason, this rostrolaterally projecting, triangular process is herein considered as an autapomorphy of SMF 13.5.37.

The proximomedial surface of the paroccipital process greatly defines the *foramen magnum*, forming the dorsolateral margin of it (Figs. 6, 10). A comparably extensive contribution is present in *Mel. readi* (Yates, 2007), which in turn leads to a consistent reduction of the supraoccipital contribution to the *foramen magnum*, whereas most sauropodomorphs show an opposite condition (e.g. Bronzati & Rauhut, 2018). Laterally to the occipital opening, a rounded ridge extends for at least half the length of the paroccipital process, horizontally dividing the medial surface in two distinct regions, the dorsal of which being deeply concave and representing the articular surface for the proatlas (e.g. Martínez, 2009; Müller et al., 2018a; Wang et al., 2020), differently from the ventral one that is slightly convex (Figs. 6, 10).

Despite being partially covered by the displaced right paroccipital process, the left otoccipital extends caudoventrally from the proximomedial surface forming a pyramidal process that contacts the basioccipital ventromedially and contributes to the dorsolateral portion of the occipital condyle, as in many sauropodomorph taxa. In caudal view, despite a clear suture in not visible, a faint groove on the occipital condyle possibly marks the otoccipital-basioccipital contact. A knob-like bony structure present on the right side of the occipital condyle is tentatively referred here as the caudoventral process of the right otoccipital, being isolated and ventrally displaced.

### Basioccipital

The basioccipital is almost completely obscured either by the matrix or by other skeletal elements, with only part of the occipital condyle and the basal tuberae visible in caudal view (Fig. 6). It contacts the otoccipital dorsolaterally and the basisphenoid ventrally.

The occipital condyle is poorly preserved, retaining only the left half, and it is mostly covered by the disarticulated right paroccipital process dorsally and by the proatlas-atlas complex dorsocaudally (Fig. 6). In left caudolateral view, it possesses a subcrescentic, mediolaterally convex outline that is defined by the basioccipital both ventrally and dorsomedially and by the pyramidal process of the otoccipital dorsolaterally, as in most basal sauropodomorphs (Bronzati & Rauhut, 2018). Contrastingly, the right caudoventral portion of the condyle is rather crushed and depressed rostrally, despite maintaining a convex surface (Fig. 6). A similar distortion is referrable as a taphonomic artifact rather than an anatomical feature.

In lateral view, the occipital condyle is proximally constricted, marking the distalmost portion of the condylar neck, whereas it protrudes caudally, slightly exceeding the level of the basal tuberae and thus differing from the elongated morphology of some basal sauropodomorphs, like *Pla. trossingensis* and *The. antiquus* (Ballell et al., 2020). Furthermore, despite most of the caudal surface of the basioccipital is obscured by the matrix, the occipital condyle clearly lies above the basioccipital tuberosities, which are more ventrally offset, forming a stepped lateral margin as in other taxa (Fig. 6), like *Pla. trossingensis*, *U. tolentinoi*, *Col. brevis*, *Luf. huenei* and *Mel. readi* (Apaldetti et al., 2014; McPhee et al., 2019).

The basal tuberae are two caudoventrally projecting, knob-like processes that are formed by the ossification of the basioccipital and the basisphenoid, with the former defining most of the caudal structure and the latter partially contributing to the proximal rostroventral region (Fig. 6), as visible in caudal view, as in many non-sauropodiform sauropodomorphs (e.g. Apaldetti et al., 2014). Possessing a rhomboid-shaped caudal surface, they represent the attachment point for the hypaxial neck musculature (e.g. Galton & Kermack, 2010; Romer, 1956; Snively & Russell, 2007).

In contrast to the majority of non-sauropodiform sauropodomorphs, in which either a transverse ridge or a shallow notch connects the medially expanded tuberosities (e.g. Ballell et al., 2021; Bronzati & Rauhut, 2018; Chapelle & Choiniere, 2018; Martínez, 2009; Müller, 2020; Rowe et al., 2011; Schaeffer, 2024; Yates, 2003a), the basioccipital component of the basal tuberae are widely separated by a deep, U-shaped notch (Figs. 6, 7) as in *Luf. huenei* (Barrett et al., 2005), *Anc. polyzelus* (Yates, 2004) and eusauropods (e.g. Madsen et al., 1995). Even though a pronounced medial groove rarely occurs also in *Pla. trossingensis* (e.g. SMF 07.M; Lallensack et al., 2021), this taxon differs from SMF 13.5.37 in having four distinct projections of the basal tuberae (Bronzati & Rauhut, 2018; Chapelle & Choiniere, 2018; Lallensack et al., 2021; Prieto-Márquez & Norell, 2011; Schaeffer, 2024; Yates, 2004). Furthermore, Bronzati and Rauhut (2018) tentatively referred a multituberculate morphology also to *Pan. caducus*, *U. tolentinoi*, *Ade. mognai* and *Mel. readi*. Despite being highly fragmented, a rostrally recessed, median ridge might be present, bordering the caudomedial surface of the basisphenoid recess, analogously to *U. tolentinoi* (McPhee et al., 2019), *Pla. trossingensis* (SMNS 13200; Schaeffer, 2024), *Mas. carinatus* (Chapelle & Choiniere, 2018) and *Luf. huenei* (Barrett et al., 2005).

### Basisphenoid

With only the caudalmost region visible in both caudal and ventral views, the basisphenoid forms the caudoventral portion of the braincase, accounting for the basal tuberae component, the basipterygoid processes, the basisphenoid recess and the subsellar recess (Figs. 6, 7). The dorsocaudal surface contributes to the rostroventral portion of the basal tuberae as a pair of caudolaterally oriented, shortly extending processes that articulate with the caudoventral surface of the basioccipital (Figs. 6, 7). Although the basioccipital-basisphenoid suture cannot be further investigated due to both taphonomic breakage and encasing sediment, it is clear that the ventral surface of the tuberosities does not bear any longitudinal striations or rugosities, otherwise present in *Pla. trossingensis* (Prieto-Márquez & Norell, 2011; Schaeffer, 2024).

Opposite to most of non-sauropodan sauropodomorphs (e.g. Barrett, 2009; Bronzati & Rauhut, 2018; Chapelle & Choiniere, 2018; Chapelle et al., 2019; Lallensack et al., 2021; Marsh & Rowe, 2018; McPhee et al., 2019; Prieto-Márquez & Norell, 2011) the relative contribution of the basisphenoid to the basal tuberae is rather reduced compared to the basioccipital, similar to *The. antiquus* and *Mel. readi* (Ballell et al., 2020, 2021; Yates, 2007). Accordingly, given the smaller caudolateral extent, the basisphenoid tuberosities develop perpendicularly from the caudal surface in lateral view, as in *Pla. trossingensis*, whereas a U-shaped outline is established in taxa, like *Mas. carinatus*, with a more caudoventrally expanded morphology (Chapelle & Choiniere, 2018).

Beneath the basal tuberae, the caudal surface of the basisphenoid extends ventrally as a subrectangular bony sheet that is characterized by a dorsomedial shallow fossa that opens caudally, namely the basisphenoid recess (Fig. 7) (*sensu* Witmer, 1997). Commonly occurring in Sauropodomorpha (e.g. Bronzati & Rauhut, 2018; McPhee et al., 2019), the basisphenoid recess is encompassed by the caudolateral margin of the basal tuberae, although differently from the deeply nested of *Mas. carinatus* (Chapelle & Choiniere, 2018) and the laminae-bordered of *Col. brevis* (Apaldetti et al., 2014), but rather comparable to the shallow oval depression of *Pla. trossingensis* and *Efr. minor* (Bronzati & Rauhut, 2018). However, contrasting the two latter taxa, the median fossa of SMF 13.5.37 does not reach the protuberance at the base of the basipterygoid processes, instead fades ventrally up to the midheight between the four basisphenoid processes.

Caudoventrally, set well-below to the basal tuberae, the basisphenoid possesses a pair of caudoventrally directed, elongated processes referred as basipterygoid processes that diverge at an angle close to 100° in caudal view (Figs. 6, 7), being wider than most basal sauropodomorphs (Chapelle & Choiniere, 2018), although comparable to *Ngw. intloko*, *Col. brevis* and *Ley. marayensis* (Apaldetti et al., 2011, 2014; Chapelle et al., 2019). In ventral view, the lateral orientation of both the basal tuberae and the basipterygoid processes provides the basisphenoid with an X shape (Fig. 7), which has been reported also in *Bur. schultzi* (Müller et al., 2018a), *Pan. caducus* (Galton & Kermack, 2010) and *Efr. minor* (Bronzati & Rauhut, 2018).

In lateral view, the basipterygoid processes project ventrally as in *The. antiquus*, *Mas. carinatus*, *Luf. huenei*, *Ade. mognai*, differently from either the caudoventral orientation of *Pla. trossingensis*, *Mas. kaalae*, *Ngw. intloko* and *Col. brevis* or the rostroventral inclination of *Pan. caducus*, *Efr. minor*, *Iss. saaneq*, *U. tolentinoi*, *Rio. incertus*, *Sar. aurifontanalis* and *Ley. marayensis* (Apaldetti et al., 2014; Beccari et al., 2021; Bronzati & Rauhut, 2018; Chapelle et al., 2019).

The right process is rostrocaudally stout, mediolaterally thin and expanded at its distal end, similarly to several sauropodomorphs (Fig. 6), like *Pla. trossingensis* (Prieto-Márquez & Norell, 2011), *Rio. incertus* (Bonaparte & Pumares, 1995), *Mas. carinatus* (Chapelle & Choiniere, 2018) and *Ade. mognai* (Martínez, 2009). On the other hand, the left process appears to be bulkier dorsoventrally due to taphonomic deformation, which, additionally, led the distal end to be incomplete and crushed onto the medial surface of the left pterygoid (Fig. 6).

In caudal view, the caudomedial margins of the basipterygoid processes converge dorsally at their proximal bases forming a sharp, dorsally pointing V-shaped ridge that has a rounded protuberance at its apex (Fig. 6), like *Efr. minor*, *Pla. trossingensis*, *U. tolentinoi and Ade. mognai* (Bronzati & Rauhut, 2018). As *Col. brevis* (Apaldetti et al., 2014), SMF 13.5.37 differs from several non-sauropodan sauropodomorphs (e.g. Chapelle & Choiniere, 2018; Marsh & Rowe, 2018), in lacking a web of bone spanning in between the basipterygoid processes, especially *Pla. trossingensis* that possesses an additional caudoventrally projecting, median extension (Prieto-Márquez & Norell, 2011; Schaeffer, 2024).

Rostrally, the ridge interconnecting the basipterygoid processes corresponds to the caudal surface of a caudoventral depression that represents the subsellar recess (Figs. 6, 7) (*sensu* Witmer, 1997). Medially defined by the concave, ventral surface of each basipterygoid process, this fossa is rostrocaudally longer than mediolaterally wide, oval in shape and remarkably shallow, contrasting the deep condition of other basal sauropodomorphs, like *Efr. minor* (Bronzati & Rauhut, 2018) and *The. antiquus* (Ballell et al., 2020, 2021).

### Laterosphenoid

The lateral and rostrocaudal surfaces of the right laterosphenoid are the only portions accessible, being visible in caudolateral view. The laterosphenoid forms the rostral region of the braincase, accounting for a main rostrolateral process and articulating with the parietal dorsally and dorsolaterally, with the frontal rostrodorsally and with the postorbital laterally as in most non-sauropodan sauropodomorphs (Fig. 5). Further caudal contacts with other neurocranial bones cannot be defined given the poor preservation of the bone itself and the encasing matrix. Noticeably, the laterosphenoid is not visible in dorsal view as it is completely subtended by other cranial bones.

The lateral surface of the laterosphenoid is vertical and dorsally continuous with the parietal, contributing to the medial wall of the supratemporal fenestra. Rostrally, it becomes concave while curving laterally to form the postorbital ramus.

The postorbital ramus is a laterally flaring, subtriangular process that develops perpendicularly from the main body of the laterosphenoid, defining an L-shaped curvature in dorsal view. Comparably, its mediolateral extension is closer to *Pla. trossingensis* (Lallensack et al., 2021; Prieto-Márquez & Norell, 2011; Schaeffer, 2024) than to the shorter morphologies shown in *Efr. minor* (Bronzati & Rauhut, 2018), *Mac. itaquii* (Müller, 2020), *Mas. carinatus* (Chapelle & Choiniere, 2018) and *Ngw. intloko* (Chapelle et al., 2019). Having a concave caudal surface that marks the rostral wall of the supratemporal fenestra, the rostrolateral process tapers in height distally to contact the caudomedial region of the frontal ramus of the postorbital, whereas it is proximally overlapped by the rostrolateral process of the parietal and centrally by the frontal contribution to the supratemporal fossa.

### Pterygoid

The pterygoids are accessible in caudal and ventral views, having only their caudomedial surfaces exposed (Figs. 6, 7). Despite being well preserved, both elements are rostroventrally displaced, particularly with the right bone slightly disarticulated from the quadrate, resulting in a more oblique orientation than the vertically oriented left one. Forming the caudalmost portion of the palatal complex, each pterygoid contacts the respective quadrate caudolaterally and accounts for three main rami, specifically a caudal process, namely the quadrate wing, a ventrolateral process and a rostral process (Figs. 6, 7).

Articulating with the medial surface of the rostromedial process of the quadrate, the quadrate ramus develops from a constricted, neck-like medial base as a vertical, caudolaterally expanding process which consists of a dorsoventrally broad, mediolaterally thin lamina that tapers dorsally into an elongated triangular flange. Even though the caudal margin of the quadrate wing is shallowly concave, it does neither bifurcate as in *Pla. trossingensis* (Galton, 1984, 1895a; Prieto-Márquez & Norell, 2011; Schaeffer, 2024) and *Ade. mognai* (Martínez, 2009) nor triradiate as in *Mas. carinatus* (Chapelle & Choiniere, 2018), but rather is defined by a single unit like *Mac. itaquii* (Müller, 2020).

Noticeably, in medial view, the ventral margin of the quadrate ramus is dorsocaudally oriented and stepped, marked by a shortly extending, caudoventrally projecting, convex bump instead of being horizontal and straight as in many basal sauropodomorphs (e.g. Chapelle & Choiniere, 2018; Chapelle et al., 2019; Müller, 2020). Nonetheless, a similar condition is shared with *Pla. trossingensis*, as visible in the specimens SMNS 12949 (Galton, 1985a), SMNS 13200 (Huene, 1926; Schaeffer, 2024) and AMNH FARB 6810 (Prieto-Márquez & Norell, 2011).

The medial surface of the quadrate wing is mostly flat but becomes concave while curving medially to merge with the rest of the pterygoid. At the level of the proximal dorsoventral constriction, two distinct concavities are present, divided from each other by a horizontal ridge. The ventral one is continuous with the caudal surface of the ventrolateral process, whereas the dorsal one is medially flanked by a short flange (Figs. 6, 7).

Ventral to the quadrate ramus, the pterygoid flares ventrolaterally forming a caudally curving, subtriangular process with a strongly concave posterior surface that tapers distally (Figs. 6, 7). Although the rostrocaudal extent cannot be defined, this process is dorsoventrally shorter and more inflated mediolaterally compared to the one of other non-sauropodan sauropodomorphs, like *Pla. trossingensis* (Prieto-Márquez & Norell, 2011; Schaeffer, 2024), *Iss. saaneq* (Beccari et al., 2021), *Luf. huenei* (Barrett et al., 2005) and *Ade. mognai* (Martínez, 2009).

In the left pterygoid, medial to the dorsal concavity of the quadrate ramus and dorsal to the caudolateral process, a dorsoventrally flat, semicircular flange shortly extends caudomedially, forming a lateral gap with the quadrate (Figs. 6, 7). The orientation of this tab-like projection might have been slightly different, given that it seems both detached and dorsally displaced from its base. On the other hand, the right contralateral element is missing in the right pterygoid. A similar, but longer process occurs in *Mas. carinatus* (Chapelle & Choiniere, 2018), *Ngw. intloko* (Chapelle et al., 2019) and *Ade. mognai* (Martínez, 2009), whereas shorter morphologies closer to SMF 13.5.37 are widespread among non-sauropodan sauropodomorphs, e.g. *Mac. itaquii* (Müller, 2020), *Sar. aurifontanalis* (Rowe et al., 2011), *Luf. huenei* (Barrett et al., 2005).

A homologous, hook-like process is present in all non-eusauropodan sauropodomorphs (e.g. Wilson, 2002; Wilson & Sereno, 1998) and, together with the proximal base of the quadrate wing, wraps around the distal end of the respective basipterygoid process of the basisphenoid. Unfortunately, this contact cannot be evaluated in SMF 13.5.37 given the evident ventral dislocation of both pterygoids. In ventromedial view, the medialmost region of the pterygoid possesses a short, rostrally oriented process that represents the proximal base of the anterior palatal ramus, which is completely obscured by the matrix (Fig. 7).

### Sclerotic ring

A semiarticulate series of six subrectangular, plate-like ossicles form an oval-shaped sclerotic ring placed at the rostroventral corner of the right orbit, partially obscuring both the lacrimal and the jugal (Fig. 3). The precise number of individual units and their articulation pattern cannot be defined due to both disarticulation and taphonomic compaction.

Remarkably, the absence of the sclerotic ring in the left orbit is likely determined by a longer exposure time prior to the burial than the right side of the skull, as occurs in some specimens of *Pla. trossingensis* from Trossingen (e.g. Schaeffer, 2024). Accordingly, the right part of the cranium was found bottom-facing in the field, indicating at least a rapid deposition on the sediment that resulted in a better preservation (Fig. 2).

### Dentary

The dentaries are partially fragmented due to both several minor fractures occurring obliquely along the main axes and a major cut that subdivides each bone into two main units. In detail, the right dentary is characterized by the dislocation of its rostralmost portion up to the third alveolus, which in turn is slightly ventromedially oriented (Fig. 3). On the other hand, the left element is slightly deformed, possessing an additional, rostroventrally oriented ridge along its symphyseal portion that is herein considered as a taphonomic artifact originated from a longitudinal breakage (Fig. 4).

**Fig. 11:**
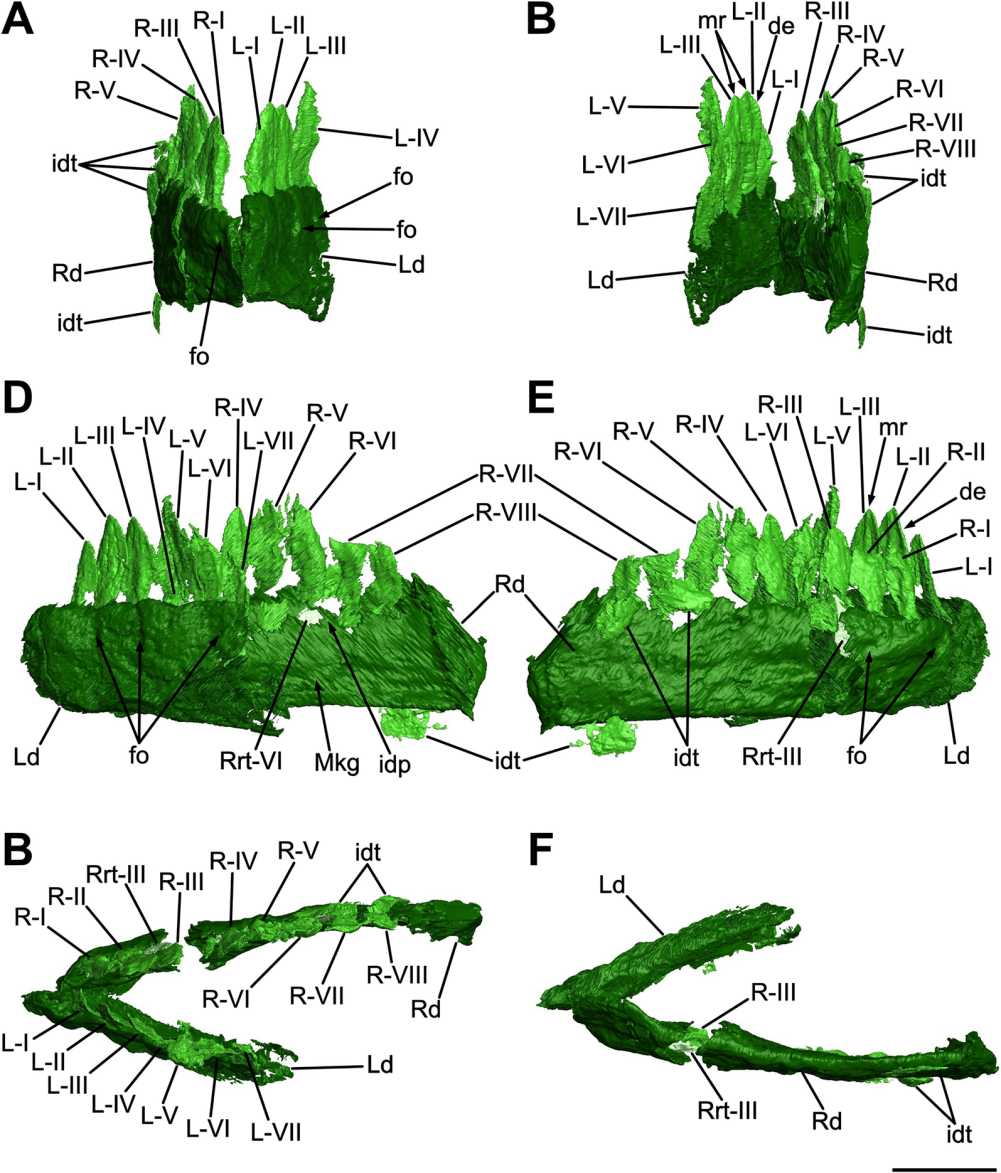
Three-dimensional rendering of the segmented dentaries and related teeth of SMF 13.5.37. **A** Rostral view. **B** Caudal view. **C** Left lateral view. **D** Right lateral view. **E** Dorsal view. **F** Ventral view. For abbreviations, see “Anatomical abbreviations” section. Scale bar equals 2 cm

The dentary is a rostrocaudally elongate, mediolaterally compressed tabular bone that contacts its contralateral equivalent rostromedially, the splenial medially, the angular caudoventrally and the surangular dorsocaudally, and bounds the rostral margin of the external mandibular fenestra (Figs. 3, 4). The rostral articulation between the dentaries forms a V-shaped mandibular symphysis that accounts for a divergence angle of roughly 40° in ventral view, as generally occurring in non-sauropodan sauropodomorphs, like *Mus. patagonicus* (43°) (Pol & Powell, 2007) (Figs. 7, 11F).

Contributing for more than the 60% of the entire mandibular length as in *Pla. trossingensis* (Prieto-Márquez & Norell, 2011), *Mac. itaquii* (Müller, 2020), *Ade. mognai* (Martínez, 2009), the dentary possesses a subrectangular shape that deepens while extending caudally, defining a posterior margin that is almost twice the height of the symphyseal region (Figs. 3, 4) as in several basal sauropodomorphs (e.g. Apaldetti et al., 2014; Barrett et al., 2005, 2007; Lallensack et al., 2021; Martínez, 2009; Schaeffer, 2024). Furthermore, SMF 13.5.37 shows a height-to-length ratio of the dentary equal to 0.22 and 0.23, respectively for the right and left dentary, being comparable to the stouter and shorter morphologies of massopodans (e.g. Apaldetti et al., 2011, 2014; Barrett, 2009; Pol & Powell, 2007; Yates, 2007; Zhang et al., 2020) rather than the slenderer ones of non-massopodan sauropodomorphs, especially plateosaurids and unaysaurids (e.g. Beccari et al., 2021; Müller, 2020).

In lateral view, the dentary is subrectangular shaped with a straight dorsal margin, whereas the ventral is slightly arched and gradually diverging from the alveolar border, thus leading the bone height to increase caudally, as in many basal sauropodomorphs. Anteriorly, the symphyseal region is rostroventrally downturned by 20° with respect to the posterior half of the dentary (Figs. 3, 4, and 11C, D), similarly to *Pla. trossingensis* (Lallensack et al., 2021; Prieto-Márquez & Norell, 2011; Schaeffer, 2024; Yates, 2003a). Remarkably, the rostralmost portion of the dentary is blunt and rounded as in *Sar. aurifontanalis* (Rowe et al., 2011), *Mas. carinatus* (Sues et al., 2004) and *Col. brevis* (Apaldetti et al., 2014) and lacks the strongly procumbent anterior margin present in *Arc. pereirabdalorum* (Yates et al., 2011), *Iss. saaneq* (Beccari et al., 2021), *Mas. kaalae* (Barrett, 2009), *Ngw. intloko* (Chapelle et al., 2019), *Ade. mognai* (Martínez, 2009), *Yun. huangi* (Barrett et al., 2007) as well as the ventral expansion found in *Pla. trossingensis* (e.g. Schaeffer, 2024), *U. tolentinoi* (McPhee et al., 2019), *Mac. itaquii* (Müller, 2020), *Rio. incertus* (Bonaparte & Pumares, 1995), *Mel. readi* (Yates, 2007) and *Mus. patagonicus* (Pol & Powell, 2007).

The lateral surface of the dentary is flat and rostrally pitted by a set of large, circular neurovascular foramina that open rostrally. In detail, the left bone accounts for a series of three evenly spaced foramina that are arranged linearly beneath the alveolar margin, whereas the right possesses only two, which are vertically placed close to the symphyseal end (Figs. 3, 4, 11C, D). Additionally, the µCT output unveiled the presence of several microforamina scattered on the anterior half of the right dentary, all being connected to a main neurovascular canal located in between the external surface and the alveoli. This cavity, as well as its branches, can be interpreted as part of the neurovascular system that housed the mandibular branch of the trigeminal nerve and related arteries and veins.

In dorsolateral view, the dorsal margin of the lateral surface of the dentary folds medially at the level of the 11th/12th alveolus, forming a faint ridge that gradually becomes more laterally prominent while extending caudally towards the contact with the surangular, distally flushing into the coronoid eminence (Figs. 3, 4). In detail, this ridge is caudodorsally oriented and forms a horizontal plateau lateral to the alveolar margin that leads the tooth row to be medially inset. A dorsocaudal, oblique ridge is widespread among non-sauropodan sauropodomorphs and it is generally associated to the presence of a buccal emargination (e.g. Barrett, 2009; Barrett et al., 2005, 2007; Chapelle et al., 2019; Galton & Kermack, 2010; Pol & Powell, 2007; Prieto-Márquez & Norell, 2011; Schaeffer, 2024; Yates, 2003a; Zhang et al., 2020).

In lateral view, the dentary bifurcates caudally into two triangular processes that define a rostrally pointing, V-shaped posterior margin of the bone while diverging from each other (Figs. 3, 4), similarly to several non-sauropodan sauropodomorphs, e.g. *Pla. trossingensis* (Lallensack et al., 2021; Schaeffer, 2024) and *Col. brevis* (Apaldetti et al., 2014), but not *Arc. pereirabdalorum* (Yates et al., 2011) and *Mus. patagonicus* (Pol & Powell, 2007). The dorsocaudal process is the largest and it is dorsolaterally overlapped by the coronoid eminence of the surangular (Figs. 3, 4). Furthermore, it forms the rostrodorsal margin of the external mandibular fenestra. Differently, the caudoventral process is slightly shorter and ventrally concave and it overlaps the angular (Figs. 3, 4). It is not clear whether the latter process contributed to the rostroventral margin of the external mandibular fenestra given the fragmentary nature of the right process and the disarticulation degree of the left dentary. The medial surface of the dentary is characterized by a rostral, subcrescentic symphyseal articular surface from which a shallow Meckelian groove develops caudally, running longitudinally along the ventromedial margin of the bone (Fig. 11C, D).

Based on the right element, the dentary tooth row consists of at least 18 alveoli and a maximum of 20, with the last putative tooth position inserted at the level of the rostralmost contact with the surangular (Figs. 3, 4). Combining visual observations and the µCT output, a total of 8 and 17 in place teeth was detected respectively in the left and right dentary (Fig. 11C, D). A comparable tooth count is widespread among plateosaurian sauropodomorphs, e.g. *Iss. saaneq* (Beccari et al., 2021) and *Col. brevis* (Apaldetti et al., 2014), but it is highly exceeded in somatically mature individuals of *Pla. trossingensis* (30) (Lallensack et al., 2021; Schaeffer, 2024) and *Mas. carinatus* (26) (Gow et al., 1990; Sues et al., 2004).

The first alveolus is placed adjacent to the mandibular symphysis, but the first dentary tooth erupts dorsocaudally in a way that results caudally inset from the rostralmost margin of the dentary, leaving an edentulous space equal to the width of a tooth root in both lateral and medial views (Fig. 11C, D), similarly to most basal sauropodomorphs (e.g. Apaldetti et al., 2011, 2014; Galton & Kermack, 2010; Müller, 2020; Pol & Powell, 2007; Pretto et al., 2019; Rowe et al., 2011; Yates, 2007; Yates et al., 2011).

The first nine alveoli are oval shaped, being labiolingually compressed, and fully separated from each other by bony septa. The labial alveolar margin, which corresponds to the dorsolateral border of the dentary, is higher than the lingual one, entailing the tooth roots to be medially exposed, a common feature occurring in many other basal sauropodomorphs (Fig. 11B) (e.g. Barrett, 2009). Furthermore, the lingual alveolar margin is indented by the presence of thin, triangular interdental plates placed in between two adjacent teeth (Fig. 11C, D).

### Surangular

The surangulars are well preserved, despite being partially fractured close to their respective glenoid fossae. Furthermore, the left element, which has been ventrally displaced resulting in a semiarticulation with the surrounding bones, possesses a taphonomically deformed retroarticular process, being ventrally stretched at its base and thus displaying a stouter morphology compared to the right one (Fig. 4).

The surangular is a robustly built, rostrocaudally elongated bone that forms most of the caudolateral region of the mandibular ramus as well as the dorsal and caudal margins of the external mandibular fenestra (Figs. 3, 4). It contacts the dentary rostrodorsally, the angular ventrally, the prearticular ventromedially and the articular caudomedially.

Like almost all non-sauropodan sauropodomorphs (e.g. Pol & Powell, 2007,), the surangular is sigmoidal in shape, a condition reflected also along its dorsal margin, having the rostrodorsal portion markedly convex and elevated compared to the shallowly concave and low caudalmost region (Figs. 3, 4). Anteriorly, the bone develops as a medially expanded, rostroventrally oriented process that distally tapers dorsoventrally, overlapping the dorsocaudal process of the dentary and extending rostrally beyond the external mandibular fenestra, whereas it forms the dorsolaterally inflated coronoid eminence as it expands caudally like most of the non-sauropodan sauropodomorphs, but differing from the less developed morphologies of *Pan. caducus* (Galton & Kermack, 2010), *Mac. itaquii* (Müller, 2020), *Mel. readi* (Yates, 2007) and *Mus. patagonicus* (Pol & Powell, 2007). Furthermore, the ventral margin of this rostral flange defines the dorsal edge of the external mandibular fenestra. Noticeably, a sharp ridge runs along most of the dorsal margin of the surangular, being continuous with the prominent dorsolateral edge of the dentary, but faints, becoming dorsoventrally broader, while extending caudoventrally behind the mandibular opening, thus leading the caudal dorsomedial surface of the bone to face dorsolaterally as in *Pla. trossingensis* (Prieto-Márquez & Norell, 2011; Schaeffer, 2024).

Behind the anterior process, the surangular abruptly expands ventrally, defining a concave caudal margin of the external mandibular fenestra (Figs. 3, 4). A short indentation is present at the dorsocaudal corner of both the mandibular openings, however it is not clear whether it is a taphonomic artifact rather than a proper anatomical feature.

The lateral surface of the surangular is slightly convex, but possesses a shallow, rostroventrally placed notch for the reception of the angular, as visible in the left element (Fig. 4). Accordingly, the angular overlaps the surangular ventrally, defining a longitudinally extensive contact that reaches the level of the glenoid fossa. A rostrocaudally elongated, teardrop shaped foramen pierces the lateral surface of the bone in between the dorsal ridge and the jaw joint (Figs. 3, 4), comparably to many basal sauropodomorphs (e.g. Barrett, 2009; Martínez & Alcober, 2009; McPhee et al., 2019; Müller, 2020; Pol & Powell, 2007; Prieto-Márquez & Norell, 2011; Sereno et al., 2013; Sues et al., 2004; Zhang et al., 2020).

In lateral view, the surangular gets constricted dorsoventrally as it extends caudally, with the dorsal margin steeply sloping caudoventrally and forming a deeply concave, cup-shaped caudolateral embayment that corresponds to the surangular component of the glenoid fossa (Figs. 3, 4). Differently, in medial view, the surangular expands as a convex, caudomedially oriented lamina that wraps the rostral surface of the glenoid fossa, overlapping the articular and contacting the prearticular ventrally. Remarkably, the jaw joint is not aligned with the dorsal margin of the mandibular ramus as in *Pan. caducus* (Galton & Kermack, 2010; Yates, 2003b), *Rio. incertus* (Bonaparte & Pumares, 1995) and *Mas. carinatus* (Sues et al., 2004), but it is rather positioned well below the dentary tooth row (Figs. 3, 4) similarly to *Pla. trossingensis* (e.g. Schaeffer, 2024), *Col. brevis* (Apaldetti et al., 2014), *Luf. huenei* (Barrett et al., 2005) and *Mus. patagonicus* (Pol & Powell, 2007).

Behind the glenoid fossa, the surangular develops dorsocaudally as a mediolaterally thin, elongated triangular process that forms the lateral wall of the retroarticular process, which sutures medially with the lateral surface of the articular (Figs. 3, 4, 6). Visible in right lateral view, the retroarticular process is rather elongate and shallow, having a length that exceeds the mandibular depth at the level of the jaw joint (Fig. 3), analogously to several non-sauropodan sauropodomorphs, e.g. *Pla. trossingensis* (Schaeffer, 2024), *Col. brevis* (Apaldetti et al., 2014), *Luf. huenei* (Barrett et al., 2005), *Jin. xinwaensis* (Zhang et al., 2020), but not *Mas. carinatus* (Barrett & Yates, 2005). Accordingly, the retroarticular process extends beyond the dorsocaudal corner of the skull.

### Angular

The left angular is disarticulated and medially dislocated in between the two hemimandibles, having its lateral surface facing ventrally and partially covered by the isolated left splenial (Fig. 7). On the other hand, the right bone is perfectly preserved and in proper anatomical connection with the other mandibular elements (Fig. 3).

The angular is an elongate, mediolaterally thin strap-like bone that forms the caudal third of the ventral region of the mandibular ramus, contacting the dentary rostrally, the surangular dorsocaudally and the prearticular ventromedially. Remarkably, the right main body is 3.6 times as long as high, being proportionately shorter than the holotype of *Pla. trossingensis* (6.5; SMNS 13200) (Schaeffer, 2024).

The angular is sigmoidal in lateral view, being dorsoventrally constricted at the level of the coronoid eminence of the surangular and defining a deeply concave dorsal margin anteriorly, which corresponds to the ventral margin of the external mandibular fenestra (Fig. 3). A comparable morphology is present in several non-massopodan sauropodomorphs (e.g. Apaldetti et al., 2014; Marsh & Rowe, 2018; Martínez & Alcober, 2009; McPhee et al., 2019; Müller, 2020; Schaeffer, 2024; Sereno et al., 2013), whereas more derived taxa, especially some massospondylids and sauropodiforms, are characterized by a robustly built angular that keeps a constant depth throughout its entire length and does not account for any marked dorsoventral inflection along its dorsal margin (e.g. Sues et al., 2004; Barrett et al., 2005; Yates, 2007; Barrett, 2009; Martínez, 2009; Chapelle et al., 2019; Zhang et al., 2020; Whang et al., 2020).

In detail, the main body increases in height both rostrally, where it is overlaid by the caudoventral process of the dentary, and caudally, behind the external mandibular fenestra where it overlaps the lateral surface of the surangular (Figs. 3, 7). Furthermore, posteriorly to the caudal convexity, the angular tapers dorsoventrally as it extends towards the ventral base of the retroarticular process, terminating at the level of the glenoid fossa (Fig. 3).

The ventral surface is convex in ventral view, following the curvature of the surangular and folding medially, but has a straight margin in lateral view (Fig. 7). The caudalmost portion of the angular establishes a contact with the prearticular ventromedially.

### Splenial

The splenial is represented in SMF 13.5.37 by an isolated left element visible in ventral view, having its medial surface exposed ventrally (Fig. 7). The splenial is an elongate, subrectangular bony lamina that articulates laterally with the lingual side of the dentary.

The ventral margin is wavy and slopes rostroventrally by 15° compared to its caudal region, almost matching the rostral inclination of the dentary (see “Dentary” section), whereas it is dorsocaudally pointing at its distal end (Fig. 7). Furthermore, the splenial folds laterally along its ventral surface, wrapping around the mandibular ramus. On the other hand, the dorsal margin seems to be incomplete, thus making it impossible to determine whether the splenial had the anterior and the caudal margins bifurcated, like in *Bur. schultzi* (Müller et al., 2018a), *Pla. trossingensis* (Prieto-Márquez & Norell, 2011; Schaeffer, 2024), *Col. brevis* (Apaldetti et al., 2014) and *Ade. mognai* (Martínez, 2009). The medial surface of the splenial is flat and featureless.

### Prearticular

The caudalmost surface is the only region exposed in both prearticulars, visible in left medial view. Differently from the right bone that is in proper anatomical position, the left element is fragmented and taphonomically displaced rostroventrally, being disarticulated and having its medial surface facing downwards (Figs. 6, 7).

The prearticular consists of a mediolaterally thin lamina that gradually decreases in height as it extends rostrally, contacting the angular along its ventral margin and the medial expansion of the surangular dorsally. Similarly to most basal sauropodomorphs (e.g. Apaldetti et al., 2014; Barrett et al., 2005, 2007; Schaeffer, 2024), the prearticular expands dorsoventrally at the level of the glenoid fossa as a convex, irregularly outlined lamina that envelops laterally the medial process of the articular, resulting in having a dorsomedial orientation (Figs. 6, 7). Immediately behind the jaw joint, the dorsal margin slopes caudoventrally, tapering caudally to a point and reaching the base of the articular component of the retroarticular process. Noticeably, the prearticular does not contribute to the retroarticular process, differently from some Carnian sauropodomorphs like *Eor. lunensis* (Sereno et al., 2013), *Bur. schultzi* (Müller et al., 2018a) and *Panphagia protos* Martínez & Alcober, 2009.

### Articular

The left articular is fragmentary, missing the dorsalmost portion of the retroarticular process. Furthermore, it is slightly disarticulated rostrally and rotated by almost 90° along its main axis, leading its ventral margin to be ventromedially oriented (Figs. 4, 6). On the other hand, the right element is perfectly preserved and articulated with the surrounding mandibular bones (Fig. 3).

The articular forms the caudomedial region of the mandibular ramus, contributing to the glenoid fossa rostrally and the retroarticular process caudally. It contacts the surangular rostrally and laterally, the prearticular ventromedially and the quadrate dorsally, forming the craniomandibular joint with the latter.

The articular is P-shaped due to the presence of a medially expanding, rostrocaudally convex pyramidal process at its anteriormost region (Figs. 6, 7), as in many basal sauropodomorphs (e.g. Apaldetti et al., 2014; Beccari et al., 2021; Prieto-Márquez & Norell, 2011; Schaeffer, 2024; Sues et al., 2004; Wang et al., 2020; Yates, 2007), but not *Mac. itaquii*, in which it is rather reduced (Müller, 2020). In dorsal view, this process contributes to the medial portion of the glenoid fossa, wrapping around the entocondyle of the quadrate and being rostrally overlapped by the medial expansion of the surangular. In medial view, the internal margin of the jaw joint is more ventrally located than the external one defined by the surangular, reflecting the extension disparity of the quadrate condyles.

A concave, dorsocaudally facing depression is present on the rear dorsal surface of the pyramidal process and it is medially marked by the glenoid margin (Fig. 6), as in *Pla. trossingensis* (Prieto-Márquez & Norell, 2011) and possibly *Mas. carinatus* (Barrett & Yates, 2005).

In medial view, the dorsal margin of the articular is deeply concave at the posterior end of the glenoid fossa, whereas, behind the latter, it expands dorsally, reaching the same height of the caudalmost portion of the surangular.

The caudal region of the articular consists of a mediolaterally thin, vertically oriented subtriangular lamina that tapers dorsoventrally to a blunt end as it extends posteriorly and that corresponds to the articular component of the retroarticular process, which contacts laterally the surangular component (Figs. 3, 4, 6, 7).

Noticeably, the dorsal surface of the retroarticular process is markedly depressed and U-shaped in caudal view, having a longitudinal trough running along the entire extent of the process and rostrally opening into the glenoid fossa, similarly to several basal sauropodomorphs, like *Eor. lunensis* (Sereno et al., 2013), *Pla. trossingensis* (Prieto-Márquez & Norell, 2011; Schaeffer, 2024), *Mac. itaquii* (Müller, 2020), *Mas. carinatus* (Barrett & Yates, 2005) and *Luf. huenei* (Barrett et al., 2005). This groove has been referred as the insertion point of the *M. depressor mandibulae* (e.g. Dilkes et al., 2012; Holliday, 2009). On the other hand, the ventral margin of the retroarticular process forms a sharp, slightly convex ridge.

### Hyoid

A rod-like, cylindrical fragment of the hyoid apparatus, precisely belonging to the first third of the right ceratobranchial, is found beneath the central region of the right mandibular ramus (Figs. 3, 7). As in other basal sauropodomorphs (e.g. Müller, 2020), the rostral end reaches the anterior margin of the antorbital fenestra and seems to be dorsoventrally expanded. Nonetheless, this latter feature cannot be certainly confirmed due to the presence of encasing matrix. Most of the caudal portion of the shaft is now missing, however, based on pictures of the specimen taken before the mechanical preparation, it appears that the right ceratobranchial accounted for a more complete section that was at least three times longer than the preserved fragment and ventrally bowed.

### Dentition

**Fig. 12:**
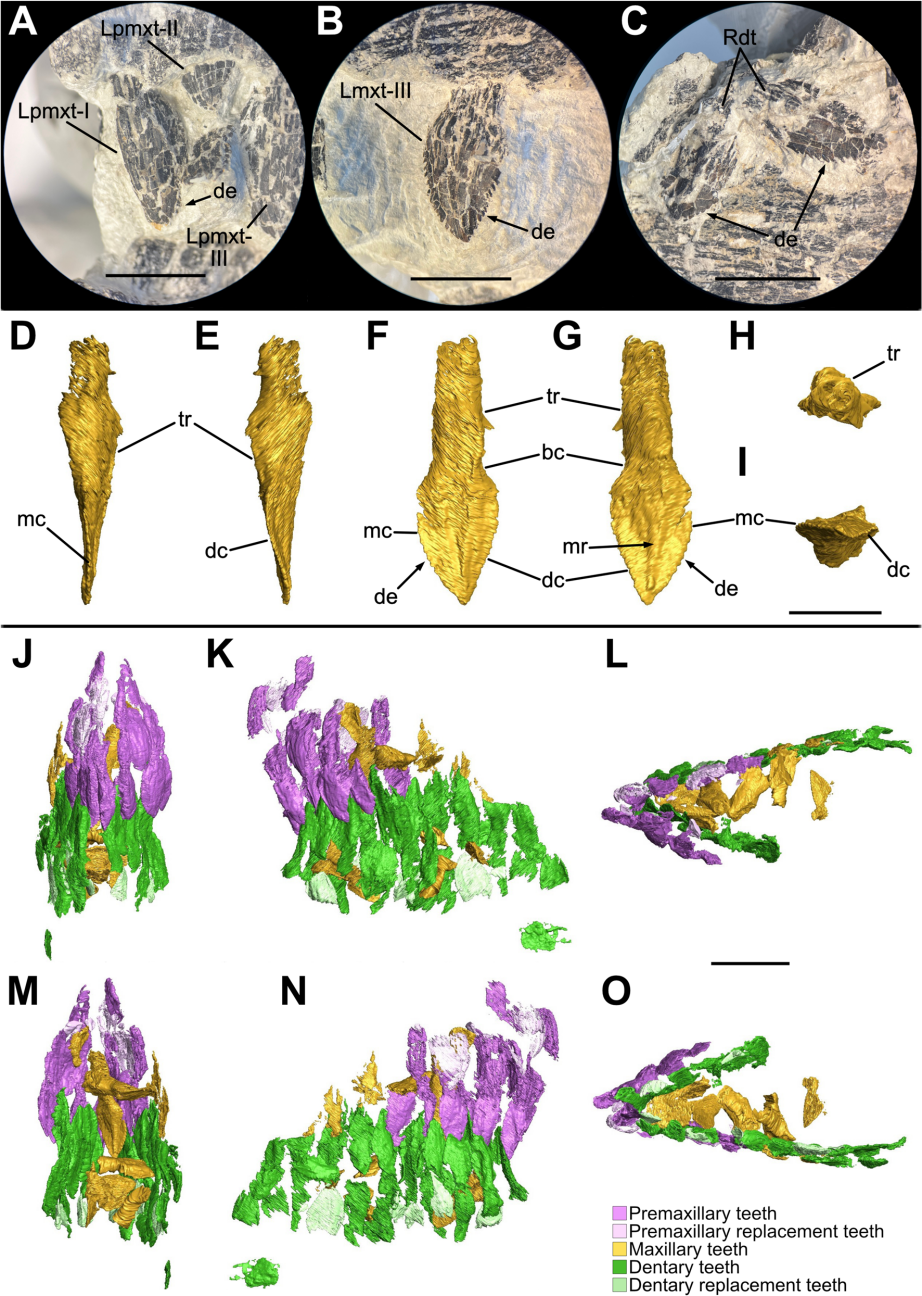
Overview of the dentition of SMF 13.5.37. **A** First, second and third left premaxillary teeth. **B** Third left maxillary tooth. **C)** Isolated right dentary teeth. Isolated left maxillary tooth in mesial (**D**), distal (**E**), labial (**F**), lingual (**G**), apical (**H**) and basal (**I**) views. Three-dimensional rendering of the segmented dentition within the snout of SMF 13.5.37: rostral (**J**), left lateral (**K**), dorsal (**L**), caudal (**M**), right lateral (**N**) and ventral (**O**) views. Scale bar for **A**–**I** equals 1 cm. Scale bar for **J**–**O** equals 3 cm

The dentition of SMF 13.5.37 consists per side of an upper tooth row that bears approximately 25 alveoli, 4 and 21 respectively located in the premaxilla and the maxilla, and a lower tooth row completely encased within the dentary that accounts for at least 18 alveoli (Figs. 3, 4, 12J–O). In detail, all the premaxillary teeth are preserved in both elements, whereas only 5 and 12 are still placed within the left and right maxilla. The right dentary preserves 17 teeth, differently from the right one, which has only 8 laterally exposed. Additionally, 4 isolated tooth crowns, two of which possibly belonging to the dentary teeth, and several splinters are found scattered in proximity to the rostral half of the lateral surface of the right dentary, mostly close to its dorsal and ventral margins (Figs. 3, 4, 12J–O). Noticeably, the µCT analysis unveiled the presence of several replacement teeth as well as 9 isolated teeth imbricated within the premaxillary block, half of which are either partially or fully-rooted, and almost all represent elements from the maxillary tooth rows (Fig. 12J–O). Despite the elevated number of preserved elements, all teeth are highly fragmented, showing longitudinal fractures or even missing patches on the enamel as the result of both sediment compaction and long-lasting exposure prior to burial (Fig. 12A–C).

The overall morphology of the teeth is consistent with that of non-sauropodan sauropodomorphs, being lanceolate, apicobasally higher than mesiodistally wide, labiolingually compressed and with a constriction at the base of the crown, which marks the transition to the root (Fig. 12) (e.g. Apaldetti et al., 2014; Beccari et al., 2021; Becerra et al., 2017; Chapelle et al., 2019; Marsh & Rowe, 2018; Martínez, 2009; Prieto-Márquez & Norell, 2011; Sues et al., 2004). Nonetheless, the dentition of SMF 13.5.3 is moderately heterodont as in several massopodans, especially massospondylids (Apaldetti et al., 2011; Knoll, 2010; Marsh & Rowe, 2018; Sues et al., 2004), and differs from the rather homodont condition of non-massopodan sauropodomorphs, as in Carnian taxa (e.g. Müller et al., 2018a), plateosaurids (Beccari et al., 2021; Prieto-Márquez & Norell, 2011; Schaeffer, 2024) and unaysaurids (McPhee et al., 2020; Müller, 2020).

The premaxillary teeth are subequal in height and possess an asymmetric outline in lateral view, with the mesial margin medially curved and the distal margin caudally oriented and more expanded dorsocaudally (Figs. 8C, D, 12A), as in *Pla. trossingensis* (Prieto-Márquez & Norell, 2011) and *Mas. carinatus* (Barrett & Yates, 2005). Particularly, the degree of curvature of the mesial carina gradually changes through the tooth series, being highly exaggerated in the first and second premaxillary teeth and becoming more rostromedially pointing in the third and fourth ones (Figs. 8F, 12A). Accordingly, the orientation disparity of the mesial carinae is reflected in the general arrangement of the tooth crowns, which are obliquely positioned in the tooth row resulting in a stepped, U-shaped outline in ventral view (Fig. 8F). As a further consequence, the mesiodistal width caudally increases through the tooth series, leading the distal carinae of the third and fourth premaxillary tooth crowns to overlap the labial surface of the mesial carinae of their respective consecutive teeth (Fig. 8C, D), like other plateosaurian sauropodomorphs, e.g. *Pla. trossingensis* (Schaeffer, 2024), *Sar. aurifontanalis* (Marsh & Rowe, 2018) and *Mas. carinatus* (Chapelle & Choiniere, 2018).

The premaxillary teeth reach their maximum mesiodistal width at the basal third of the crown height, having a slenderness index (SI, *sensu* Upchurch, 1998) that decreases along the tooth row, from 2.45 (first position) to 1.95 (third position). Remarkably, a lower SI is recorded for both fully developed replacement teeth, like the second left tooth (1.6), and newly erupted teeth, as the left third tooth (1.5), however it might have been exaggerated due to taphonomic deformation.

In lateral view, the first and second premaxillary teeth are characterized by an inflated, apicobasal eminence that runs through the entire height of the tooth crowns at the level of the anterior margin of the roots (Figs. 8, 12A). This swollen convexity leads the teeth to have a halfmoon-shaped cross section and to be labiolingually thicker than the more compressed and mesiodistally broader morphologies of the third and fourth premaxillary teeth.

All the premaxillary teeth have a convex labial surface and a concave lingual surface, together terminating ventrally into a subtriangular apex that is slightly medially oriented and not caudally recurved (Fig. 8). In detail, the labial surface is smooth and unornamented, despite the presence of low fluting that is attributable to the taphonomic fragmentation. On the other hand, the lingual surface is always characterized by a pronounced, median ridge that extends apicobasally from the base of the tooth crown to its tip and that divides the surfaces into two marked concavities, respectively placed mesial and distal to the ridge itself (Fig. 8B), similarly only to *Mas. kaalae* (Barrett, 2009) and *Mel. readi* (Yates, 2007). Remarkably, this latter feature is present also in the replacement teeth, indicating that it is genuine and not a taphonomic artifact.

Both the mesial and distal carinae are sharp and coarsely serrated along their apical two-thirds, bearing subconical denticles (1–2 per mm) that are upwardly directed at roughly 45° with respect to the main axis of the tooth (Fig. 8). Denticulated carinae are also present in the replacement teeth. This condition is similar to *Pla. trossingensis* (e.g. Schaeffer, 2024) and *Mas. carinatus* (e.g. Sues et al., 2004), but differs from several sauropodomorphs that lack denticles on either one of the two or both premaxillary carinae, like *Bur. schultzi* (Müller et al., 2018a), *Pan. caducus* (Galton & Kermack, 2010), *Iss. saaneq* (Beccari et al., 2021), *Mac. itaquii* (Müller, 2020), *Ley. marayensis* (Apaldetti et al., 2011), *Yun. huangi* (Barrett et al., 2007), *Mel. readi* (Yates, 2007) and *Mus. patagonicus* (Pol & Powell, 2007).

Each tooth is deeply implanted in the premaxillary main body with a long, dorsomedially arched root that follows the curvature of the bone itself and that forms the 50% of the entire tooth height. In detail, the first pair of premaxillary tooth roots is cylindrical and more robust compared to the other ones, which are rather labiolingually compressed and subelliptical in cross section (Fig. 12N).

Remarkably, the tooth implantation of the premaxillary teeth is not homogeneous. Specifically, the first and second teeth are set perpendicularly to the premaxillary tooth row, thus resulting in being caudoventrally oriented in lateral view (Figs. 8C, D, 12L, M). On the other hand, the third and fourth teeth are slightly procumbent with respect to the alveolar margin and appear to have a vertical orientation in lateral view (Figs. 8C, D, 12L, M).

Four unerupted replacement teeth are found within the first and third pairs of alveoli. In detail, the premaxillary replacement teeth embedded within the left tooth row appear to be at a germinal stage, with only the ventralmost portion of the tooth crown developed, which is rather labiolingually inflated compared to the erupted equivalent and with already denticulated carinae. Furthermore, both of them are placed dorsomedial to the descending, exposed tooth, a pattern shared with several basal sauropodomorphs (e.g. Beccari et al., 2021; Chapelle & Choiniere, 2018). On the other hand, the replacement teeth in the right premaxilla have already fully developed crowns that are inset dorsal to the roots of the respective erupted teeth.

The maxillary teeth are morphologically comparable to the third and fourth premaxillary teeth, even though they differ in being labiolingually thinner with a subelliptical cross section and mesiodistally wider than the latter, as best visible in the left upper tooth row (Fig. 12B, D–I). Embedded in between the jaws, an isolated, though perfectly preserved, fully-rooted tooth possesses the aforementioned features, thus can be referred to a left maxillary tooth, which likely slipped out from either the fourth or the fifth alveolus (Fig. 12D–I).

In both labial and lingual views, the maxillary tooth crowns are spear-tip shaped, not caudally recurved and highly asymmetric, having a markedly convex mesial margin that is more mesiodistally inflated than the distal one (Fig. 12B, F, G). The maximum breadth is reached at the basal third and the SI is not gradually decreasing throughout the tooth row as in the premaxillary teeth, but rather fluctuates in values comprised between 1.4 and 1.6, indicating that the height-to-width ratio is mostly retained in the entire series even though the individual crowns get drastically reduced in size caudally like many basal sauropodomorphs (Fig. 3) (e.g. Barrett et al., 2005, 2007; Martínez, 2009; Müller, 2020; Schaeffer, 2024).

In rostral and caudal views, the mesial margin is slightly convex and rostromedially oriented (Fig. 12D), contrasting the medially bent one of the premaxillary teeth, whereas the distal margin points caudolaterally and is sigmoidal (Fig. 12E), entailing that the crowns were originally imbricated displaying an en-echelon arrangement that widely occurs in several non-sauropodan sauropodomorphs, e.g. *U. tolentinoi* (McPhee et al., 2019), *Ngw. intloko* (Chappelle et al., 2019) and *Luf. huenei* (Barrett et al., 2005). Accordingly, the tooth crowns are obliquely set within the maxillary tooth row in ventral view. Nonetheless, an overlapping pattern cannot be properly evaluated due to the paucity of well-preserved elements in series. The labial surface is flat-to-convex and often featureless, even though some low striations alternated with shallow grooves are detected in the best-preserved teeth (Fig. 12F), whereas the lingual surface is slightly concave and divided in two depressed areas due to the presence of an apicobasal median ridge that is analogous to the one in the premaxillary teeth (Fig. 12G).

Both mesial and distal margins are characterized by sharp carinae that show the same denticulation pattern as the premaxillary teeth, extending upwardly from the basal third (Fig. 12D, I) as in non-massopodan sauropodomorphs, like *Pla. trossingensis* (e.g. Schaeffer, 2024), *Iss. saaneq* (Beccari et al., 2021), *U. tolentinoi* (McPhee et al., 2019) and contrasting more derived taxa, like non-sauropodiform massopodans and sauropodiforms in which it is restricted to the apical third (e.g. Apaldetti et al., 2014; Barrett & Yates, 2005; Chapelle et al., 2019; Pol & Powell, 2007).

The maxillary teeth are firmly inserted within the tooth row thanks to hollow, deep, cylindrical roots that are the most robust in the entire dentition (Fig. 12H). Even though the main axis of each element is vertical, the maxillary teeth are obliquely inset compared to the alveolar margin in lateral view, showing a weakly procumbent orientation that is parallel to that of the last two premaxillary teeth (Figs. 3, 4, 12B), but that becomes more perpendicular as the upper tooth row extends caudally (Fig. 3).

The dentary teeth are leaf-shaped with a pronounced distal margin, resulting in a more symmetric outline than the maxillary teeth (Fig. 12C) that accounts for SI values ranging between 2.2 and 1.8 throughout the lower series. Furthermore, they differ from the upper jaw elements due to straighter, almost completely flat tooth crowns that are considerably thinner labiolingually, as noticeable in cross section and in both rostral and caudal views (Fig. 11). Nonetheless, the dentary teeth are similar to some extent to the maxillary ones as they show the same denticulation pattern, a more concave lingual surface in the posterior teeth and a gradual size reduction towards the caudal positions (Figs. 3, 11, 12). On the other hand, the crown ornamentation and the tooth replacement pattern are identical to those of the premaxillary teeth.

Remarkably, the first three pairs of dentary teeth are comparable to the first two premaxillary ones, sharing a mesial carina that is strongly angled medially and whose curvature gradually weakens in the posterior teeth (Fig. 11A, B, E). Accordingly, the rostralmost dentary tooth is characterized by a C-shaped cross section, contrasting the broader ones of the following teeth (Fig. 11E).

In lateral view, the dentary teeth are closely packed and juxtaposed in an overlapping en-echelon arrangement (Figs. 3, 11C, D), as in several basal sauropodomorphs (e.g. Apaldetti et al., 2011; Barrett, 2009; Gow et al., 1990; Martínez, 2009). The imbricated pattern is also defined by the tilted disposition of the dentary teeth within the lower tooth row, which have the sagittal axis rostromedially oriented (Fig. 11). In dorsal view, the two conjoined mandibular dental series are displayed in a V-shaped arrangement that is rostrally narrower than the broader outline of the premaxillary ones, implicating that the lower tooth rows interlocked medially with the upper ones as the mandible clenched (Figs. 8F, 11E, 12J–O). As further evidence, the first two dentary teeth are weakly concave apicobasally and dorsocaudally oriented with respect to the mandibular alveolar margin, inducing their labial surfaces to match the lingual concavities of the respective premaxillary teeth and to rostromedially slide on them during the jaw motion (Figs. 12J–O, S2A, C, D).

### Ontogenetic status

The specimen SMF 13.5.37 displays fully-closed cranial sutures, especially in the neurocranial region where even a certain degree of co-ossification occurs, as in the completely fused exoccipital-opisthotic complex that forms the otoccipital. Even though the cranial sutural fusion has often been used as a proxy for somatic maturity in non-avian dinosaurs (e.g. Bakker & Williams, 1988; Sampson et al., 1997), Bailleul et al. (2016) argued about its reliability and stated that progressive fusion throughout ontogeny should be cautiously employed (e.g. Galton & Kermack, 2010).

The premaxilla of SMF 13.5.37 shares an edentulous gap between its rostral tip and the first pair of premaxillary teeth (Fig. 8) with somatically mature individuals of *Pla. trossingensis*, differently from immature or not fully-grown specimens of the latter taxon and *Iss. saaneq* (Beccari et al., 2021). According to Lallensack et al. (2021), this craniodental feature might be considered as an indicator of an advanced ontogenetic stage. In light of this, the skull SMF 13.5.37 is tentatively referred herein as belonging to a somatically mature individual, pending a proper histological analysis on the associated postcranial material that could confirm this ontogenetic status.

## Phylogenetic analysis

The heuristic search yielded 26,000 MTPs (most parsimonious trees) of 1689 steps each (consistency index CI = 0.289, retention index RI = 0.661). The exploration of the strict consensus tree (CI = 0.251, RI = 0.588) found several unresolved polytomies, the largest of which comprises several non-plateosaurian sauropodomorphs, non-massospondylid massopodans, massospondylids as well as SMF 13.5.37 (Fig. S1). In all the MTPs, SMF 13.5.37 unstably clusters within the clade formed by Plateosauridae and Unaysauridae, precisely as the basalmost member of either the clade itself or one of the two families.

**Fig. 13:**
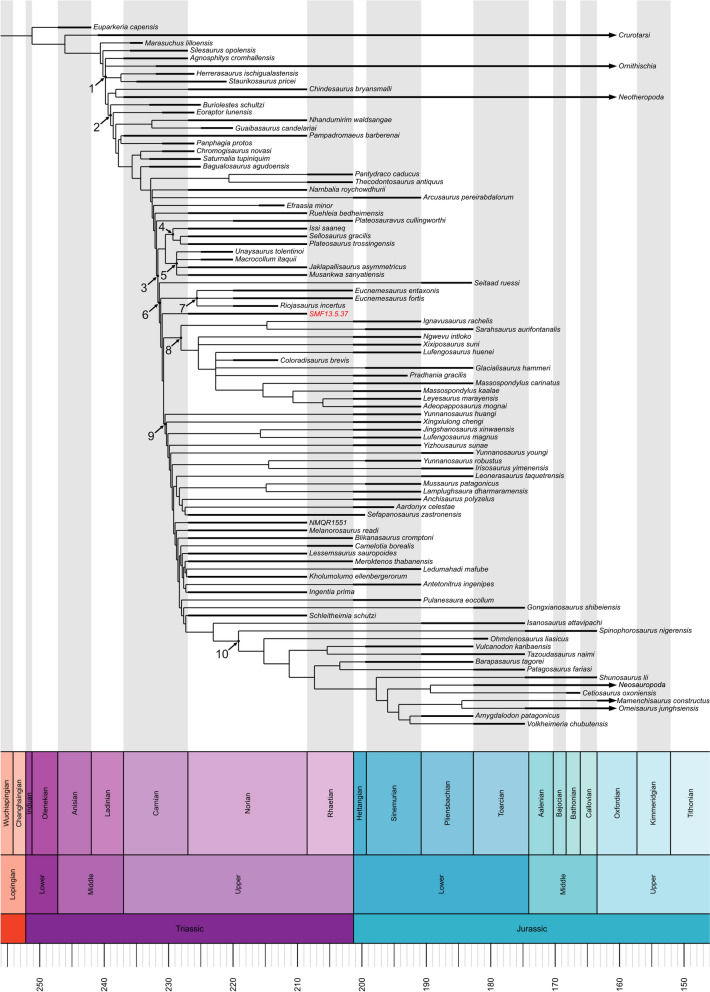
Strict consensus tree recovered from the heuristic search under implied weights (K = 12) of the modified matrix from Ezcurra et al. (2024). Node numbers: 1, Dinosauria; 2, Sauropodomorpha; 3, Plateosauria; 4, Plateosauridae; 5, Unaysauridae; 6, Massopoda; 7, Riojasauridae; 8, Massospondylidae; 9, Sauropodiformes; 10, Sauropoda. Tree calibration and plotting used Paleotree (Bapst, 2012) and Strap R (Bell & Lloyd, 2015) packages

The heuristic search under implied weighting (K = 12) recovered 45 MTPs with a score of 72.88757 (CI = 0.289; RI = 0.660), from which a well-resolved strict consensus tree (CI = 0.284; RI = 0.653) is returned with minor polytomies (Fig. 13) (e.g. at the base of Dinosauria, within Unaysauridae, Riojasauridae and Massospondylidae). The overall topology is congruent to the phylogenetic results of Ezcurra et al. (2024) and similar to those of Beccari et al. (2021) and Apaldetti et al. (2021), but slightly differs from the analysis of Müller (2020) and Pol et al. (2021) due to the position of both Unaysauridae, which nests as the earliest branching clade of Massopoda in the latter and not as the sister group of Plateosauridae like in the presented results, and some unstable “wildcards”, e.g. *Pradhania gracilis* Kutty et al., 2007 and *Seitaad ruessi* Sertich and Loewen, 2010. Furthermore, *Mus. sanyatiensis* resulted as a member of Unaysauridae, contrasting the basalmost massopodan position recovered in Barrett et al. (2024). SMF 13.5.37 is placed as sister taxon of the clade formed by Massospondylidae + Sauropodiformes along the third massopodan branch, diverging after Riojasauridae (Fig. 13).

**Fig. 14:**
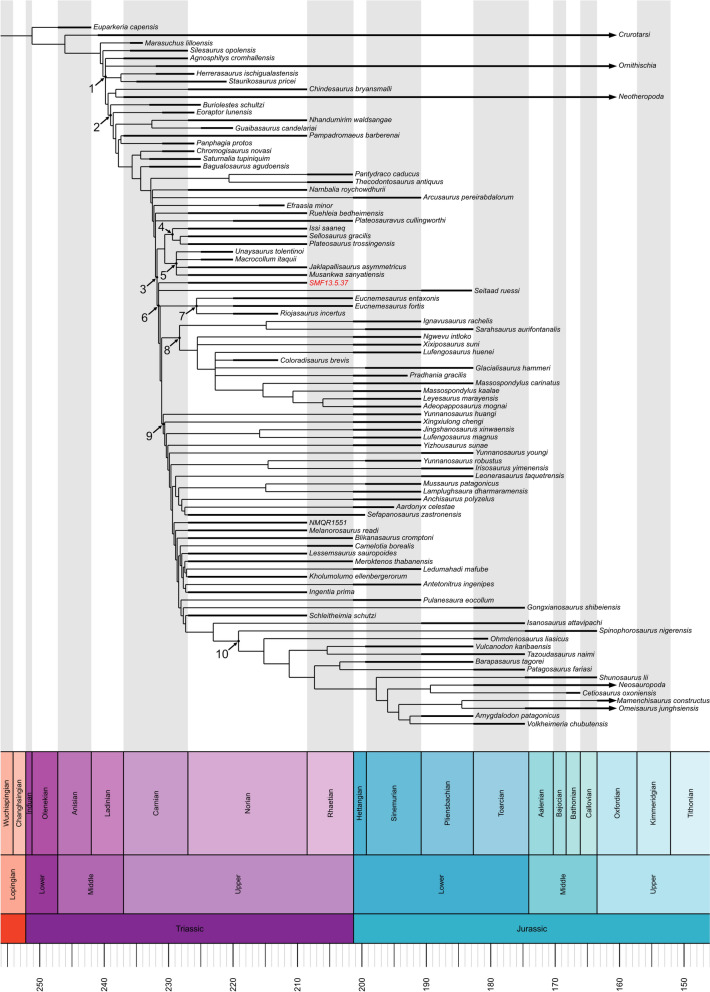
Strict consensus tree recovered from the heuristic search under implied weights (K = 12) of the modified matrix from Ezcurra et al. (2024) with characters 13 and 14 omitted. Node numbers: 1, Dinosauria; 2, Sauropodomorpha; 3, Plateosauria; 4, Plateosauridae; 5, Unaysauridae; 6, Massopoda; 7, Riojasauridae; 8, Massospondylidae; 9, Sauropodiformes; 10, Sauropoda. Tree calibration and plotting used Paleotree (Bapst, 2012) and Strap R (Bell & Lloyd, 2015) packages

Given that the massopodan affinity of SMF 13.5.37 is supported by a single unambiguous synapomorphy [see Supplementary material, “Ezcurra et al. (2024): heuristic search under implied weights (K = 12)”] related to the subnarial foramen, which is not properly preserved in the specimen herein described (see “Premaxilla” section), a second round of phylogenetic analysis is conducted employing the same tree search methodology and scoring all characters based on the aforementioned morphological feature (i.e. characters 13 and 14) as missing (“?”) in order to avoid a possible interpretative bias. The heuristic search recovered 27,000 MTPs of 1688 steps each (CI = 0.290; RI = 0.662), the strict consensus (CI = 0.250; RI = 0.586) of which resulted in having the same topology as the former analysis under equal weighting (Fig. S1). The phylogenetic analysis using implied weights (K = 12) produced 90 MTPs with a score of 72.84345 (CI = 0.289; RI = 0.660), whose consensus tree (CI = 0.284; RI = 0.653) is almost topologically congruent with the previous results, but remarkably differs as it places SMF 13.5.37 in a small polytomy with *Sei. ruessi* along the basalmost massopodan branch, thus recovering the former as the earliest-diverging member of Massopoda given the Pliensbachian age of the latter (Fig. 14). Differently from the first round of analysis, SMF 13.5.37 has an affinity to Massopoda that is supported by three unambiguous synapomorphies (see Supplementary material, “Ezcurra et al. (2024): heuristic search under implied weights (K = 12) with characters 13 and 14 omitted”).

## Discussion

### Mosaic craniomandibular anatomy of SMF 13.5.37

Based on the presented phylogenetic analyses, the specimen SMF 13.5.37 belongs to a new basal massopodan sauropodomorph from the Late Triassic, namely the first non-*Plateosaurus* sauropodomorph from the Canton Aargau, that showcases a mosaic character suite that couples plesiomorphic states with more derived cranial features typical of massopodan taxa.

In detail, SMF 13.5.37 shares several craniomandibular features with non-sauropodan plateosaurians, such as: a dorsal profile of the snout with a depression behind the naris (except the plateosaurid *Iss. saaneq*); the dorsal margin of the postorbital with a distinct embayment between the rostral and the dorsocaudal processes (except the plateosaurids *Pla. trossingensis* and *Sel. gracilis*, the massospondylids *Ignavusaurus rachelis* Knoll, 2010 and *Sar. aurifontanalis* and the non-sauropodan sauropodiform *X. chengi*); a divergence angle between the jugal and squamosal rami of the quadratojugal close to 90° (except *Pla. trossingensis* and several massospondylids, like *Col. brevis* and *Luf. huenei*); four premaxillary teeth in each premaxilla (except for plateosaurids).

Differently, cranial traits typical of non-sauropodiform plateosaurians present in SMF 13.5.37 are: a web of bone spanning at the junction between the anterior and the ventral rami of the lacrimal that obscures the dorsocaudal corner of the antorbital fossa; the jugal contribution to the definition of the ventral margin of the antorbital fenestra (except for derived massospondylids, like *Mas. carinatus*, *Ley. marayensis* and *Ade. mognai*); the supraoccipital plate strongly sloping forward so that the tip lies at the level of the basipterygoid processes (except the basal massopodan *Rio. incertus* and the massospondylids *Mas. carinatus* and *Ade. mognai*).

On the other hand, SMF 13.5.37 resembles non-massopodan plateosaurians in possessing: the nasal extensively overhanging so that it obscures the dorsal contact between the maxilla and the lacrimal in lateral view (present also in the massospondylid *Mas. carinatus*); the ascending ramus of the maxilla tapering dorsally in lateral view (present also in the basal massopodan *Rio. incertus* and the massospondylids *Col. brevis* and *Ley. marayensis*); the contribution of the maxillary ascending ramus to the antorbital fossa being deeply impressed and delimited by a sharp, scarp-like rim (present also in the non-sauropodan sauropodiform *Aar. celestae*); a large maxillary lamina forming the antorbital fossa that occupies more than 25% of the rostrocaudal length of the antorbital fenestra, with a straight to gently concave caudal margin (except for the massospondylid *Col. brevis* and the non-sauropodan sauropodiforms *Aar. celestae* and *Mel. readi*); the ratio of the minimum depth of the jugal below the orbit to the distance between the anterior end of the jugal and the rostroventral corner of the infratemporal fenestra is less than 0.2 (except *Pla. trossingensis*, but present in the basal massopodan *Rio. incertus*); the denticulation pattern consisting of carinae that are serrated along most of the length of the tooth crown and not restricted to upper half of it.

Remarkably, SMF 13.5.37 exhibits some morphological characters in common with non-sauropodan massopodans, such as: the caudolateral process of the premaxilla and the rostroventral process of the nasal being briefly separated by the maxilla (present also in the plateosaurid *Iss. saaneq*); a slot-shaped subnarial foramen (present also in the unaysaurid *Mac. itaquii*); the length of the antorbital fossa that does not exceed the orbital one; an extension of the antorbital fossa onto the ventral corner of the lacrimal (present also in the plateosaurids *Pla. trossingensis* and *Sel. gracilis* and the unaysaurid *Mac. itaquii*); the rostral margin of the infratemporal fenestra extending under the rear half of the orbit (except the massospondylid *Col. brevis* and the non-sauropodan sauropodiform *X. chengi*); the medial margin of the supratemporal fossa being simple and smoothly curving; the symphyseal end of the dentary being in line with the long axis of the bone itself (except some derived massospondylids that are characterized by a distal rostroventral curvature).

Moreover, features of SMF 13.5.37 shared with non-sauropod sauropodiforms include: the orbital margin formed by the lacrimal being erect and close to vertical, similarly to only few taxa, namely *Jin. xinwaensis*, *Yizhousaurus sunae* Zhang et al., 2018 and *Yunnanosaurus robustus* Young, 1951; the dentary having a height: length ratio that is greater than 0.2 (present also in the massospondylid *Ngw*. *intloko*); a stout, triangular, medial process of the articular present behind the glenoid fossa (present also in the massospondylid *Col. brevis*).

Finally, SMF 13.5.37 possesses some specific craniomandibular features that are recorded also in other non-sauropod plateosaurians, but without being distinctive of specific clades, such as: the prefrontal possessing a maximum transverse width that is 25% of the skull width at the same level, comparably to the plateosaurid *Pla. trossingensis*, the massospondylids *Sar. aurifontanalis* and *Col. brevis* and the non-sauropod sauropodiforms *Aar. celestae* and *Mel. readi*; the floor of the braincase being bent with the basipterygoid processes below the level of the basioccipital condyle and the basal tuberae, similarly to the plateosaurids *Pla. trossingensis* and *Sel. gracilis* and the massospondylids *Luf. huenei* and *Col. brevis*; the jugal ramus of the quadratojugal being shorter than the squamosal ramus, like in the plateosaurid *Pla. trossingensis*; the paroccipital processes being horizontal-to-dorsolaterally oriented, as in the massospondylids *Sar. aurifontanalis* and *Ngw. intloko*; the maxillary tooth crowns being procumbent, like in the massospondylids *Ley. marayensis* and *Ade. mognai*; the frontal contributing to the supratemporal fenestra, a condition shared only with the non-sauropod sauropodiform *X. chengi*.

### Macroevolutionary implications of SMF 13.5.37

SMF 13.5.37 increases the continental European faunal diversity of the mid-latest Norian non-sauropodan sauropodomorphs, representing the fourth officially described Swiss taxon along with the plateosaurid *Pla. trossingensis*, the enigmatic *Gre. ingens* (often regarded as *“Plateosaurus” ingens*, e.g. Yates, 2007; McPhee et al., 2015) and the sauropodiform *Sch. schutzi* (Rauhut et al., 2020). As discussed by Rauhut et al. (2020), additional sauropodomorph material found at Hallau-Schwärzibuck (Canton Schaffhausen) might represent another large non-sauropodan sauropodomorph taxon, although not yet recognized due to the fragmentary nature of the referred specimens.

The establishment of the new taxon is validly supported by a set of diagnostic cranial features that does not overlap with any other known sauropodomorph (see Supplementary material, “supporting synapomorphies of Sauropodomorpha nodes present in SMF 13.5.37 and unique combinations of characters”) and the taxonomic affinities of SMF 13.5.37 are congruent among the presented phylogenetic results, revealing a main evolutionary scenario (Figs. 13, 14). Despite the consistency of the taxonomic placement, the phylogenetic signal is characterized by the unusual combination of plateosaurid-like plesiomorphic traits combined with massopodan-like apomorphic features, which stands as an intermixtured, mosaic condition for the craniomandibular anatomy of SMF 13.5.37, as best highlighted above (see “morphological description of SMF 13.5.37”, “mosaic craniomandibular anatomy of SMF 13.5.37”, “supporting synapomorphies” in Supplementary material). It is noteworthy that a similar condition has been reported in the Argentinian *Coloradisaurus brevis* from the mid-to-late Norian of the Los Colorados Formation, which is generally referred as the oldest massospondylid known (Apaldetti et al., 2014; Pol et al., 2021). The development of a mosaic cranial anatomy mixing basal plesiomorphic and derived apomorphic characters in SMF 13.5.37 could have convergently evolved as an independent experimentation towards massopodan craniomandibular traits driven by speciation events in response to Laurasian paleoenvironmental settings comparable to those of Gondwanan continents. Alternatively, the condition of SMF 13.5.37 might be postulated as a morphological variation on a massopodan background pattern derived from Gondwanan taxa, like *Col. brevis*, that dispersed to Europe during the Late Triassic, with the acquisition of homoplastic features present in plateosaurids.

The resulting macroevolutionary scenario recovers SMF 13.5.37 as a basal massopodan, being either the basalmost member of Massopoda or the outgroup taxon of the clade formed by Massospondylidae + Sauropodiformes. Interestingly, the general position within Massopoda implicates that SMF 13.5.37 represents the first non-sauropodiform massopodan from Laurasia as well as the third Norian non-plateosaurid plateosaurian from Europe, together with *Sch. schutzi* and *Tue. maierfritzorum*. Remarkably, the massopodan affinity of SMF 13.5.37 is significant as it potentially fills the morphological gap between the more basal plateosaurids and the derived sauropodiforms from Europe during the Late Triassic, which otherwise would have been left vacant. As a further consequence, the recovery of SMF 13.5.37 within Massopoda might open up a plausible, though speculative, hypothesis of sauropodomorph dispersal towards the eastern regions of Laurasia during the Late Triassic, where a highly diverse vertebrate assemblage accounting exclusively for massospondylid and sauropodiform taxa was already fully established by the beginning of the Lower Jurassic in the Lufeng Basin (e.g. Barrett et al., 2005, 2007; Wang et al., 2017; Zhang et al., 2018, 2020). Moreover, the paucity of Triassic sauropodomorph material from Asia as well as the absence of bridging landmasses between eastern Laurasia and Gondwana, which were separated by the Tethys Ocean during the Triassic-Jurassic boundary (e.g. Scotese, 2014), strengthen the suggestion that the dominating massopodan clades in Asia might have originated from the already existing Triassic taxa of Europe, like plateosaurids or early-diverging massopodans, such as possibly SMF 13.5.37.

Analogously to the case of *Col. brevis*, whose postcranial material played a key role in elucidating its phylogenetic affinities given the conflicting signals of the cranial anatomy (Apaldetti et al., 2013, 2014), the postcranium of SMF 13.5 will be pivotal to better decipher its taxonomic placement within Massopoda. Despite the uncertainty concerning the precise taxonomic placement, SMF 13.5.37 increases the already wide craniodental disparity among basal sauropodomorphs (Ballell et al., 2022; Button et al., 2017), which is the result of a rapid evolutionary diversification aimed at opportunistically exploiting the diverse ecomorphological niches that were left vacant after the extinction of several herbivorous clades after the Carnian Pluvial Episode and prior to the Triassic-Jurassic boundary (Apaldetti et al., 2021; Bernardi et al., 2018; Marsh & Rowe, 2018; Pol et al., 2021). Furthermore, it is realistic to hypothesize that the massopodan cranial architecture was positively selected, likely due to its functional efficiency, and thus evolved in multiple lineages of Sauropodomorpha.

### Evolutionary trend across the late Norian

The Norian represents a crucial moment for the evolutionary history of Sauropodomorpha, as several lineages became rapidly established and successfully radiated across Pangea due to the gradual development of ecomorphological novelties, like herbivory and larger size, which subsequently led to the assembly of the typical sauropodomorph body plan by the end of this Late Triassic stage (e.g. Ezcurra et al., 2024; Marsh & Rowe, 2018; Müller, 2020; Pol et al., 2021). Consequently, numerous clades diversified by that time (plateosaurids, unaysaurids, riojasaurids, massospondylids, sauropodiforms), occupying disparate functional morphospace and experimenting unique combinations of features, especially related to their craniomandibular anatomy and locomotory system (e.g. Apaldetti et al., 2021; Ballell et al., 2022). Additionally, depending on the definition of the clade, also sauropods are referred to have originated before the Jurassic period (Sauropoda *sensu* Yates, 2007), given the presence of sauropod-like trackways from the Late Triassic of both Argentina and Greenland as well as osteological evidence of several large-sized, graviportal taxa, like lessemsaurids (Lallensack et al., 2017; Marsicano & Barredo, 2004; Pol et al., 2021).

A high diversity and an elevated abundance of sauropodomorphs from Norian-Rhaetian formations have been previously recorded in Gondwanan continents, especially South America, Africa and India, highlighting the taxonomic overlap of certain clades, mostly represented by early diverging massopodans and sauropodiforms, across several Southern Hemisphere regions (Apaldetti et al., 2021; Barrett et al., 2024; Ezcurra et al., 2024; Kutty et al., 2007; Novas et al., 2010; Pol et al., 2021; Rauhut et al., 2020). The discovery of SMF 13.5.37 as a non-*Plateosaurus* sauropodomorph with a mosaic craniomandibular anatomy leading towards a massopodan morphology extends and strengthens the evidence of a species-rich sauropodomorph assemblage also in the European continent, specifically regarding the Klettgau Formation of Switzerland which is thus similar to the Los Colorados Formation of Argentina (e.g. Pol et al., 2021) as well as the lower Elliot Formation of South Africa (e.g. McPhee et al., 2017). In this regard, a second comparable unit is the Trossingen Formation of Germany, which is also geographically proximate and stratigraphically congruent to the Swiss one (e.g. Regalado Fernández & Werneburg, 2022; Schaeffer, 2024; Yates, 2003a).

The presence of taxonomically comparable sauropodomorph assemblages distributed across Pangea subtends a major, globally-affecting evolutionary trend likely driven by dispersal first and subsequently by parallel development of adaptive features in response to similar paleonvironmental settings, heading to the definition of coeval, though geographically separated taxa that experimented similar morphological strategies and then occupied new regions of the functional morphospace, like *Col. brevis* and SMF 13.5.37.

It is noteworthy to highlight that an analogous evolutionary trend consisting of the development of derived novelties is reflected also in the coeval neotheropod *Not. frickensis* (Zahner & Brinkmann, 2019), which comes from the same stratigraphic horizon of SMF 13.5.37. The recovery of two saurischian dinosaurs showing a mosaic condition of plesiomorphic and apomorphic morphological features respectively of basal sauropodomorphs and theropods likely implies that the paleoenvironmental setting of Switzerland at Norian times played a decisive role in the evolution towards more derived clades. Nonetheless, more sedimentological analyses as well as the osteological descriptions of both the postcranium of SMF 13.5 and the newly found specimens from the uppermost fossiliferous horizon of the Klettgau Formation are essential to better understand the environmental selective pressures and the ecological mechanisms behind the establishment of a different dinosaurian fauna compared to the one of the lower horizons, which is rather dominated by *Pla. trossingensis* (e.g. Lallensack et al., 2021).

## Conclusions

The specimen SMF 13.5.37, a complete articulated skull associated to a partial skeleton of a new latest Norian sauropodomorph from Frick (SMF 13.5), is here reported to represent the first non-*Plateosaurus* sauropodomorph from the Canton Aargau and the fourth Late Triassic non-sauropodan sauropodomorph of Switzerland. The osteological investigation coupled with morphological comparisons unravelled a mosaic craniomandibular anatomy that combines features typical of non-massopodan plateosaurians and massopodan sauropodomorphs, a condition shared with the mid-to-late Norian massospondylid *Coloradisaurus brevis* from Argentina. Congruent with the osteological signature, the updated cladistic framework integrated with the novel, though incomplete, phylogenetic scoring of this new taxon unveiled a consistent taxonomic affinity for SMF 13.5.37, which is recovered as a massopodan sauropodomorph, branching out at the basal nodes of Massopoda, as well as the first non-sauropodiform massopodan from Laurasia. Remarkably, the resulting macroevolutionary scenario opens up a plausible hypothesis supporting a European origin for the Early Jurassic massopodans from Asia during the Late Triassic, although more evidence is required to corroborate it. Moreover, SMF 13.5.37 increases both the craniodental disparity and the paleobiodiversity of Norian sauropodomorphs from Laurasia, with the latter being comparable to those from Gondwana, especially South America and Africa.

In order to properly define the final phylogenetic placement of this new taxon and to further test the presented macroevolutionary implication, the description of the postcranium of SMF 13.5, which will be investigated elsewhere, is essential as it could alter its constituency and relationships. Furthermore, anatomical comparisons with *Schleitheimia schutzi*, *Gresslyosaurus ingens* and *Tuebingosaurus maierfritzorum*, known only from fragmentary postcranial specimens, will be needed to evaluate potential synonymousness with SMF 13.5, given the geographic proximity and the possible stratigraphic similarity of the three taxa, all of which assigned to the “latest Norian” (Rauhut et al., 2020; Regalado Fernández & Werneburg, 2022).

## Supplementary Information

Additional file1

Additional file2

Additional file3

Additional file4

## Data Availability

All data generated or analysed during this study are included in this published article and the fossil described herein is officially accessioned and available for study upon request at the Sauriermuseum Frick (SMF). The photogrammetry model of SMF 13.5.37 (in.obj format), the 3D surface models (in.ply format) of the segmented elements from the premaxillary block (i.e. partial premaxillae, partial right maxilla, partial dentaries and related teeth) and the underlying micro-computed tomography scans (µCT) are made available on Morphosource, repository project “Craniomandibular osteology of a new massopodan sauropodomorph (Dinosauria: Sauropodomorpha) from the Late Triassic (latest Norian) of Canton Aargau, Switzerland”, ID: 000699446 (https://www.morphosource.org/projects/000699446).

## Institutional abbreviations

AMNH FARB: American Museum of Natural History, Fossil Amphibian, Reptile and Bird collection, New York, New York, USA
CAPPA/UFSM: Centro de Apoio à Pesquisa Paleontologica da Quarta Colonia da Universidade Federal de Santa Maria, Rio Grande do Sul, Brazil
SMF: Sauriermuseum Frick, Frick, Canton Aargau, Switzerland
SMNS: Staatliches Museum für Naturkunde Stuttgart, Stuttgart, Germany

## Anatomical abbreviations

a: Angular
aof: Antorbital fenestra
aofs: Antorbital fossa
ar: Articular
bc: Basal constriction
bo: Basioccipital
bpp: Basipterygoid process
bs: Basisphenoid
bt: Basal tubera
clts: Caudolateral triangular spur
d: Dentary
dc: Distal carina
de: Denticles
dmn: Dorsomedial notch
emf: External mandibular fenestra
f: Frontal
fo: Foramen
fm: Foramen magnum
hy: Hyoid
idp: Interdental plates
idt: Isolated dentary tooth
itf: Infratemporal fenestra
j: Jugal
la: Lacrimal
Ld: Left dentary
Lmxt-III: Left maxillary tooth III
Lpmx-I: Left premaxillary tooth I
Lpmx-II: Left premaxillary tooth II
Lpmx-III: Left premaxillary tooth III
ls: Laterosphenoid
mc: Mesial carina
mid: Median internasal depression
Mkg: Meckelian groove
mr: Median ridge
mx: Maxilla
n: Nasal
nf: Narial fenestra
oc: Occipital condyle
or: Orbit
ot: Otoccipital
otp: Otoccipital triangular process
pa: Parietal
pac: Proatlas-atlas complex
pap: Paroccipital process
pf: Prefrontal
pmx: Premaxilla
po: Postorbital
pra: Prearticular
pt: Pterygoid
q: Quadrate
qj: Quadratojugal
Rd: Right dentary
Rdt: Right dentary tooth
s: Splenial
sa: Surangular
?sfo: ? Subnarial foramen
snfs: S-shaped nasal-frontal suture
so: Supraoccipital
sq: Squamosal
sr: Sclerotic ring
stf: Supratemporal fenestra
stfo: Supratemporal fossa
tr: Tooth root
uft: Undetermined fragment or tooth
