# Supplementary material for: Craniomandibular osteology of a new massopodan sauropodomorph (Dinosauria: Sauropodomorpha) from the Late Triassic (latest Norian) of Canton Aargau, Switzerland
Source: Swiss J Palaeontol. 2025 Jul 14;144(1):39. doi: 10.1186/s13358-025-00373-6 (PMC12321939; doi:10.1186/s13358-025-00373-6)
Supplement: Supplementary file 1 — Additional file1 [file 13358_2025_373_MOESM1_ESM.docx]

**SUPPLEMENTARY MATERIAL**

**Craniomandibular osteology of a new massopodan sauropodomorph (Dinosauria: Sauropodomorpha) from the Late Triassic (latest Norian) of Canton Aargau, Switzerland**

Alessandro Lania ^1*^, Ben Pabst ^2^ and Torsten M. Scheyer ^3^

^1^ Abteilung Paläontologie, Bonner Institut für Organismische Biologie, Rheinische Friedrich-Wilhelms-Universität Bonn, Nussallee 8, 53115, Bonn, Germany.

^2^ Sauriermuseum Aathal, Zürichstrasse 69, 8607 Aathal-Seegräben, Switzerland.

^3^ University of Zurich, Department of Paleontology, Karl-Schmid-Strasse 4, 8006 Zurich, Switzerland.

*Correspondence: Alessandro Lania, [alelania99@gmail.com](mailto:alelania99@gmail.com)

**SUPPLEMENTARY FILE**

The supplementary file corresponds to the modified data matrix from Ezcurra et al. (2024), which is available in NEXUS format for download.

**MEASUREMENT TABLE**

**Table 1**. Selected skull measurements (in cm) of SMF 13.5.37.

| **Cranial element** | **Anatomical feature** | **Right** | **Left** |
| --- | --- | --- | --- |
| Articulated skull | Rostrocaudal length (rostral tip of premaxilla to caudal end of articular) | 30.5 | 28.1 |
|  | Dorsoventral height (dorsal margin of supratemporal fenestra to ventral margin of angular) | 17.1 | 18.2 |
|  | Dorsoventral height of preorbital region (highest point anterior to the orbit to ventralmost margin of dentary) | 17.0 | 16.1 |
|  | Dorsoventral height of the cranium at the middle of the orbit | 8.6 | 9.7 |
|  | Mediolateral width (between the lateralmost edge of postorbitals in dorsal view) | 10.5 | |
|  | Mediolateral width at maximum transverse width of prefrontals | 8.6 | |
| External naris | Maximum rostrocaudal length | 8.5 | 7.2 |
|  | Maximum dorsoventral height | 5.9 | 5.1 |
| Antorbital fenestra | Maximum rostrocaudal length | 8.3 | 6.9 |
|  | Maximum dorsoventral height | 5.0 | 4.8 |
|  | Dorsoventral height of rostral margin | 4.0 | 5.5 |
|  | Dorsoventral height of caudal margin | 5.6 | 4.8 |
|  | Rostrocaudal length of dorsal margin | 3.9 | 0.8 |
|  | Rostrocaudal length of ventral margin | 8.3 | 6.9 |
| Orbit | Maximum rostrocaudal diameter | 6.1 | 5.8 |
|  | Maximum dorsoventral diameter | 8.6 | 9.7 |
| Supratemporal fenestra | Rostrocaudal length of longitudinal axis | 3.9 | 1.9 |
|  | Mediolateral length of transverse axis | 2.8 | 3.5 |
| Infratemporal fenestra | Rostrocaudal length of longitudinal axis | 3.7 | 2.3 |
|  | Dorsoventral length of frontal axis | 8.0 | - |
| Premaxilla | Rostrocaudal length | 8.3 | 7.8 |
|  | Rostrocaudal length of maxillary ramus | 3.0 | 2.4 |
|  | Rostrocaudal length of nasal ramus | 5.0 | 5.0 |
|  | Length of alveolar margin | 3.4 | 3.6 |
| Maxilla | Rostrocaudal length | 15.2 | 15.2 |
|  | Dorsoventral height (dorsal tip of nasal ramus to alveolar margin) | 5.2 | 5.5 |
|  | Rostrocaudal length of premaxillary ramus | 4.0 | 3.8 |
|  | Dorsoventral height of premaxillary ramus | 2.3 | 2.4 |
|  | Rostrocaudal length of nasal ramus | 4.7 | 5.4 |
|  | Rostrocaudal length of jugal ramus | 10.5 | 10.0 |
|  | Length of alveolar margin (rostralmost portion of the first tooth to the caudalmost portion of the last tooth) | 15.3 | 15.6 |
| Nasal | Rostrocaudal length | 13.6 | 16.2 |
|  | Dorsoventral height | 3.6 | 4.2 |
|  | Maximum width (median depression to overhanging bony lip forming the dorsal rim of the antorbital fenestra) | 4.4 | 4.6 |
|  | Rostrocaudal length of premaxillary ramus | 4.8 | 5.8 |
|  | Rostrocaudal length of maxillary ramus | 2.4 | 2.6 |
| Lacrimal | Rostrocaudal length of dorsal region | 1.6 | 1.9 |
|  | Dorsoventral height | 5.6 | 5.3 |
| Prefrontal | Rostrocaudal length of dorsomedial flange | 3.7 | 3.6 |
|  | Dorsoventral height (folding between dorsal flange and ventral ramus to distal tip of ventral ramus, following axis of ventral ramus) | 4.7 | 3.0 |
|  | Maximum transverse width | 2.3 | 2.4 |
| Frontal | Rostrocaudal length (along interfrontal suture) | 5.0 | |
|  | Mediolateral width (between distal end of each postorbital processes) | 6.8 | |
| Jugal | Rostrocaudal length | 9.5 | 11.2 |
|  | Dorsoventral height (along bisecting axis of dorsal ramus) | 4.6 | 5.3 |
|  | Minimum dorsoventral height below the orbit | 1.0 | 1.3 |
|  | Distance between the rostral end of the jugal (maxillary ramus) and the rostroventral corner of infratemporal fenestra | 5.5 | 8.0 |
|  | Rostrocaudal length of maxillary ramus | 4.3 | 6.9 |
|  | Rostrocaudal length of postorbital ramus | 3.8 | 4.4 |
|  | Rostrocaudal length of quadratojugal ramus | 6.1 | 4.8 |
| Postorbital | Rostrocaudal length | 4.4 | 5.1 |
|  | Dorsoventral height | 6.5 | 6.6 |
|  | Rostrocaudal length of jugal ramus | 4.7 | 4.1 |
|  | Rostrocaudal length of frontal ramus | 3.3 | 3.8 |
|  | Rostrocaudal length of squamosal ramus | 2.4 | 1.6 |
| Parietal | Rostrocaudal length (frontal-parietal suture to distal end of squamosal ramus) | 6.0 | |
|  | Mediolateral width (between distal end of squamosal rami) | 8.6 | |
|  | Mediolateral length of squamosal ramus | 4.7 | 4.9 |
| Squamosal | Rostrocaudal length | 4.5 | 4.2 |
|  | Dorsoventral height | 3.0 | 3.3 |
|  | Rostrocaudal length of postorbital ramus | 1.7 | - |
|  | Rostrocaudal length of parietal ramus | 1.5 | - |
| Quadratojugal | Rostrocaudal length (along rostroventral margin) | 4.3 | 3.5 |
|  | Dorsoventral height | 5.2 | 5.3 |
|  | Rostrocaudal length of jugal ramus | 4.2 | 3.2 |
|  | Dorsoventral length of squamosal ramus | 5.2 | 5.3 |
| Quadrate | Dorsoventral height | 10.1 | 8.3 |
| Braincase | Dorsoventral height (dorsal apex of supraoccipital to ventral edge of right basipterygoid process) | 11.4 | |
|  | Mediolateral width (between distal end of paroccipital processes) | 7.7 | |
|  | Mediolateral width across basal tuberae | 3.8 | |
|  | Mediolateral width of basisphenoid (between ventrolateral ends of basipterygoid processes) | 3.8 | |
| Articulated mandible | Rostrocaudal length (rostral end of dentary to caudal end of articular) | 27.5 | 27.0 |
|  | Dorsoventral height (dorsal margin of coronoid eminence to ventral border of angular) | 6.0 | - |
| External mandibular fenestra | Maximum rostrocaudal length | 4.1 | 3.5 |
|  | Maximum dorsoventral height | 3.2 | 2.9 |
| Dentary | Rostrocaudal length (rostral end to caudal end of caudoventral process) | 17.7 | 17.2 |
|  | Maximum dorsoventral height | 3.9 | 4.0 |
|  | Dorsoventral height of symphyseal region | 2.0 | 2.1 |
| Surangular | Rostrocaudal length | 14.4 | 12.7 |
|  | Dorsoventral height (dorsal margin of coronoid eminence to ventral margin at articulation with angular) | 4.4 | 5.0 |
| Angular | Rostrocaudal length | 8.7 | 8.2 |
|  | Dorsoventral height | 1.9 | 1.8 |
| Splenial | Rostrocaudal length (along ventral margin) | - | 10.9 |
| Prearticular | Rostrocaudal length | 5.1 | - |
| Articular | Rostrocaudal length (in medial view) | 5.5 | - |
|  | Mediolateral width | 2.4 | - |
|  | Rostrocaudal length of retroarticular process (in dorsomedial view) | 2.9 | 3.2 |
| Hyoid | Rostrocaudal length | 4.8 | - |

**SUMMARY LIST OF SCORING AMENDMENTS UPON THE PHYLOGENETIC MATRIX**

Given that the taxonomy of the species referred to the genus *Plateosaurus* is still controversial and one single type species is currently validly accepted, namely *Plateosaurus trossingensis* (ICNZ, 2019), the previously scored OTU “*Plateosaurus*” *gracilis* was accordingly changed into *Sellosaurus gracilis*, pending a thorough osteological revision of the associated material (e.g. Nau et al., 2020; Schaeffer, 2024). The codification of the OTU *Plateosaurus trossingensis* was updated based on the character scores of the redescription of the holotype SMNS 13200 (Schaeffer, 2024). Nonetheless, the scores for the cranial characters 8, 32, 50, 55, 71, 82, 84, 85 and 108 were kept from Ezcurra et al. (2024) as some of them either differ between the matrices (e.g. incongruent anatomical features, swapped scores, differently paraphrased) or were purposely omitted in the dataset from Schaeffer (2024) (i.e. characters 71 and 108). Finally, the sauropodomorph *Musankwa sanyatiensis* was added to the data matrix using the character scorings of Barrett et al. (2024).

Several scoring changes were defined based on information from the literature:

- Character 50, jugal contribution to the antorbital fenestra: present (0) or absent (1) (Holtz, 1994):
  - *Buriolestes schultzi*, *Coloradisaurus brevis* and *Lufengosaurus huenei* from “1” to “0”.
  - *Sarahsaurus aurifontanalis* and *Aardonyx celestae* from “?” to “0”.
- Character 58, frontal contribution to the supratemporal fenestra: present (0) or absent (1) (modified from Gauthier, 1986):
  - *Plateosaurus trossingensis* (pers. comm. Joep Schaeffer)*, Issi saaneq* and *Yunnanosaurus huangi* from “0” to “1”.
  - *Sellosaurus gracilis* from “0” to “?”.
- Character 65, length of jugal ramus of quadratojugal: no longer than (0), or longer than (1), the squamosal ramus (Wilson & Sereno, 1998):
  - *Buriolestes schultzi* and *Macrocollum itaquii* from “0” to “1”.
  - *Unaysaurus tolentinoi*, *Riojasaurus incertus* and *Coloradisaurus brevis* from “0” to “?”.

**CHARACTER SCORES FOR SMF 13.5.37**

? 0 0 1 1 0 0 2 1 ? 0 0 2 1 1 1 1 1 1 1 1 1 2 1 1 1 1 0 1 ? 0 0 1 1 0 0 0 1 1 ? 1 0 1 0 1 0 1 1 0 0 1 0 ? 1 1 1 1 0 0 0 ? ? ? 0 0 0 1 ? 0 1 ? ? ? ? 0 1 0 0 ? ? 1 0 0 ? 1 ? ? ? ? ? ? 1 ? 1 0 0 1 1 0 1 0 ? ? 0 1 1 0 1 1 1 0 1 0 1 0 1 0 0 0 0 ? ? ? ? ? ? ? ? ? ? ? ? ? ? ? ? ? ? ? ? ? ? ? ? ? ? ? ? ? ? ? ? ? ? ? ? ? ? ? ? ? ? ? ? ? ? ? ? ? ? ? ? ? ? ? ? ? ? ? ? ? ? ? ? ? ? ? ? ? ? ? ? ? ? ? ? ? ? ? ? ? ? ? ? ? ? ? ? ? ? ? ? ? ? ? ? ? ? ? ? ? ? ? ? ? ? ? ? ? ? ? ? ? ? ? ? ? ? ? ? ? ? ? ? ? ? ? ? ? ? ? ? ? ? ? ? ? ? ? ? ? ? ? ? ? ? ? ? ? ? ? ? ? ? ? ? ? ? ? ? ? ? ? ? ? ? ? ? ? ? ? ? ? ? ? ? ? ? ? ? ? ? ? ? ? ? ? ? ? ? ? ? ? ? ? ? ? ? ? ? ? ? ? ? ? ? ? ? ? ? ? ? ? ? ? ? ? ? ? ? ? ? ? ? ? ? ? ? ? ? ? ? ? ? ? ? ? ? ? ? ? ? ? ? ? ? ? ? ? ? ? ? 0 0 0 0 0 ? ? ? ? ? ? ? ? ? ? ? ? ? ? ? ? ? ? ? ? ? ? ? 0 0 0 ? 0 0 1 ? ? 1 ? 1 ? 0 ? ? 0 ? ? 0 1

**SUPPORTING SYNAPOMORPHIES OF SAUROPODOMORPHA NODES PRESENT IN SMF 13.5.37 AND UNIQUE COMBINATIONS OF CHARACTERS**

**Ezcurra et al. (2024): heuristic search under implied weights (K = 12)**

The synapomorphies supporting the basal nodes of both Plateosauria (i.e. the least inclusive node containing Plateosauridae, Unaysauridae and more derived plateosaurians) and Massopoda (i.e. the least inclusive node containing *S. ruessi* and more derived massopodans), as well as the second massopodan node (i.e. the least inclusive node containing Riojasauridae and more derived massopodans), are related to postcranial features, thus not detectable in SMF 13.5.37.

A single unambiguous synapomorphy supports the sister group relationship between SMF 13.5.37 and the massospondylid-sauropodiform clade:

- a slot-shaped subnarial foramen (character 14: 0 → 1).

On the other hand, SMF 13.5.37 is excluded from the massospondylid-sauropodiform clade because it lacks:

- lateral margin of nasal overhangs the antorbital fossa and forms its dorsal margin (character 23: 2 → 1);
- ascending ramus of the maxilla with an anteroposterior expansion at the dorsal end in lateral view (character 28: 0 → 1);
- development of the antorbital fossa on the ascending ramus weakly impressed and delimited by a rounded rim or a change in slope (character 31: 0 → 1);
- ratio of the minimum depth of the jugal below the orbit to the distance between the anterior end of the jugal and the rostroventral corner of the infratemporal fenestra greater than 0.2 (character 52: 0 → 1);
- length of jugal ramus of quadratojugal longer than the squamosal ramus (character 65: 0 → 1);
- distribution of the serrations along the mesial and distal carinae of the mid-posterior teeth restricted to the upper half of the crown (character 120: 0 → 1).

SMF 13.5.37 is distinguished from other massopodans due to the unique combination of the following cranial characters:

- antorbital fossa consisting of a large maxillary lamina, occupying more than 25% of the anteroposterior length of the antorbital opening, frequently with a straight to gently concave posterior margin (character 32: 0), shared with all non-massopodan plateosaurian, *C. brevis*, *A. celestae* and *M. readi*;
- neurovascular foramen at the posterior end of the lateral maxillary row opens ventrally (character 34: 1);
- orientation of the lacrimal orbital margin is erect and close to vertical (character 39: 1), shared with *J.* *xinwaensis*, *Y. sunae* and *Y. huangi*;
- maximum transverse width of the prefrontal more than 0.25 of the skull width at that level (character 45: 1), shared with *S. aurifontanalis* and *C. brevis*;
- frontal contribution to the supratemporal fenestra is present (character 58: 0), shared with *X. chengi*;
- height: length ratio of the dentary greater than 0.2 (character 98: 1), shared with *N. intloko* and *J.* *xinwaensis*;
- orientation of the maxillary tooth crowns is procumbent (character 110: 1), shared with *L. marayensis* and *A. mognai*;
- orientation of postorbital ramus of laterosphenoid extends laterally (character 414: 0), shared with *C. brevis*, *M. carinatus* and *A. mognai*.

**Ezcurra al. (2024): heuristic search under implied weights (K = 12) with characters 13 and 14 omitted**

The synapomorphies supporting the basal node of Plateosauria (i.e. the least inclusive node containing Plateosauridae, Unaysauridae and more derived plateosaurians) are based on the postcranial morphology and thus are not found in SMF 13.5.37 due to absence of anatomical overlap.

Three unambiguous synapomorphies supporting the basal node of Massopoda (i.e. the least inclusive node containing SMF 13.5.37 and other massopodans) are present in SMF 13.5.37:

- anteroposterior length of the antorbital fossa less than that of the orbit (character 29: 0 → 1);
- length of the posterior process of the prefrontal elongated so that total prefrontal length is equal to the anteroposterior diameter of the orbit (character 43: 0 → 1);
- anterior margin of the infratemporal fenestra extends under the rear half of the orbit (character 57: 0 → 1).

SMF 13.5.37 is excluded from the least inclusive clade that comprises Riojasauridae and more derived massopodans because it possesses:

- a web of bone spanning junction between anterior and ventral rami of lacrimal obscuring the posterodorsal corner of the antorbital fossa, otherwise absent in the latter monophyletic group (character 41: 1 → 0).

The unique combination of characters that differentiates SMF 13.5.37 from other massopodans partially matches the one provided with the analysis under implied weights with the characters 13 and 14 scored (see above, “Ezcurra et al. (2024): heuristic search under implied weights (K = 12)”), retaining most of them but switching the characters 32 and 58 with other two:

- orientation of the paroccipital process in occipital view is slightly dorsolaterally directed to horizontal (character 77: 0), shared *S. aurifontanalis* and *N. intloko*;
- presence of a stout, triangular, medial process of the articular, behind the glenoid (character 104: 0), shared with *C. brevis, X. chengi* and *J.* *xinwaensis*.

**SUPPLEMENTARY FIGURES**

**Figure S1.** Strict consensus tree recovered from the heuristic searches under equal weights and equal weights with characters 13 and 14 omitted of the modified matrix from Ezcurra et al. (2024). Node numbers: 1, Dinosauria; 2, Sauropodomorpha.

**Figure S2.** Three-dimensional rendering of the segmented snout of SMF 13.5.37. **A)** Rostral view. **B)** Caudal view. **C)** Left lateral view. **D)** Right lateral view. **E)** Dorsal view. **F)** Ventral view. For abbreviations, see main text (“anatomical abbreviations”). Scale bar equals 2 cm.

**ADDITIONAL COMMENT ON NAMING A NEW TAXON BASED ON SMF 13.5.37**

Despite SMF 13.5.37 unquestionably represents the skull of new massopodan sauropodomorph based on both its unique craniomandibular anatomy and the presented phylogenetic results, the name of the new taxon was preferred not to be assigned in this paper. Accordingly, the phylogenetic signal of the taxon is incomplete as the postcranium of the skeleton SMF 13.5 still awaits an osteological description, as well as other partial specimens tentatively referred to the same new massopodan sauropodomorph, which might be pivotal to better decipher its phylogenetic affinities (see main text, “phylogenetic analysis”). Finally, the postcranial series will be crucial to test potential synonymousness with stratigraphically comparable taxa which lack cranial material.

To wit, a valid taxon name will be defined together with a complete emended diagnosis only after a thorough osteological description and codification of the undescribed referred material, following the establishment of a holotypic specimen and paratype.

**REFERENCES**

Barrett, P.M., Chapelle, K.E.J., Sciscio, L., Broderick, T.J., Zondo, M., Munyikwa, D., & Choiniere, J.N. (2024). A new Late Triassic sauropodomorph dinosaur from the Mid-Zambezi Basin, Zimbabwe. *Acta Palaeontologica Polonica, 69*(2), 227–241. https://doi.org/10.4202/app.01100.2023.

Ezcurra, M.D., Müller, R.T., Novas, F.E., & Chatterjee, S. (2024). Osteology of the sauropodomorph dinosaur *Jaklapallisaurus asymmetricus* from the Late Triassic of central India. *The* *Anatomical record*, *307*(4), 1093–1112. https://doi.org/10.1002/ar.25359.

Gauthier, J.A. (1986). Saurischian monophyly and the origin of birds. In Padian, K. (Ed.), *Memoirs of the California Academy of Sciences* (*8*, pp. 1-55). San Francisco: The Academy.

Holtz, T. R. (1994). The phylogenetic position of Tyrannosauridae: implications for theropod systematics. *Journal of Paleontology*, *68(5)*, 1100–1117. https://doi.org/10.1017/S0022336000026706

ICZN. (2019). Opinion 2435 (Case 3560) – *Plateosaurus* Meyer, 1837 (Dinosauria, Sauropodomorpha): new type species designated. *The Bulletin of Zoological Nomenclature*, *76*(1), 144-145. https://doi.org/10.21805/bzn.v76.a042.

Nau, D., Lallensack, J.N., Bachmann, U., & Sander, P.M. (2020). Postcranial osteology of the first early-stage juvenile skeleton of *Plateosaurus trossingensis* from the Norian of Frick, Switzerland. *Acta Palaeontologica Polonica*, *65*(4), 679–708. https://doi.org/10.4202/app.00757.2020.

Schaeffer, J. (2024). Osteological redescription of the holotype of *Plateosaurus trossingensis* (Dinosauria: Sauropodomorpha) from the Upper Triassic of SW Germany and its phylogenetic implications. *Journal of Systematic Palaeontology*, *22*(1). https://doi.org/10.1080/14772019.2024.2335387.

Wilson, J.A., & Sereno, P.C. (1998). Early Evolution and Higher-Level Phylogeny of Sauropod Dinosaurs. *Journal of Vertebrate Paleontology*, *18*(sup002), 1–79. https://doi.org/10.1080/02724634.1998.10011115.

Yates, A.M. (2007). The first complete skull of the Triassic dinosaur *Melanorosaurus* Haughton (Sauropodomorpha: Anchisauria). *Special Papers in Palaeontology*, *77*, 9–55.
