# Supplementary figures and images for: Craniomandibular osteology of a new massopodan sauropodomorph (Dinosauria: Sauropodomorpha) from the Late Triassic (latest Norian) of Canton Aargau, Switzerland
Source: Swiss J Palaeontol. 2025 Jul 14;144(1):39. doi: 10.1186/s13358-025-00373-6 (PMC12321939; doi:10.1186/s13358-025-00373-6)

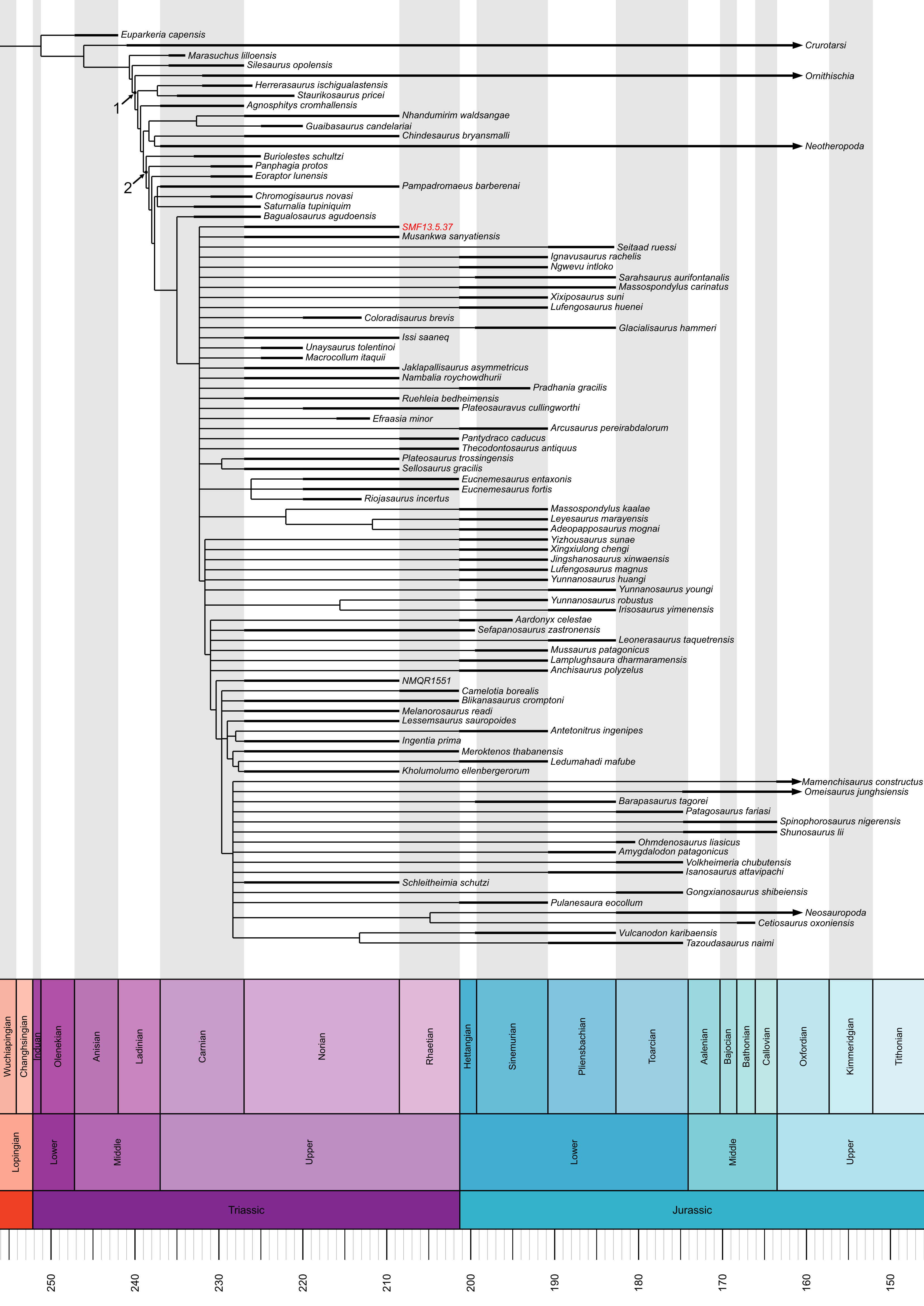

Supplement: Supplementary file 3 — Additional file3 [file 13358_2025_373_MOESM3_ESM.jpg]

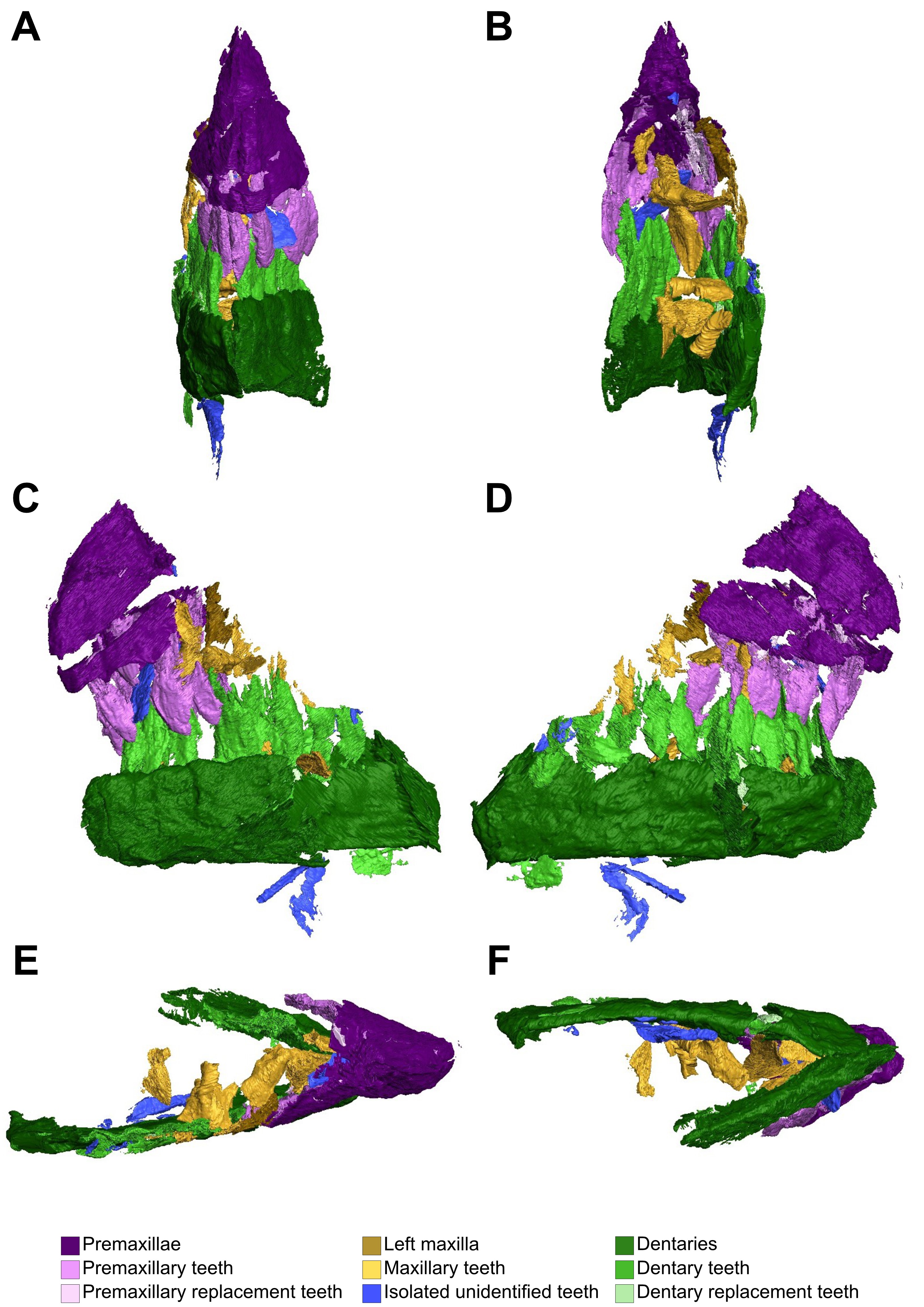

Supplement: Supplementary file 4 — Additional file4 [file 13358_2025_373_MOESM4_ESM.jpg]
